# Congenericity of Claimed Compounds in Patent Applications

**DOI:** 10.3390/molecules26175253

**Published:** 2021-08-30

**Authors:** Maria J. Falaguera, Jordi Mestres

**Affiliations:** 1 Research Group on Systems Pharmacology, Research Program on Biomedical Informatics (GRIB), IMIM Hospital del Mar Medical Research Institute, Parc de Recerca Biomèdica (PRBB), Doctor Aiguader 88, 08003 Barcelona, Catalonia, Spain; 2 Department of Experimental and Health Sciences, University Pompeu Fabra, Parc de Recerca Biomèdica (PRBB), Doctor Aiguader 88, 08003 Barcelona, Catalonia, Spain; 3 Chemotargets SL, Baldiri Reixac 4, Parc Cientific de Barcelona, 08028 Barcelona, Catalonia, Spain

**Keywords:** chemical series, patent compounds, similarity analysis, SureChEMBL, SureChEMBLccs, ChEMBL

## Abstract

A method is presented to analyze quantitatively the degree of congenericity of claimed compounds in patent applications. The approach successfully differentiates patents exemplified with highly congeneric compounds of a structurally compact and well defined chemical series from patents containing a more diverse set of compounds around a more vaguely described patent claim. An application to 750 common patents available in SureChEMBL, SureChEMBLccs and ChEMBL is presented and the congenericity of patent compounds in those different sources discussed.

## 1. Introduction

A chemical series is a central concept in drug discovery that defines a set of small molecules sharing a core structure decorated with different functionalities [1,2,3,4,5]. These molecular analogues effectively map the chemical space around their common scaffold and thus, they constitute the basis to explore structure-activity relationships [6] and to identify activity cliffs [7,8,9], such as in cases of structurally similar compounds with large differences in binding affinities for a given target. When chemical series are enriched with molecules active against several members of a target family their common molecular framework is referred to as a privileged scaffold [10,11,12,13,14,15], one of the basic principles in chemogenomics initiatives [16,17,18].

The development of unsupervised computational protocols for the identification of chemical series in large compound collections has become, in recent years, an active area of research in chemoinformatics [19,20,21]. Applications capable of automatically detecting the core chemical structure of patent claims among all chemical entities stored in patent databases [22,23] have received special attention [24,25,26]. Due to the use of chemical entity recognition technologies to extract all small molecules from patent text and images, the main difficulty in these cases lies in distinguishing the exemplified compounds intended to be protected from many other starting materials and intermediate products mentioned in the patent. Under the assumption that claimed molecules fit into a chemical series defined by the Markush structure of the patent claim, recent computational protocols have exploited this concept to successfully extract and make publicly available all pharmacologically relevant molecules contained in the largest patent database [27].

Having the ability to automatically identify the chemical series of claimed compounds in patents, one may then wonder how well the chemical space around the patent claim is covered by those exemplified compounds in the patent. In essence, what we are interested in here is defining a set of quantitative parameters to assess the degree of congenericity of patent compounds. A highly congeneric chemical series of exemplified compounds will be associated with a narrow protection of a well-defined portion of the chemical space, whereas a set of exemplified compounds with low congenericity may reflect a loose attempt to cover the chemical space defined by the patent claim, offering opportunities to fill in the remaining gaps.

## 2. Database and Methods

### 2.1. SureChEMBLccs

We used a subset of the SureChEMBLccs 2021 release [27] that includes 159,439 unique small molecules from 851 US pharmacological and high confidence patents [26] of which 47,857 molecules are also present in ChEMBL [28]. Of those 851 patents, 750 have more than one molecule in ChEMBL. A patent is described as pharmacological when it has an A61K* IPC code, with the exception of A61K6 (preparations for dentistry), A61K7 or A61K8 (cosmetics or similar toilet preparations), A61K9 (medicinal preparations characterized by special physical form), A61K38 (medicinal preparations containing peptides), A61K39 (medicinal preparations containing antigens or antibodies) and A61K48 (medicinal preparations containing genetic material which is inserted into cells of the living body to treat genetic diseases). Additionally, a patent is considered of high confidence when the molecules exemplified in it describe a similarity distribution with a median value equal or higher than 0.4.

### 2.2. Similarity Distribution Descriptors

A list of three descriptors is suggested to be used in congenericity analyses of sets of molecules. They can be computed from any of the pairwise similarity distributions derived for a given set of N molecules. One intuitive approach is to base the construction of the similarity distribution on the prior selection of a reference molecule. In principle, any molecule can be used as a reference. In this work, a *centroid* is selected as the reference molecule having the maximum value of its minimum pairwise similarity. The *centroid similarity distribution* is then defined as the counts of pairwise Dice similarities between the Morgan fingerprints of the centroid against the other N-1 molecules in the set, calculated with RDKit [29], within each hundredth of the 0.00 to 1.00 range of similarity values. Alternatively, one can avoid the reference compound selection and directly compute the similarities between all N × (N − 1)/2 unique pairs of non-identical molecules to construct an *all pairwise similarity distribution*. Then, the shape of the centroid or all pairwise similarity distribution will be quantitatively characterized by its *extent*, *mode*, and *density*. The *extent* is given by the minimum similarity value populated in the distribution. The *mode* is the most frequent similarity value in the distribution. Finally, the *density* measures the degree of dispersion around the *mode*. It is obtained by subtracting from unity the normalized projected Shannon entropy of the distribution [30], that is, the projected optimal number of uniformly occupied bins. A set of compounds having a similarity distribution with high extent, high mode, and high density values will be associated with a highly congeneric series. An additional fourth descriptor, *size*, is considered to account for the number of molecules in the set.

## 3. Results and Discussion

### 3.1. Use of Similarity Distribution Descriptors on Illustrative Patent Applications

**Figure 1:**
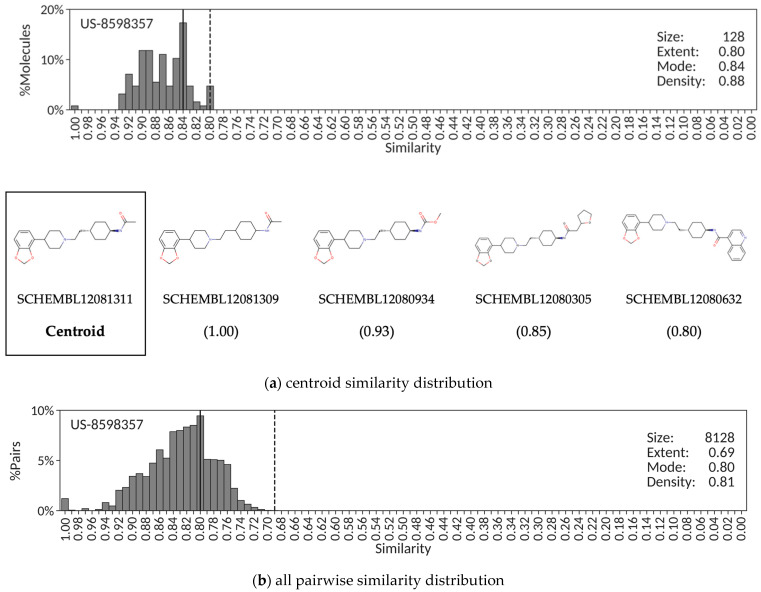
Centroid (**a**) and all pairwise (**b**) similarity distributions for the set of exemplified molecules in SureChEMBLccs patent US-8598357. Additionally, included (**a**) is a sample of chemical structures with their SCHEMBL identifiers and similarity values (in parenthesis). The extent and mode are indicated, respectively, by vertically dashed and solid lines.

A set of representative examples were selected to illustrate the use of similarity distribution descriptors to perform congenericity analyses of exemplified compounds in patent applications. The first example is patent US-8598357, a typical case of a patent containing a highly congeneric series of 128 benzodioxole piperidine compounds claimed as dual modulators of the serotonin 2A and dopamine D3 receptors. Figure 1a shows the distribution of pairwise similarity values of all exemplified molecules against the selected centroid, SCHEMBL12081311. The high extent (0.80), high mode (0.84) and high density (0.88) values obtained are all consistent with a highly congeneric chemical series of patent molecules. Correspondingly, the distribution obtained from all 8128 pairwise similarities (Figure 1b) results also in high extent (0.69), high mode (0.80) and high density (0.81) values. This situation will occur when the core structure common to all exemplified compounds in a patent application covers a large portion of the chemical structures, and most molecules differ only by rather small functional groups at one edge of that core structure. This conclusion is confirmed by visual inspection of a selection of molecules with similarity values covering the entire range of the extent in the centroid similarity distribution (Figure 1a).

**Figure 2:**
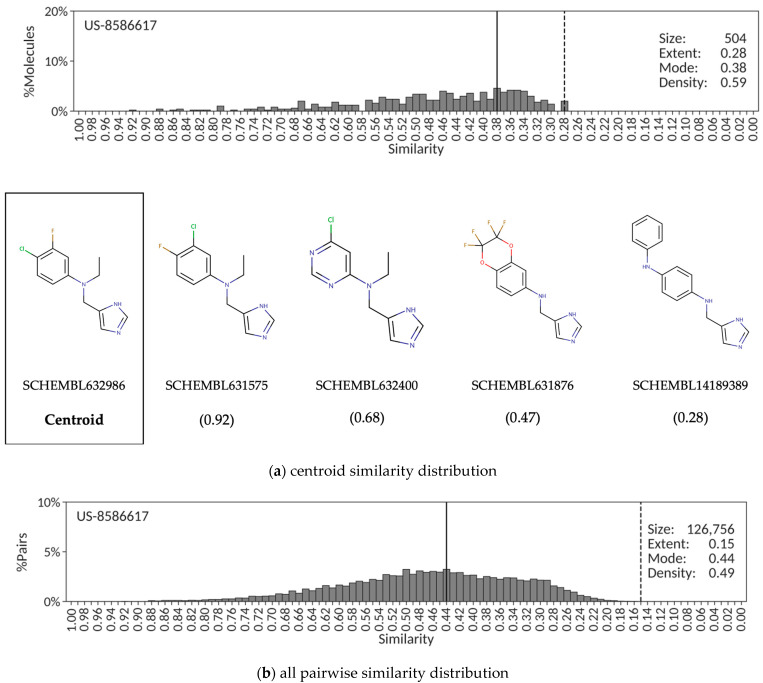
Centroid (**a**) and all pairwise (**b**) similarity distributions for the set of molecules exemplified in SureChEMBLccs patent US-8586617. Additionally, included (**a**) is a sample of chemical structures with their SCHEMBL identifiers and similarity values (in parenthesis). The extent and mode are indicated, respectively, by vertically dashed and solid lines.

A completely different scenario is found in patent US-8586617 protecting a chemical series of 504 amino-4-methyl imidazoles for the treatment of depression, anxiety and bipolar disorders, among others. In this case, the centroid similarity distribution around SCHEMBL632986 is quantitatively characterized by low extent (0.28), low mode (0.38) and medium density (0.59) values consistent with a chemical series of patent molecules aiming at sampling diversity rather than coverage completeness (Figure 2a). Similarly, the corresponding distribution derived from all 126,756 pairwise similarities (Figure 2b) results in low extent (0.15), low mode (0.44) and low density (0.49) values. In contrast to the previous patent example, this situation is likely to occur when the common core structure covers only a minor portion of the chemical structure in most of the exemplified compounds in the patent that contains a wide range of diverse, and often large, functionalities around it. This inference is substantiated by the selection of molecules with similarity values covering the entire range of the extent in the centroid similarity distribution (Figure 2a).

**Figure 3:**
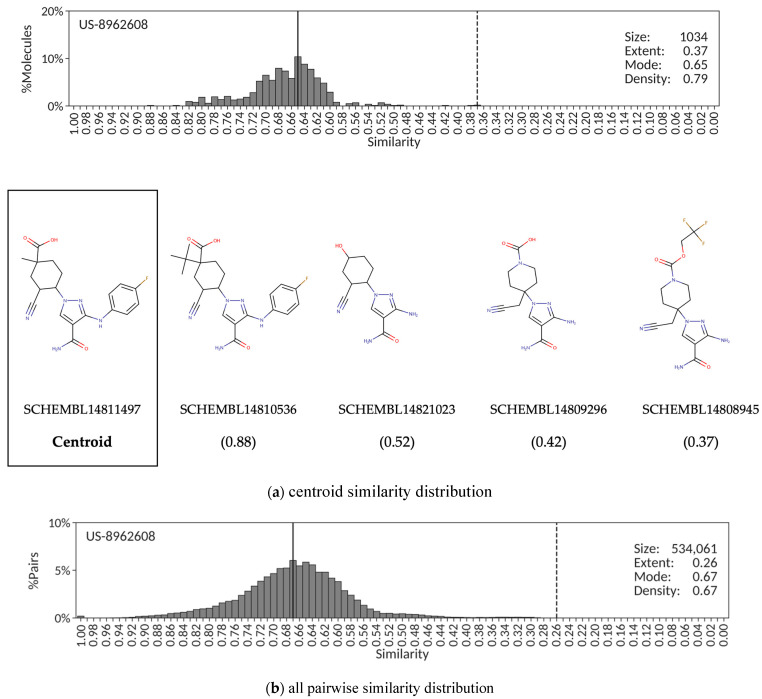
Centroid (**a**) and all pairwise (**b**) similarity distributions for the set of molecules exemplified in SureChEMBLccs patent US-8962608. Additionally, included (**a**) is a sample of chemical structures with their SCHEMBL identifiers and similarity values (in parenthesis). The extent and mode are indicated, respectively, by vertically dashed and solid lines.

An intermediate situation between the two cases presented above is provided by patent US-8962608 aiming at protecting cycloalkylnitrile pyrazole carboxamides as Janus kinase inhibitors. Most of the 1034 exemplified molecules extracted from this patent show, in fact, relatively high similarity values (>0.60) against the centroid, SCHEMBL14811497; however, a small number of molecules form a long tail below that similarity mark with values as low as 0.37 (Figure 3a). Therefore, despite the medium mode (0.65) and high density (0.79) values, the centroid similarity distribution also has a low extent (0.37) value. The corresponding distribution derived from all 534,061 pairwise similarities (Figure 3b) follow very much the same trend, with a low extent (0.26) value despite the relatively high mode (0.67) and density (0.67) values. This may help in the unsupervised identification of patents for which a large subset of the exemplified compounds extracted automatically form a reasonably tight congeneric series; however, this congenericity is somehow masked with medium to low extent values due to the presence of a few distant compounds that nonetheless share some core structure attributes. As can be observed in Figure 3a, this is indeed the case for this patent because some intermediate products were recognized by the automatic extracting protocol [26] as being part of the core structure of claimed compounds (SCHEMBL14821023, SCHEMBL14809296 and SCHEMBL14808945).

The shape of similarity distributions and the descriptor values derived from them depend ultimately on the choice of the reference compound. In the patent examples presented above, the similarity centroid was selected as the reference compound. To assess the dependency of the similarity distribution descriptors on the selected reference, a random sample of 10% of all compounds from each patent was extracted, each compound selected as individual reference, and the corresponding similarity distributions and associated descriptors calculated. The results reveal that, even though the exact values of extent, mode and density may vary slightly, the overall quantitative description of the different similarity distributions obtained from random claimed compounds in a patent is essentially retained. Accordingly, the corresponding mean and standard deviation values for extent, mode and density using 13 random compounds as references to derive the similarity distributions of patent US-8598357 are 0.74 ± 0.02, 0.82 ± 0.02 and 0.86 ± 0.02, respectively, not too distant from the values reported in Figure 1a. Similarly, the extent, mode and density values calculated from the similarity distributions constructed when using a set of 50 random claimed compounds from patent US-8586617 are 0.21 ± 0.03, 0.46 ± 0.10 and 0.61 ± 0.06, respectively, all close to the values shown in Figure 2a, and those resulting from taking 103 random compounds from patent US-8962608 are 0.31 ± 0.02, 0.65 ± 0.07 and 0.76 ± 0.04, respectively, all values near those reported in Figure 3a. Therefore, even though similarity distribution descriptors depend on the reference compound selected, the variability observed in their exact values does not affect the ability of the descriptors to capture quantitatively the essence of the degree of congenericity in sets of claimed patent compounds. 

The alternative to using a reference molecule to construct the similarity distribution is to simply account for all pairwise similarities. The advantages are that it alleviates the reference compound selection dilemma, and it provides a unique, more robust, similarity distribution. However, there are also some disadvantages worth considering. For example, the selection of a centroid from which the similarity distribution is derived offers a sense of chemical space coverage around a molecular structure central to the set of patent molecules that cannot be obtained from an all pairwise similarity distribution. Additionally, one may argue that plotting the percentage of molecular pairs instead of the percentage of molecules in the corresponding similarity distributions provides a less intuitive picture of the similarities between molecules and may confound comparisons between patents. Balancing all these advantages and disadvantages and also considering the good correspondence between descriptor values obtained from centroid and all pairwise similarity distributions observed in the three patent examples presented above, centroid similarity distributions will be used in the remainder of this work.

### 3.2. Correlations of Similarity Distribution Descriptors across Patents

Having illustrated the use of similarity distribution descriptors to quantify the degree of congenericity of claimed compounds in three patent examples, the set of 851 US pharmacological and high confidence SureChEMBLccs patents present also in ChEMBL was processed. In terms of size, the number of claimed molecules per patent ranged from 2 to 2790, with a median value of 98 molecules, and 425 (50%) and 785 (92%) of the patents containing more than 100 and less than 600 molecules, respectively. The analysis of the centroid similarity distributions gave a wide range of extent values, ranging from 0.09 to 1.00, with a median value of 0.50, and of mode values from 0.12 to 0.96, with a median value of 0.67. It is worth mentioning here that the limit case of extent values equal to unity is due to seven patents (e.g., US-8895245) having two molecules (e.g., SCHEMBL804176 and SCHEMBL803938) with identical Morgan fingerprints but different structures. In contrast, density values tended to be relatively high for all 851 patents, with minimum and median values of 0.53 and 0.78, respectively. Overall, the median values obtained for the three similarity distribution descriptors reflect the fact that patent molecules in SureChEMBLccs form rather compact chemical series around common core chemical structures and provide further reassurance of the filtering protocol applied to extract them from the original SureChEMBL database [26].

**Figure 4:**
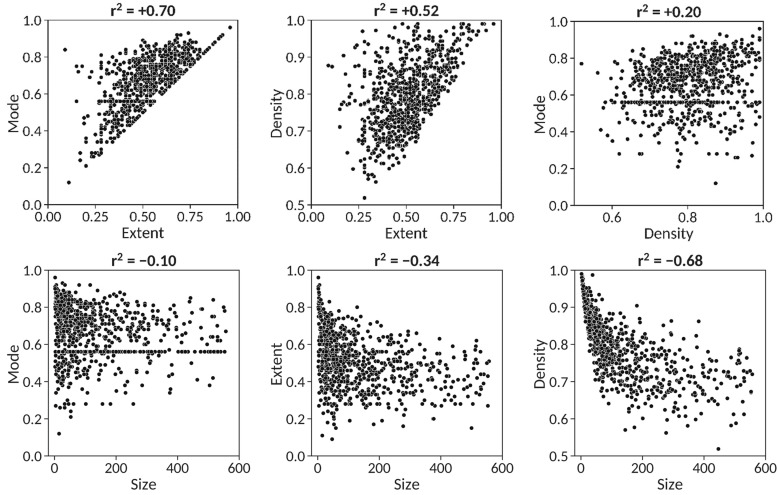
Pairwise correlations between the four descriptors obtained from centroid similarity distributions of 851 patents. The correlation coefficient (r^2^) is provided on top of each graph.

Examination of the potential existence of pairwise correlations between the descriptors obtained for the 851 patents resulted in the identification of both positive and negative correspondences. As shown in Figure 4, the strongest correlation identified (r^2^ = +0.70) is between extent and mode, a trend exposing that, on one hand, patents having high extent values necessarily accumulate pairs of molecules at high mode values (see Figure 1) and, on the other hand, as extent values decrease, the similarity distribution tends to disperse its bin population across the extent and thus, the mode values have also a tendency to decrease accordingly. In this respect, almost 79% of the patents (669) have minimum similarity values (extent) within 0.25 orders of magnitude from the mode indicating that patents with high modes and low extents are exceptional. The second strongest correlation, albeit negative (r^2^ = −0.68), is between density and size. This is an expected situation as the larger the number of claimed molecules in a patent, the larger its chemical diversity in principle is and thus, the more difficult that pairwise similarities accumulate around the mode, resulting in lower density values. The third trend encountered (r^2^ = +0.52) is between extent and density, which is consistent with the fact that molecular sets having high minimum pairwise similarities (high extent) are more likely to have their similarity distributions concentrated in a small number of bins (high density) and vice versa. No significant relationships were found between extent and size, mode and size and mode and density.

**Figure 5:**
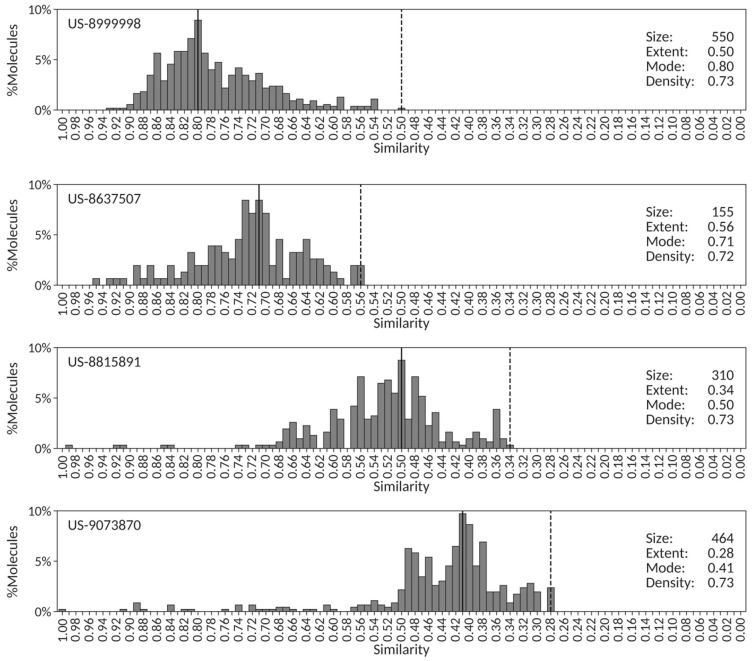
Centroid similarity distributions for four patents with almost identical density values. The mode and the extent are indicated, respectively, by vertically solid and dashed lines.

To illustrate these correlations with concrete examples, a set of four patents was selected with consistent high density values (0.73) and varying extent, mode and size values. Their corresponding centroid similarity distributions are shown in Figure 5. The first patent (US-8999998) contains a highly congeneric chemical series of 550 pyrazolopyrimidine Janus kinase inhibitors, with a centroid similarity distribution characterized by a medium extent (0.50) and a high mode (0.80). The second patent (US-8637507) is composed of 155 heterocyclic compounds as diacylglycerol acyltransferase inhibitors that has a centroid similarity distribution of comparable density to US-8999998 but with slightly higher extent (0.56) and lower mode (0.71). The third (US-8815891) and fourth (US-9073870) patents exemplify, respectively, 310 tricyclic derivatives as poly(ADP-ribose) polymerase inhibitors and 464 alicyclic carboxylic acid derivatives of benzomorphans and related scaffolds as 11b-hydroxysteroid dehydrogenase 1 inhibitors. Despite having consistent density values with the first two patents, their centroid similarity distributions have clearly lower extent (0.34 and 0.28, respectively) and lower mode (0.50 and 0.41, respectively) values consistent with sets of more diverse compounds that nonetheless share a core chemical structure [26].

The shape of the four centroid similarity distributions shown in Figure 5 is representative of the average similarity distribution obtained for the set of 851 SureChEMBLccs patents analyzed in this work, with average density values of 0.78 (*vide supra*). Comparing the values of the descriptors across the four patents, the positive trend between extent and mode detected above (Figure 4) is recovered and can be visually assessed. It becomes evident that no clear trend can be established for the remaining descriptor pairs,

As a final remark, it is worth stressing that the level of precision in the wording of the patent summary defining the chemical nature of the compounds being claimed already provides some clues on the expected degree of congenericity for the set of exemplified compounds in those patents. For example, defining a set of pyrazolopyrimidine inhibitors in patent US-8999998 is a more chemically precise wording than the generic mention of alicyclic carboxylic acid derivatives of benzomorphans and related scaffolds in patent US-9073870, and this is then clearly reflected in the differences between extent (0.50 vs. 0.28) and mode (0.80 vs. 0.41) values. This aspect could be exploited in the use of text-mining techniques when processing patent titles and summaries.

### 3.3. Congenericity Analysis of SureChEMBL Patents

SureChEMBLccs [27] was derived by applying an unsupervised automatic filtering protocol to identify the core chemical structure in SureChEMBL patents and extract all pharmacologically relevant molecules exemplifying the patent claims [26]. Accordingly, SureChEMBLccs should be in principle intrinsically biased towards a highly congeneric chemical series of compounds. To assess this assumption and validate the use of similarity distribution descriptors to quantify congenericity in sets of molecules, a principal component analysis (PCA) was performed on a focused set of 750 SureChEMBL patents for which a filtered subset of compounds sharing a core chemical structure is available in SureChEMBLccs and a carefully curated selection of at least two compounds is also present in ChEMBL. In PCA, each patent was quantitatively defined by the extent, mode and density values of the centroid similarity distributions derived from the corresponding full set of compounds in SureChEMBL, SureChEMBLccs, and ChEMBL.

**Figure 6:**
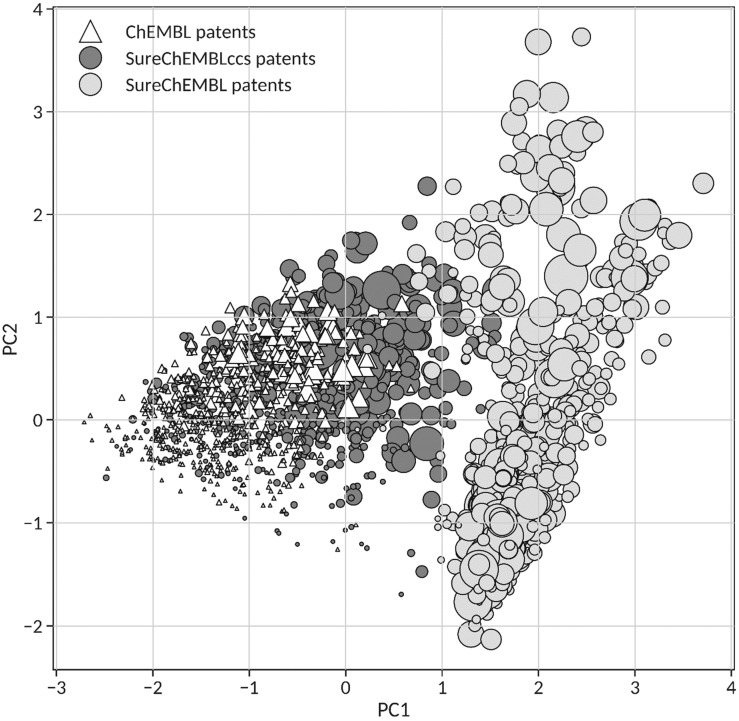
Principal Component Analysis of 750 patents based on the corresponding molecules available in ChEMBL (white triangles), SureChEMBLccs (dark grey circles) and SureChEMBL (light grey circles). PC1 and PC2 describe 75% and 23% of the total variance, respectively.

Figure 6 shows the projection of the 750 common patents between SureChEMBL (light grey circles), SureChEMBLccs (dark grey circles) and ChEMBL (white triangles) on the first two principal components that combined accumulate 98% of the variance (PC1 75% and PC2 23%). The loadings of the extent, mode and density values in PC1 (−0.65, −0.59 and −0.48, respectively) reveal a major contribution of extent and mode values in the first principal component, whereas the corresponding loadings in PC2 (0.14, 0.53 and −0.84, respectively) denote a major contribution of density values. The fact that PC1 describes already 75% of the variance allows the ordering of patents from all sources according to their intrinsic congenericity from left to right in the PC1 axis. Indeed, as can be observed, there is clear separation between the highly congeneric sets of claimed molecules assigned to all 750 patents in ChEMBL and SureChEMBLccs, on the left (PC1 < 1), and all molecules originally extracted from those patents and deposited in SureChEMBL, on the right (PC1 > 1). The strong presence of starting materials and intermediate products in SureChEMBL is certainly responsible for the low degree of congenericity associated with the full set of patent compounds in SureChEMBL. As clearly visible in Figure 6, this situation was corrected in SureChEMBLccs through the automatic identification of those molecules in the patent sharing a core chemical structure [26], resulting in sets of patent compounds with significantly higher congenericities, comparable to those observed for the curated sets contained in ChEMBL. In this respect, it ought to be stressed that the size values for the 750 patents in ChEMBL range from 2 to 787 molecules per patent with a median value of 23, significantly smaller than the median size of 111 molecules in SureChEMBLccs for those same 750 patents. In fact, the number of claimed compounds per patent in SureChEMBLccs is on average 7.5 times larger than in ChEMBL. Therefore, the fact that SureChEMBLccs overlap well with ChEMBL for 750 patents (Figure 6) provides confidence for the high degree of congenericity of the chemical series for all 188,795 pharmacological patents available in SureChEMBLccs [26].

**Figure 7:**
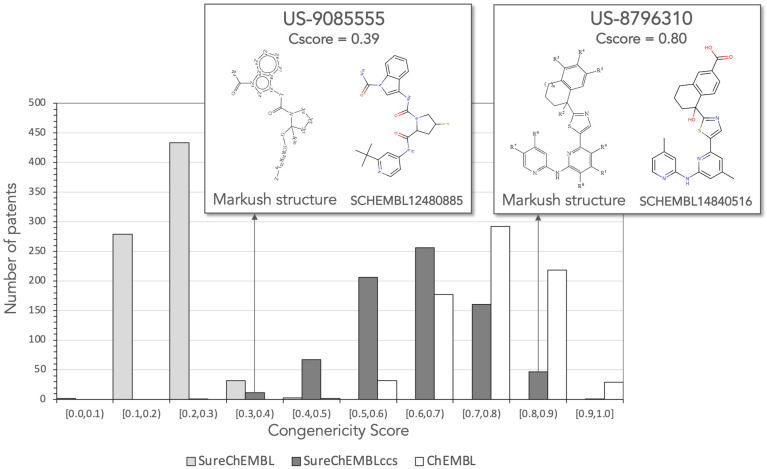
Congenericity Score (CScore) distributions for the same 750 patents in SureChEMBL, SureChEMBLccs and ChEMBL.

Given the optimal split obtained between patents in ChEMBL and SureChEMBLccs, on one side, and in SureChEMBL, on the other side, a congenericity score (CScore) was defined as the geometric mean of the three similarity distribution descriptors used in the PCA. The distribution of CScores from the molecules available in the three patent sources for the set of 750 patents is presented in Figure 7. As can be observed, all patents in SureChEMBL (except three) have CScores below 0.4. In contrast, all patents in ChEMBL and 737 patents (98%) in SureChEMBLccs obtained CScores above 0.4. Therefore, a CScore threshold of 0.4 is recommended to assume a minimum degree of congenericity within patent molecules.

To illustrate the difference between patents containing a highly congeneric set of compounds and patents exemplified with more diverse chemical structures, two patent examples from SureChEMBLccs having CScores above and below 0.4 are included in Figure 7. Patent US-8796310 refers to the invention of amino-pyridine-containing compounds as spleen tyrosine kinase (SYK) inhibitors. The centroid of the patent molecules in SureChEMBLccs is compound SCHEMBL14840516 and its structure matches perfectly the Markush structure of the patent claim. The extent, mode and density values of its centroid similarity distribution are 0.72, 0.87 and 0.81, respectively. The resulting CScore of 0.80 reflects that molecules exemplified in this patent do not deviate much from the Markush structure. Conversely, patent US-9085555 has a CScore of 0.39 in SureChEMBLccs, right below the recommended CScore threshold of 0.4. The patent claims a set of compounds around a Markush structure that allows a wide diversity of ring sizes and composition, linkers and functional groups. The centroid of the filtered patent molecules in SureChEMBLccs is compound SCHEML12480885 and the similarity distribution constructed around it returned extent, mode and density values of 0.25, 0.43 and 0.56, respectively. Based on these results, CScore values offer a good simple metric to assess the congenericity of claimed compounds in patent applications.

Finally, it ought to be stressed that, very much in agreement with the results presented above (Figure 1, Figure 2 and Figure 3), a strong correlation (r^2^ = +0.93) was found between the CScores calculated from the extent, mode and density values obtained from centroid similarity distributions and all pairwise similarity distributions. Therefore, even though this work focused on the use of centroid similarity distributions to perform a congenericity analysis of molecular sets (Figure 4, Figure 5, Figure 6 and Figure 7), comparable results would be obtained by using all pairwise similarity distributions instead.

## 4. Conclusions

Pharmacological patent applications aim at protecting the invention of chemical series of compounds acting on the same mechanism-of-action target(s) or having similar cellular phenotype(s). Therefore, by definition, the sets of molecules claimed in patents can be chemically defined by a Markush structure with only small functional variations allowed or by a looser definition of a chemical series to cover the widest possible portion within that chemical space. Both patent strategies have their strengths and limitations, and they could be balanced if a quantitative means to assess the degree of chemical compactness of all molecules contained in the patent would be available.

To this aim, a method was designed to calculate the degree of congenericity of claimed compounds in patent applications. The approach was applied and validated on a set of 750 patents from SureChEMBL for which a filtered set of molecules sharing a core chemical structure was available in SureChEMBLccs and a carefully curated set of at least two molecules was present also in ChEMBL. Patents were described by the similarity distribution around a reference compound and quantitatively characterized by its extent, mode and density values.

A principal component analysis (PCA) using the three similarity distribution descriptors successfully differentiated the patent molecular composition in each source, with filtered molecules in SureChEMBLccs showing overlapping congenericities with the manually curated sets in ChEMBL. A congenericity score (CScore), defined as the geometric mean of the extent, mode and density of similarity distributions, allowed for ranking patents according to the chemical compactness of their claimed molecules. Patent descriptors, CScores, and PC coordinates are provided as Supplementary Material to facilitate mapping future patents onto the PC space defined by the set of 750 curated patents. The current approach can be useful to describe the chemical space coverage of claimed compounds in pharmacological patent applications. More research in this direction is underway in our group.

## Data Availability

The filtered subset of molecules claimed by pharmacological US patents in SureChEMBL (SureChEMBLccs) is available for download at ftp://ftp.ebi.ac.uk/pub/databases/chembl/SureChEMBLccs (accessed on 9 July 2021). Patent descriptors (size, extent, mode and density), CScores and Principal Component coordinates (PC1 and PC2) of the 750 patents common in ChEMBL, SureChEMBLccs and SureChEMBL are provided as Supplementary Material.

## Supplementary Materials

The following are available, Table S1: Patent descriptors (size, extent, mode and density), CScores and Principal Component coordinates (PC1 and PC2) for the 750 patents common in the three patent sources used (SureChEMBL, SureChEMBLccs and ChEMBL).
