# Supplementary material for: Congenericity of Claimed Compounds in Patent Applications
Source: Molecules. 2021 Aug 30;26(17):5253. doi: 10.3390/molecules26175253 (PMC8433967; doi:10.3390/molecules26175253)
Supplement: Supplementary file 1 [file molecules-26-05253-s001.zip › molecules-1331982-supplementary.pdf]

Supplementary Material

Congenericity of Claimed Compounds in Patent Applications

Maria J. Falaguera[1,2] and Jordi Mestres[1,2,*]

[1] Research Group on Systems Pharmacology, Research Program on Biomedical Informatics (GRIB), IMIM Hospital del Mar Medical Research Institute and University Pompeu Fabra, Parc de Recerca Biomèdica (PRBB), Doctor Aiguader 88, 08003 Barcelona, Catalonia, Spain

[2] Chemotargets SL, Baldiri Reixac 4, Parc Cientific de Barcelona, 08028 Barcelona, Catalonia, Spain

* Correspondence: To whom correspondence should be addressed. Email: jmestres@imim.cat

Table S1. Patent descriptors (size, extent, mode and density), CScores and Principal Component coordinates (PC1 and PC2) for the 750 patents common in the three patent sources used (SureChEMBL, SureChEMBLccs and ChEMBL).

patent	dataset	size	extent	mode	density	cscore	PC1	PC2
US-20070135499	SureChEMBL	264	0.05	0.19	0.78	0.19	1.774	-0.902
US-20070135499	SureChEMBLccs	126	0.42	0.56	0.75	0.56	0.107	0.3
US-20070135499	ChEMBL	94	0.51	0.59	0.87	0.64	-0.658	-0.419
US-20070208166	SureChEMBL	346	0.04	0.28	0.54	0.18	2.531	0.949
US-20070208166	SureChEMBLccs	156	0.31	0.73	0.63	0.52	0.434	1.441
US-20070208166	ChEMBL	86	0.4	0.73	0.75	0.6	-0.258	0.658
US-20080096907	SureChEMBL	122	0.07	0.72	0.61	0.31	1.114	1.433
US-20080096907	SureChEMBLccs	43	0.69	0.77	0.86	0.77	-1.487	0.134
US-20080096907	ChEMBL	29	0.69	0.74	0.87	0.76	-1.454	-0.0
US-20080207655	SureChEMBLccs	24	0.7	0.71	0.86	0.75	-1.365	0.009
US-20080207655	ChEMBL	18	0.75	0.76	0.9	0.8	-1.765	-0.133
US-20080207655	SureChEMBL	120	0.04	0.14	0.69	0.16	2.276	-0.392
US-20080249081	ChEMBL	2	0.79	0.79	0.99	0.85	-2.291	-0.67
US-20080249081	SureChEMBL	369	0.15	0.28	0.76	0.32	1.395	-0.516
US-20080249081	SureChEMBLccs	67	0.64	0.72	0.85	0.73	-1.206	0.069
US-20100113462	ChEMBL	77	0.56	0.68	0.75	0.66	-0.52	0.633
US-20100113462	SureChEMBL	772	0.07	0.15	0.89	0.21	1.386	-1.739
US-20100113462	SureChEMBLccs	456	0.41	0.56	0.7	0.54	0.329	0.641
US-20100130505	ChEMBL	17	0.33	0.42	0.89	0.5	0.107	-1.019
US-20100130505	SureChEMBLccs	76	0.32	0.45	0.75	0.48	0.614	0.01
US-20100130505	SureChEMBL	319	0.05	0.14	0.81	0.18	1.776	-1.218
US-20100130737	SureChEMBLccs	667	0.47	0.62	0.72	0.59	-0.04	0.664
US-20100130737	ChEMBL	76	0.6	0.76	0.86	0.73	-1.247	0.066
US-20100130737	SureChEMBL	1631	0.04	0.14	0.82	0.17	1.76	-1.292
US-20100298347	SureChEMBLccs	120	0.4	0.51	0.76	0.54	0.237	0.112
US-20100298347	ChEMBL	39	0.54	0.72	0.85	0.69	-0.966	0.017
US-20100298347	SureChEMBL	324	0.06	0.19	0.7	0.2	2.067	-0.343
US-20110201629	ChEMBL	35	0.62	0.78	0.88	0.75	-1.423	-0.019
US-20110201629	SureChEMBL	637	0.07	0.15	0.77	0.2	1.862	-0.909
US-20110201629	SureChEMBLccs	168	0.28	0.75	0.71	0.53	0.14	0.916
US-20110257211	SureChEMBL	267	0.08	0.22	0.81	0.24	1.51	-1.029
US-20110257211	SureChEMBLccs	86	0.6	0.73	0.79	0.7	-0.896	0.485
US-20110257211	ChEMBL	34	0.68	0.86	0.85	0.79	-1.642	0.393

US-20110263566	SureChEMBL	213	0.07	0.45	0.64	0.27	1.65	0.641
US-20110263566	SureChEMBLccs	114	0.39	0.48	0.79	0.53	0.214	-0.166
US-20110263566	ChEMBL	58	0.5	0.56	0.71	0.58	0.074	0.618
US-20110263610	SureChEMBLccs	149	0.28	0.77	0.85	0.57	-0.463	-0.01
US-20110263610	ChEMBL	51	0.71	0.77	0.84	0.77	-1.456	0.283
US-20110263610	SureChEMBL	447	0.05	0.13	0.78	0.17	1.919	-1.032
US-20120115841	ChEMBL	348	0.45	0.56	0.71	0.56	0.194	0.592
US-20120115841	SureChEMBL	907	0.07	0.42	0.59	0.26	1.921	0.922
US-20120115841	SureChEMBLccs	515	0.34	0.42	0.79	0.48	0.48	-0.322
US-20120202782	ChEMBL	39	0.65	0.7	0.85	0.73	-1.181	0.031
US-20120202782	SureChEMBL	163	0.05	0.21	0.84	0.21	1.487	-1.274
US-20120202782	SureChEMBLccs	68	0.65	0.73	0.83	0.73	-1.175	0.234
US-20120225846	SureChEMBL	429	0.07	0.42	0.62	0.26	1.802	0.714
US-20120225846	SureChEMBLccs	231	0.26	0.34	0.77	0.41	0.945	-0.398
US-20120225846	ChEMBL	2	0.61	0.61	0.99	0.72	-1.422	-1.154
US-20130102619	SureChEMBLccs	69	0.35	0.63	0.72	0.54	0.224	0.623
US-20130102619	ChEMBL	35	0.73	0.86	0.87	0.82	-1.841	0.281
US-20130102619	SureChEMBL	511	0.04	0.2	0.81	0.19	1.654	-1.093
US-20140128392	SureChEMBLccs	85	0.56	0.8	0.79	0.71	-0.97	0.616
US-20140128392	ChEMBL	52	0.56	0.8	0.83	0.72	-1.129	0.339
US-20140128392	SureChEMBL	342	0.07	0.28	0.71	0.24	1.785	-0.212
US-20140155374	SureChEMBLccs	80	0.68	0.71	0.86	0.75	-1.317	-0.001
US-20140155374	SureChEMBL	132	0.04	0.2	0.88	0.19	1.377	-1.577
US-20140155374	ChEMBL	63	0.68	0.71	0.85	0.74	-1.278	0.068
US-20140309185	SureChEMBL	274	0.08	0.2	0.82	0.24	1.519	-1.141
US-20140309185	SureChEMBLccs	139	0.41	0.68	0.79	0.6	-0.319	0.278
US-20140309185	ChEMBL	96	0.41	0.68	0.8	0.61	-0.359	0.209
US-20140309237	SureChEMBL	711	0.06	0.09	0.76	0.16	2.072	-0.975
US-20140309237	SureChEMBLccs	142	0.41	0.68	0.69	0.58	0.078	0.97
US-20140309237	ChEMBL	50	0.47	0.56	0.78	0.59	-0.132	0.118
US-20150031679	SureChEMBLccs	249	0.51	0.68	0.73	0.63	-0.321	0.745
US-20150031679	ChEMBL	96	0.56	0.63	0.8	0.66	-0.597	0.178
US-20150031679	SureChEMBL	578	0.05	0.19	0.82	0.2	1.615	-1.179
US-20150094297	ChEMBL	260	0.63	0.76	0.78	0.72	-1.001	0.635
US-20150094297	SureChEMBL	1130	0.04	0.2	0.74	0.18	1.932	-0.609
US-20150094297	SureChEMBLccs	479	0.52	0.63	0.73	0.62	-0.224	0.642
US-7420055	ChEMBL	184	0.6	0.7	0.76	0.68	-0.704	0.628
US-7420055	SureChEMBL	744	0.05	0.23	0.78	0.21	1.676	-0.815
US-7420055	SureChEMBLccs	370	0.41	0.68	0.67	0.57	0.157	1.108
US-7531547	ChEMBL	41	0.65	0.85	0.89	0.79	-1.704	0.079
US-7531547	SureChEMBLccs	87	0.55	0.72	0.75	0.67	-0.593	0.714
US-7531547	SureChEMBL	194	0.04	0.04	0.49	0.09	3.312	0.775
US-7582661	SureChEMBLccs	121	0.65	0.7	0.8	0.71	-0.983	0.377
US-7582661	ChEMBL	100	0.66	0.7	0.8	0.72	-1.007	0.382
US-7582661	SureChEMBL	329	0.06	0.21	0.76	0.21	1.78	-0.715
US-7589090	SureChEMBLccs	31	0.75	0.81	0.87	0.81	-1.768	0.183
US-7589090	ChEMBL	27	0.75	0.79	0.87	0.8	-1.719	0.139
US-7589090	SureChEMBL	103	0.07	0.11	0.72	0.18	2.158	-0.649
US-7767701	ChEMBL	7	0.65	0.65	0.97	0.74	-1.536	-0.908
US-7767701	SureChEMBL	403	0.04	0.36	0.6	0.21	2.099	0.707
US-7767701	SureChEMBLccs	245	0.48	0.77	0.73	0.65	-0.467	0.925

US-7776872	SureChEMBL	129	0.05	0.28	0.64	0.21	2.11	0.262
US-7776872	SureChEMBLccs	24	0.36	0.48	0.86	0.53	0.009	-0.666
US-7776872	ChEMBL	10	0.71	0.81	0.95	0.82	-1.989	-0.392
US-7786110	SureChEMBL	269	0.04	0.06	0.52	0.11	3.144	0.611
US-7786110	SureChEMBLccs	101	0.5	0.7	0.76	0.64	-0.464	0.576
US-7786110	ChEMBL	52	0.53	0.7	0.8	0.67	-0.695	0.314
US-7799796	SureChEMBL	194	0.03	0.22	0.79	0.17	1.709	-0.916
US-7799796	ChEMBL	71	0.63	0.82	0.84	0.76	-1.385	0.35
US-7799796	SureChEMBLccs	97	0.56	0.72	0.78	0.68	-0.736	0.512
US-7879887	ChEMBL	99	0.55	0.65	0.78	0.65	-0.542	0.355
US-7879887	SureChEMBL	447	0.08	0.28	0.78	0.26	1.483	-0.691
US-7879887	SureChEMBLccs	177	0.56	0.62	0.79	0.65	-0.533	0.226
US-7910602	ChEMBL	5	0.65	0.65	0.97	0.74	-1.536	-0.908
US-7910602	SureChEMBL	144	0.11	0.36	0.76	0.31	1.296	-0.364
US-7910602	SureChEMBLccs	88	0.59	0.66	0.79	0.68	-0.702	0.328
US-8110576	SureChEMBL	392	0.06	0.15	0.77	0.19	1.886	-0.914
US-8110576	SureChEMBLccs	16	0.55	0.78	0.93	0.74	-1.453	-0.401
US-8110576	ChEMBL	7	0.65	0.65	0.94	0.74	-1.417	-0.7
US-8153641	SureChEMBLccs	115	0.51	0.74	0.75	0.66	-0.546	0.737
US-8153641	ChEMBL	53	0.56	0.8	0.78	0.7	-0.93	0.685
US-8153641	SureChEMBL	528	0.06	0.11	0.77	0.17	1.983	-1.001
US-8394808	SureChEMBLccs	72	0.43	0.53	0.71	0.54	0.314	0.517
US-8394808	ChEMBL	50	0.54	0.65	0.77	0.65	-0.479	0.419
US-8394808	SureChEMBL	157	0.04	0.11	0.85	0.16	1.714	-1.565
US-8410272	ChEMBL	63	0.53	0.57	0.84	0.63	-0.538	-0.244
US-8410272	SureChEMBL	506	0.05	0.18	0.72	0.19	2.036	-0.508
US-8410272	SureChEMBLccs	72	0.52	0.56	0.81	0.62	-0.371	-0.063
US-8426414	SureChEMBL	217	0.09	0.28	0.67	0.26	1.895	0.075
US-8426414	SureChEMBLccs	94	0.43	0.56	0.75	0.57	0.083	0.305
US-8426414	ChEMBL	4	0.76	0.76	0.97	0.82	-2.067	-0.612
US-8470800	SureChEMBL	88	0.06	0.2	0.66	0.2	2.201	-0.044
US-8470800	SureChEMBLccs	28	0.3	0.41	0.78	0.46	0.64	-0.295
US-8470800	ChEMBL	9	0.74	0.74	0.94	0.8	-1.851	-0.459
US-8470816	SureChEMBLccs	877	0.4	0.71	0.69	0.58	0.029	1.03
US-8470816	ChEMBL	7	0.86	0.91	0.95	0.91	-2.592	-0.097
US-8470816	SureChEMBL	2008	0.07	0.28	0.78	0.25	1.507	-0.696
US-8470820	ChEMBL	62	0.67	0.73	0.83	0.74	-1.223	0.245
US-8470820	SureChEMBL	290	0.07	0.19	0.66	0.21	2.202	-0.061
US-8470820	SureChEMBLccs	144	0.3	0.41	0.57	0.41	1.473	1.158
US-8470825	ChEMBL	11	0.8	0.83	0.91	0.85	-2.095	-0.025
US-8470825	SureChEMBL	170	0.04	0.14	0.82	0.17	1.76	-1.292
US-8470825	SureChEMBLccs	22	0.69	0.78	0.94	0.8	-1.829	-0.398
US-8470836	ChEMBL	22	0.68	0.71	0.9	0.76	-1.476	-0.278
US-8470836	SureChEMBLccs	136	0.43	0.57	0.83	0.59	-0.259	-0.227
US-8470836	SureChEMBL	208	0.08	0.28	0.68	0.25	1.88	0.001
US-8470837	SureChEMBLccs	100	0.57	0.72	0.77	0.68	-0.721	0.586
US-8470837	SureChEMBL	480	0.03	0.16	0.66	0.15	2.37	-0.147
US-8470837	ChEMBL	42	0.67	0.8	0.86	0.77	-1.512	0.189
US-8470841	SureChEMBLccs	89	0.6	0.73	0.75	0.69	-0.738	0.762
US-8470841	SureChEMBL	387	0.1	0.28	0.7	0.27	1.752	-0.127
US-8470841	ChEMBL	25	0.67	0.81	0.89	0.78	-1.655	0.003

US-8470843	SureChEMBLccs	14	0.73	0.73	0.92	0.79	-1.724	-0.347
US-8470843	SureChEMBL	66	0.08	0.28	0.77	0.26	1.523	-0.622
US-8470843	ChEMBL	12	0.75	0.75	0.92	0.8	-1.82	-0.293
US-8470847	SureChEMBLccs	41	0.72	0.76	0.9	0.79	-1.693	-0.149
US-8470847	SureChEMBL	113	0.07	0.16	0.65	0.19	2.314	-0.057
US-8470847	ChEMBL	13	0.75	0.8	0.94	0.83	-2.021	-0.323
US-8476255	SureChEMBLccs	271	0.47	0.77	0.66	0.62	-0.166	1.404
US-8476255	ChEMBL	58	0.47	0.68	0.84	0.65	-0.661	-0.037
US-8476255	SureChEMBL	363	0.07	0.26	0.8	0.24	1.476	-0.878
US-8476267	ChEMBL	4	0.74	0.74	0.97	0.81	-1.97	-0.666
US-8476267	SureChEMBL	242	0.04	0.21	0.66	0.18	2.225	-0.033
US-8476267	SureChEMBLccs	44	0.62	0.81	0.8	0.74	-1.178	0.6
US-8476271	ChEMBL	6	0.79	0.79	0.95	0.84	-2.132	-0.393
US-8476271	SureChEMBL	572	0.06	0.28	0.74	0.23	1.69	-0.425
US-8476271	SureChEMBLccs	56	0.28	0.75	0.87	0.57	-0.494	-0.192
US-8476295	SureChEMBL	310	0.09	0.2	0.61	0.22	2.328	0.317
US-8476295	SureChEMBLccs	111	0.39	0.62	0.72	0.56	0.152	0.622
US-8476295	ChEMBL	5	0.79	0.79	0.97	0.85	-2.212	-0.532
US-8481536	SureChEMBLccs	425	0.33	0.56	0.76	0.52	0.283	0.184
US-8481536	ChEMBL	255	0.52	0.63	0.7	0.61	-0.105	0.85
US-8481536	SureChEMBL	716	0.07	0.56	0.42	0.25	2.256	2.402
US-8481564	SureChEMBLccs	85	0.4	0.77	0.68	0.59	-0.077	1.229
US-8481564	ChEMBL	11	0.72	0.83	0.94	0.83	-2.022	-0.274
US-8481564	SureChEMBL	338	0.06	0.17	0.45	0.17	3.107	1.344
US-8481586	SureChEMBLccs	18	0.23	0.26	0.93	0.38	0.577	-1.695
US-8481586	SureChEMBL	98	0.11	0.21	0.85	0.27	1.303	-1.312
US-8481586	ChEMBL	2	0.83	0.83	0.99	0.88	-2.484	-0.563
US-8481733	SureChEMBL	1143	0.04	0.28	0.47	0.17	2.809	1.433
US-8481733	SureChEMBLccs	749	0.34	0.56	0.59	0.48	0.934	1.366
US-8481733	ChEMBL	333	0.56	0.75	0.7	0.66	-0.492	1.13
US-8481743	SureChEMBL	617	0.08	0.28	0.8	0.26	1.404	-0.83
US-8481743	SureChEMBLccs	23	0.48	0.48	0.84	0.58	-0.2	-0.465
US-8481743	ChEMBL	4	0.83	0.83	0.97	0.87	-2.405	-0.424
US-8486940	SureChEMBLccs	375	0.2	0.34	0.82	0.38	0.891	-0.776
US-8486940	ChEMBL	9	0.53	1.0	0.93	0.79	-1.939	0.065
US-8486940	SureChEMBL	1129	0.06	0.15	0.8	0.19	1.767	-1.122
US-8486953	ChEMBL	10	0.61	0.72	0.92	0.74	-1.412	-0.431
US-8486953	SureChEMBL	497	0.07	0.16	0.79	0.21	1.759	-1.026
US-8486953	SureChEMBLccs	85	0.56	0.8	0.79	0.71	-0.97	0.616
US-8486964	ChEMBL	3	0.86	0.86	0.98	0.9	-2.589	-0.413
US-8486964	SureChEMBL	176	0.06	0.23	0.74	0.22	1.811	-0.533
US-8486964	SureChEMBLccs	8	0.57	0.66	0.96	0.71	-1.329	-0.859
US-8486966	SureChEMBL	245	0.05	0.17	0.78	0.19	1.822	-0.945
US-8486966	SureChEMBLccs	38	0.47	0.56	0.79	0.59	-0.172	0.049
US-8486966	ChEMBL	22	0.65	0.75	0.9	0.76	-1.501	-0.207
US-8487111	SureChEMBLccs	39	0.41	0.56	0.83	0.58	-0.186	-0.259
US-8487111	ChEMBL	4	0.92	0.92	0.97	0.94	-2.839	-0.183
US-8487111	SureChEMBL	272	0.07	0.19	0.79	0.22	1.686	-0.961
US-8492379	SureChEMBLccs	10	0.69	0.86	0.92	0.82	-1.943	-0.086
US-8492379	ChEMBL	3	0.88	0.88	0.98	0.91	-2.686	-0.359
US-8492379	SureChEMBL	120	0.1	0.18	0.62	0.22	2.312	0.21

US-8492387	ChEMBL	7	0.48	0.48	0.94	0.6	-0.597	-1.157
US-8492387	SureChEMBL	198	0.06	0.14	0.78	0.19	1.871	-1.005
US-8492387	SureChEMBLccs	107	0.39	0.46	0.76	0.51	0.382	-0.002
US-8492392	SureChEMBL	319	0.05	0.21	0.79	0.2	1.685	-0.928
US-8492392	SureChEMBLccs	47	0.52	0.59	0.8	0.63	-0.404	0.071
US-8492392	ChEMBL	15	0.61	0.64	0.9	0.71	-1.138	-0.466
US-8492394	SureChEMBL	133	0.09	0.23	0.75	0.25	1.699	-0.587
US-8492394	SureChEMBLccs	34	0.73	0.85	0.86	0.81	-1.777	0.328
US-8492394	ChEMBL	11	0.74	0.83	0.94	0.83	-2.07	-0.264
US-8497265	SureChEMBL	1073	0.05	0.4	0.64	0.23	1.819	0.522
US-8497265	ChEMBL	320	0.46	0.69	0.72	0.61	-0.186	0.81
US-8497265	SureChEMBLccs	569	0.36	0.49	0.69	0.5	0.659	0.532
US-8497271	ChEMBL	10	0.72	0.72	0.91	0.78	-1.636	-0.305
US-8497271	SureChEMBL	201	0.08	0.28	0.57	0.23	2.316	0.762
US-8497271	SureChEMBLccs	55	0.4	0.56	0.8	0.56	-0.043	-0.057
US-8497281	ChEMBL	102	0.64	0.74	0.85	0.74	-1.255	0.112
US-8497281	SureChEMBL	1175	0.05	0.53	0.59	0.25	1.702	1.15
US-8497281	SureChEMBLccs	761	0.36	0.81	0.63	0.57	0.12	1.641
US-8497286	SureChEMBL	286	0.08	0.28	0.54	0.23	2.435	0.97
US-8497286	SureChEMBLccs	44	0.54	0.7	0.8	0.67	-0.719	0.32
US-8497286	ChEMBL	17	0.64	0.69	0.88	0.73	-1.252	-0.204
US-8497288	SureChEMBLccs	50	0.63	0.64	0.88	0.71	-1.107	-0.317
US-8497288	ChEMBL	32	0.59	0.65	0.95	0.71	-1.313	-0.801
US-8497288	SureChEMBL	137	0.06	0.57	0.67	0.28	1.264	0.688
US-8497368	SureChEMBLccs	247	0.46	0.64	0.71	0.59	-0.025	0.771
US-8497368	ChEMBL	66	0.56	0.56	0.81	0.63	-0.467	-0.042
US-8497368	SureChEMBL	681	0.06	0.19	0.8	0.21	1.67	-1.035
US-8501708	SureChEMBLccs	69	0.78	0.88	0.92	0.86	-2.208	0.004
US-8501708	SureChEMBL	115	0.05	0.86	0.76	0.32	0.227	0.688
US-8501708	ChEMBL	18	0.87	0.94	0.94	0.92	-2.649	0.042
US-8501729	SureChEMBL	461	0.07	0.21	0.81	0.23	1.558	-1.056
US-8501729	SureChEMBLccs	165	0.42	0.49	0.72	0.53	0.396	0.356
US-8501729	ChEMBL	14	0.69	0.79	0.9	0.79	-1.694	-0.099
US-8501732	SureChEMBL	743	0.03	0.28	0.73	0.18	1.801	-0.371
US-8501732	SureChEMBLccs	426	0.43	0.66	0.65	0.57	0.237	1.214
US-8501732	ChEMBL	211	0.53	0.66	0.73	0.63	-0.32	0.712
US-8501744	SureChEMBLccs	539	0.39	0.62	0.76	0.57	-0.006	0.345
US-8501744	ChEMBL	21	0.77	0.79	0.92	0.82	-1.965	-0.196
US-8501744	SureChEMBL	846	0.05	0.79	0.42	0.26	1.746	2.89
US-8501749	ChEMBL	31	0.63	0.72	0.89	0.74	-1.341	-0.213
US-8501749	SureChEMBL	169	0.07	0.43	0.66	0.27	1.619	0.459
US-8501749	SureChEMBLccs	94	0.37	0.46	0.75	0.5	0.469	0.057
US-8501750	ChEMBL	25	0.71	0.72	0.91	0.77	-1.612	-0.31
US-8501750	SureChEMBL	949	0.05	0.18	0.83	0.2	1.599	-1.269
US-8501750	SureChEMBLccs	237	0.34	0.4	0.8	0.48	0.489	-0.434
US-8501767	ChEMBL	17	0.8	0.86	0.94	0.86	-2.287	-0.167
US-8501767	SureChEMBLccs	247	0.4	0.56	0.68	0.53	0.433	0.774
US-8501767	SureChEMBL	269	0.07	0.51	0.59	0.28	1.703	1.117
US-8501783	SureChEMBLccs	66	0.37	0.66	0.68	0.55	0.262	0.975
US-8501783	ChEMBL	51	0.37	0.66	0.74	0.57	0.024	0.56
US-8501783	SureChEMBL	190	0.09	0.22	0.82	0.25	1.446	-1.093

US-8501944	SureChEMBLccs	488	0.43	0.56	0.76	0.57	0.043	0.236
US-8501944	ChEMBL	4	0.79	0.79	0.98	0.85	-2.251	-0.601
US-8501944	SureChEMBL	972	0.04	0.39	0.61	0.21	1.987	0.703
US-8501961	ChEMBL	9	0.89	0.93	0.96	0.93	-2.752	-0.107
US-8501961	SureChEMBL	201	0.08	0.28	0.62	0.24	2.118	0.416
US-8501961	SureChEMBLccs	47	0.09	0.84	0.88	0.41	-0.296	-0.165
US-8507473	SureChEMBL	474	0.07	0.09	0.82	0.17	1.809	-1.385
US-8507473	SureChEMBLccs	198	0.31	0.44	0.71	0.46	0.821	0.26
US-8507473	ChEMBL	111	0.54	0.68	0.77	0.66	-0.552	0.484
US-8507479	SureChEMBL	897	0.07	0.12	0.93	0.2	1.3	-2.081
US-8507479	SureChEMBLccs	654	0.52	0.64	0.76	0.63	-0.367	0.456
US-8507479	ChEMBL	43	0.56	0.63	0.86	0.67	-0.835	-0.237
US-8507484	SureChEMBLccs	122	0.67	0.73	0.8	0.73	-1.104	0.452
US-8507484	ChEMBL	3	0.72	0.72	0.98	0.8	-1.914	-0.789
US-8507484	SureChEMBL	438	0.05	0.28	0.38	0.17	3.142	2.061
US-8507510	ChEMBL	25	0.7	0.77	0.85	0.77	-1.471	0.208
US-8507510	SureChEMBL	169	0.05	0.12	0.61	0.15	2.618	0.123
US-8507510	SureChEMBLccs	54	0.59	0.7	0.81	0.69	-0.879	0.276
US-8507533	ChEMBL	72	0.56	0.71	0.81	0.69	-0.831	0.283
US-8507533	SureChEMBL	747	0.07	0.56	0.37	0.24	2.454	2.748
US-8507533	SureChEMBLccs	297	0.41	0.6	0.68	0.55	0.311	0.866
US-8507535	SureChEMBL	120	0.1	0.23	0.83	0.27	1.358	-1.135
US-8507535	SureChEMBLccs	51	0.7	0.73	0.86	0.76	-1.414	0.053
US-8507535	ChEMBL	24	0.71	0.72	0.83	0.75	-1.295	0.244
US-8507676	SureChEMBLccs	178	0.44	0.56	0.8	0.58	-0.139	-0.036
US-8507676	ChEMBL	50	0.62	0.72	0.82	0.72	-1.039	0.266
US-8507676	SureChEMBL	699	0.07	0.21	0.79	0.23	1.637	-0.917
US-8507683	SureChEMBLccs	20	0.61	0.81	0.87	0.75	-1.432	0.11
US-8507683	ChEMBL	7	0.83	0.91	0.95	0.9	-2.52	-0.113
US-8507683	SureChEMBL	87	0.07	0.23	0.82	0.24	1.47	-1.082
US-8524699	SureChEMBLccs	120	0.6	0.71	0.82	0.7	-0.967	0.234
US-8524699	SureChEMBL	348	0.07	0.14	0.73	0.19	2.045	-0.654
US-8524699	ChEMBL	10	0.61	0.62	0.94	0.71	-1.248	-0.786
US-8524710	SureChEMBL	293	0.06	0.18	0.8	0.21	1.694	-1.057
US-8524710	ChEMBL	80	0.53	0.62	0.79	0.64	-0.461	0.21
US-8524710	SureChEMBLccs	216	0.34	0.45	0.76	0.49	0.526	-0.049
US-8524717	SureChEMBL	415	0.06	0.22	0.66	0.21	2.153	-0.001
US-8524717	ChEMBL	16	0.48	0.68	0.89	0.66	-0.884	-0.378
US-8524717	SureChEMBLccs	191	0.37	0.56	0.87	0.56	-0.249	-0.557
US-8524722	SureChEMBL	637	0.04	0.71	0.45	0.23	1.845	2.503
US-8524722	ChEMBL	18	0.63	0.69	0.92	0.74	-1.387	-0.486
US-8524722	SureChEMBLccs	280	0.42	0.71	0.73	0.6	-0.178	0.763
US-8524917	SureChEMBL	471	0.09	0.31	0.71	0.27	1.664	-0.137
US-8524917	ChEMBL	150	0.65	0.87	0.75	0.75	-1.197	1.091
US-8524917	SureChEMBLccs	321	0.46	0.75	0.66	0.61	-0.093	1.355
US-8530460	ChEMBL	198	0.62	0.73	0.82	0.72	-1.063	0.288
US-8530460	SureChEMBLccs	254	0.55	0.7	0.79	0.67	-0.703	0.394
US-8530460	SureChEMBL	556	0.07	0.17	0.44	0.17	3.123	1.418
US-8530490	SureChEMBLccs	319	0.43	0.78	0.61	0.59	0.104	1.751
US-8530490	ChEMBL	110	0.61	0.73	0.77	0.7	-0.841	0.629
US-8530490	SureChEMBL	727	0.07	0.18	0.78	0.21	1.75	-0.913

US-8530494	SureChEMBLccs	64	0.32	0.71	0.75	0.55	-0.017	0.573
US-8530494	ChEMBL	4	0.84	0.84	0.98	0.88	-2.493	-0.467
US-8530494	SureChEMBL	499	0.08	0.28	0.8	0.26	1.404	-0.83
US-8530504	ChEMBL	78	0.57	0.76	0.81	0.71	-0.976	0.396
US-8530504	SureChEMBL	318	0.05	0.16	0.42	0.15	3.274	1.525
US-8530504	SureChEMBLccs	130	0.54	0.78	0.72	0.67	-0.596	1.047
US-8536158	SureChEMBL	172	0.08	0.28	0.7	0.25	1.8	-0.138
US-8536158	SureChEMBLccs	13	0.5	0.89	0.93	0.75	-1.6	-0.189
US-8536158	ChEMBL	6	0.83	0.89	0.97	0.89	-2.55	-0.294
US-8536165	SureChEMBL	453	0.05	0.15	0.81	0.18	1.752	-1.196
US-8536165	SureChEMBLccs	174	0.44	0.56	0.69	0.55	0.297	0.726
US-8536165	ChEMBL	48	0.62	0.62	0.8	0.67	-0.717	0.188
US-8536166	SureChEMBL	339	0.05	0.17	0.43	0.15	3.21	1.477
US-8536166	ChEMBL	2	0.83	0.83	0.99	0.88	-2.484	-0.563
US-8536166	SureChEMBLccs	53	0.71	0.72	0.85	0.76	-1.374	0.105
US-8536172	ChEMBL	55	0.69	0.77	0.88	0.78	-1.566	-0.004
US-8536172	SureChEMBL	249	0.05	0.33	0.73	0.23	1.632	-0.252
US-8536172	SureChEMBLccs	116	0.65	0.75	0.8	0.73	-1.104	0.485
US-8536175	ChEMBL	50	0.6	0.79	0.81	0.73	-1.121	0.477
US-8536175	SureChEMBL	838	0.07	0.18	0.81	0.22	1.631	-1.121
US-8536175	SureChEMBLccs	220	0.49	0.56	0.66	0.57	0.296	0.959
US-8536192	SureChEMBL	451	0.08	0.73	0.67	0.34	0.828	1.045
US-8536192	SureChEMBLccs	294	0.65	0.81	0.83	0.76	-1.369	0.408
US-8536192	ChEMBL	9	0.75	0.78	0.94	0.82	-1.973	-0.367
US-8536193	SureChEMBLccs	165	0.35	0.76	0.79	0.59	-0.369	0.42
US-8536193	ChEMBL	7	0.69	0.69	0.94	0.76	-1.61	-0.593
US-8536193	SureChEMBL	815	0.07	0.28	0.71	0.24	1.785	-0.212
US-8536198	SureChEMBLccs	206	0.7	0.81	0.82	0.77	-1.449	0.503
US-8536198	ChEMBL	23	0.75	0.86	0.88	0.83	-1.929	0.222
US-8536198	SureChEMBL	555	0.06	0.28	0.7	0.23	1.848	-0.148
US-8536227	ChEMBL	10	0.68	0.71	0.96	0.77	-1.714	-0.693
US-8536227	SureChEMBL	174	0.08	0.22	0.76	0.24	1.708	-0.683
US-8536227	SureChEMBLccs	63	0.41	0.65	0.71	0.57	0.071	0.767
US-8541380	SureChEMBL	363	0.03	0.73	0.46	0.22	1.781	2.472
US-8541380	SureChEMBLccs	165	0.36	0.71	0.67	0.56	0.204	1.147
US-8541380	ChEMBL	105	0.69	0.71	0.83	0.74	-1.222	0.212
US-8541427	SureChEMBL	251	0.07	0.2	0.8	0.22	1.622	-1.008
US-8541427	SureChEMBLccs	75	0.45	0.47	0.83	0.56	-0.064	-0.433
US-8541427	ChEMBL	43	0.69	0.76	0.87	0.77	-1.502	0.043
US-8541596	SureChEMBLccs	96	0.46	0.56	0.81	0.59	-0.227	-0.095
US-8541596	ChEMBL	11	0.75	0.86	0.91	0.84	-2.048	0.014
US-8541596	SureChEMBL	498	0.07	0.28	0.71	0.24	1.785	-0.212
US-8546370	ChEMBL	23	0.5	0.56	0.89	0.63	-0.64	-0.627
US-8546370	SureChEMBL	1865	0.05	0.28	0.8	0.22	1.476	-0.845
US-8546370	SureChEMBLccs	1477	0.33	0.56	0.79	0.53	0.164	-0.024
US-8546380	ChEMBL	34	0.6	0.69	0.87	0.71	-1.117	-0.155
US-8546380	SureChEMBL	1376	0.05	0.2	0.82	0.2	1.591	-1.157
US-8546380	SureChEMBLccs	826	0.5	0.69	0.7	0.62	-0.202	0.969
US-8546389	ChEMBL	18	0.73	0.75	0.92	0.8	-1.772	-0.304
US-8546389	SureChEMBLccs	247	0.61	0.77	0.77	0.71	-0.938	0.715
US-8546389	SureChEMBL	511	0.11	0.17	0.81	0.25	1.559	-1.122

US-8546392	SureChEMBLccs	160	0.27	0.55	0.73	0.48	0.57	0.339
US-8546392	ChEMBL	17	0.5	0.56	0.9	0.63	-0.68	-0.697
US-8546392	SureChEMBL	336	0.08	0.18	0.82	0.23	1.567	-1.185
US-8546434	SureChEMBLccs	61	0.6	0.63	0.85	0.68	-0.892	-0.147
US-8546434	ChEMBL	12	0.68	0.68	0.89	0.74	-1.364	-0.274
US-8546434	SureChEMBL	244	0.06	0.16	0.53	0.17	2.814	0.769
US-8546435	ChEMBL	3	0.8	0.8	0.98	0.86	-2.3	-0.574
US-8546435	SureChEMBL	226	0.08	0.28	0.66	0.25	1.959	0.139
US-8546435	SureChEMBLccs	24	0.76	0.85	0.9	0.83	-2.008	0.067
US-8546451	SureChEMBL	188	0.04	0.28	0.66	0.19	2.055	0.118
US-8546451	SureChEMBLccs	24	0.53	0.68	0.87	0.68	-0.924	-0.213
US-8546451	ChEMBL	10	0.62	0.62	0.91	0.7	-1.153	-0.573
US-8551963	SureChEMBL	299	0.06	0.22	0.64	0.2	2.232	0.137
US-8551963	SureChEMBLccs	19	0.79	0.79	0.91	0.83	-1.974	-0.117
US-8551963	ChEMBL	2	0.94	0.94	0.99	0.96	-3.015	-0.267
US-8551988	SureChEMBL	357	0.05	0.16	0.82	0.19	1.688	-1.244
US-8551988	ChEMBL	123	0.5	0.75	0.75	0.66	-0.546	0.753
US-8551988	SureChEMBLccs	220	0.41	0.84	0.65	0.61	-0.152	1.593
US-8552004	ChEMBL	2	0.72	0.72	0.99	0.8	-1.953	-0.858
US-8552004	SureChEMBL	212	0.07	0.28	0.82	0.25	1.348	-0.973
US-8552004	SureChEMBLccs	52	0.23	0.72	0.88	0.53	-0.341	-0.352
US-8552005	ChEMBL	13	0.74	0.79	0.92	0.81	-1.893	-0.212
US-8552005	SureChEMBL	122	0.05	0.14	0.84	0.18	1.657	-1.425
US-8552005	SureChEMBLccs	41	0.47	0.67	0.83	0.64	-0.597	0.011
US-8552017	SureChEMBL	121	0.05	0.15	0.82	0.18	1.712	-1.265
US-8552017	SureChEMBLccs	32	0.57	0.73	0.87	0.71	-1.142	-0.084
US-8552017	ChEMBL	4	0.81	0.81	0.97	0.86	-2.308	-0.478
US-8552023	SureChEMBLccs	14	0.62	0.65	0.91	0.72	-1.226	-0.508
US-8552023	ChEMBL	4	0.83	0.83	0.97	0.87	-2.405	-0.424
US-8552023	SureChEMBL	360	0.06	0.2	0.67	0.2	2.162	-0.114
US-8552033	SureChEMBLccs	15	0.6	0.77	0.87	0.74	-1.311	0.018
US-8552033	ChEMBL	2	0.56	0.56	0.99	0.68	-1.181	-1.288
US-8552033	SureChEMBL	85	0.07	0.21	0.8	0.23	1.598	-0.986
US-8552037	SureChEMBLccs	77	0.44	0.56	0.8	0.58	-0.139	-0.036
US-8552037	SureChEMBL	387	0.06	0.16	0.79	0.2	1.783	-1.031
US-8552037	ChEMBL	20	0.52	0.82	0.92	0.73	-1.438	-0.261
US-8552057	SureChEMBL	95	0.06	0.11	0.87	0.18	1.587	-1.693
US-8552057	SureChEMBLccs	12	0.37	0.43	0.92	0.53	-0.132	-1.184
US-8552057	ChEMBL	5	0.44	0.44	0.96	0.57	-0.483	-1.403
US-8552199	SureChEMBL	483	0.07	0.28	0.48	0.21	2.697	1.38
US-8552199	SureChEMBLccs	135	0.55	0.68	0.74	0.65	-0.457	0.697
US-8552199	ChEMBL	24	0.56	0.59	0.86	0.66	-0.738	-0.324
US-8557823	SureChEMBLccs	100	0.56	0.68	0.82	0.68	-0.798	0.148
US-8557823	ChEMBL	35	0.56	0.73	0.88	0.71	-1.157	-0.159
US-8557823	SureChEMBL	306	0.07	0.16	0.84	0.21	1.56	-1.372
US-8563545	ChEMBL	8	0.85	0.85	0.97	0.89	-2.501	-0.371
US-8563545	SureChEMBL	232	0.08	0.16	0.77	0.21	1.814	-0.882
US-8563545	SureChEMBLccs	15	0.67	0.85	0.93	0.81	-1.911	-0.187
US-8563553	ChEMBL	94	0.52	0.56	0.68	0.58	0.145	0.836
US-8563553	SureChEMBL	357	0.06	0.16	0.39	0.16	3.369	1.737
US-8563553	SureChEMBLccs	163	0.52	0.56	0.68	0.58	0.145	0.836

US-8563583	SureChEMBL	325	0.05	0.28	0.69	0.21	1.912	-0.084
US-8563583	SureChEMBLccs	149	0.59	0.76	0.72	0.69	-0.667	1.029
US-8563583	ChEMBL	4	0.65	0.65	0.97	0.74	-1.536	-0.908
US-8563594	SureChEMBLccs	152	0.57	0.74	0.8	0.7	-0.888	0.422
US-8563594	ChEMBL	7	0.73	0.86	0.95	0.84	-2.158	-0.273
US-8563594	SureChEMBL	262	0.09	0.5	0.65	0.31	1.441	0.69
US-8569281	SureChEMBLccs	347	0.3	0.56	0.58	0.46	1.069	1.414
US-8569281	ChEMBL	114	0.46	0.7	0.78	0.63	-0.448	0.416
US-8569281	SureChEMBL	517	0.06	0.14	0.84	0.19	1.633	-1.42
US-8569291	ChEMBL	10	0.66	0.66	0.94	0.74	-1.465	-0.673
US-8569291	SureChEMBL	352	0.1	0.28	0.73	0.27	1.633	-0.335
US-8569291	SureChEMBLccs	46	0.35	0.46	0.85	0.52	0.121	-0.645
US-8569299	SureChEMBL	384	0.05	0.19	0.83	0.2	1.575	-1.248
US-8569299	SureChEMBLccs	92	0.52	0.75	0.77	0.67	-0.673	0.625
US-8569299	ChEMBL	7	0.71	0.81	0.96	0.82	-2.029	-0.461
US-8569306	SureChEMBL	115	0.04	0.11	0.66	0.14	2.468	-0.25
US-8569306	SureChEMBLccs	26	0.68	0.71	0.91	0.76	-1.516	-0.347
US-8569306	ChEMBL	10	0.64	0.66	0.92	0.73	-1.338	-0.545
US-8569308	SureChEMBL	284	0.09	0.27	0.8	0.27	1.404	-0.846
US-8569308	ChEMBL	73	0.55	0.67	0.78	0.66	-0.591	0.398
US-8569308	SureChEMBLccs	126	0.55	0.63	0.77	0.64	-0.454	0.381
US-8569311	ChEMBL	24	0.72	0.76	0.88	0.78	-1.614	-0.01
US-8569311	SureChEMBLccs	131	0.66	0.76	0.84	0.75	-1.311	0.235
US-8569311	SureChEMBL	594	0.06	0.15	0.82	0.19	1.688	-1.26
US-8569313	ChEMBL	27	0.71	0.8	0.9	0.8	-1.766	-0.067
US-8569313	SureChEMBLccs	30	0.6	0.67	0.89	0.71	-1.147	-0.337
US-8569313	SureChEMBL	145	0.14	0.16	0.82	0.26	1.472	-1.197
US-8569314	ChEMBL	9	0.71	0.71	0.92	0.77	-1.627	-0.401
US-8569314	SureChEMBLccs	248	0.46	0.69	0.68	0.6	-0.027	1.087
US-8569314	SureChEMBL	589	0.06	0.14	0.8	0.19	1.792	-1.143
US-8569316	ChEMBL	27	0.61	0.62	0.83	0.68	-0.812	-0.025
US-8569316	SureChEMBLccs	35	0.6	0.78	0.83	0.73	-1.176	0.317
US-8569316	SureChEMBL	367	0.04	0.11	0.79	0.15	1.952	-1.15
US-8569318	ChEMBL	7	0.62	0.62	0.94	0.71	-1.272	-0.781
US-8569318	SureChEMBL	120	0.05	0.16	0.83	0.19	1.648	-1.313
US-8569318	SureChEMBLccs	30	0.55	0.65	0.82	0.66	-0.701	0.078
US-8569338	SureChEMBL	134	0.08	0.23	0.57	0.22	2.438	0.654
US-8569338	SureChEMBLccs	8	0.69	0.69	0.93	0.76	-1.571	-0.524
US-8569338	ChEMBL	2	0.87	0.87	0.99	0.91	-2.677	-0.455
US-8569511	SureChEMBL	270	0.04	0.22	0.75	0.19	1.844	-0.634
US-8569511	SureChEMBLccs	62	0.47	0.64	0.78	0.62	-0.326	0.292
US-8569511	ChEMBL	22	0.66	0.79	0.89	0.77	-1.583	-0.046
US-8575114	SureChEMBLccs	83	0.42	0.43	0.87	0.54	-0.054	-0.812
US-8575114	ChEMBL	18	0.7	0.91	0.91	0.83	-2.049	0.097
US-8575114	SureChEMBL	213	0.05	0.69	0.5	0.26	1.671	2.119
US-8575145	ChEMBL	109	0.63	0.76	0.79	0.72	-1.041	0.566
US-8575145	SureChEMBL	642	0.08	0.56	0.6	0.3	1.518	1.161
US-8575145	SureChEMBLccs	296	0.56	0.63	0.75	0.64	-0.399	0.524
US-8575157	ChEMBL	102	0.62	0.7	0.8	0.7	-0.911	0.361
US-8575157	SureChEMBL	648	0.06	0.36	0.78	0.26	1.337	-0.528
US-8575157	SureChEMBLccs	396	0.55	0.75	0.72	0.67	-0.547	0.987

US-8575186	ChEMBL	150	0.63	0.75	0.8	0.72	-1.056	0.475
US-8575186	SureChEMBLccs	320	0.43	0.74	0.7	0.61	-0.156	1.041
US-8575186	SureChEMBL	532	0.07	0.18	0.84	0.22	1.512	-1.328
US-8575197	SureChEMBLccs	216	0.5	0.68	0.7	0.62	-0.178	0.948
US-8575197	ChEMBL	9	0.56	0.65	0.93	0.7	-1.162	-0.678
US-8575197	SureChEMBL	386	0.06	0.28	0.78	0.24	1.531	-0.702
US-8575201	SureChEMBLccs	237	0.5	0.81	0.76	0.68	-0.731	0.814
US-8575201	ChEMBL	172	0.67	0.81	0.8	0.76	-1.298	0.626
US-8575201	SureChEMBL	297	0.07	0.28	0.82	0.25	1.348	-0.973
US-8575203	ChEMBL	229	0.6	0.68	0.82	0.69	-0.894	0.169
US-8575203	SureChEMBL	977	0.06	0.22	0.8	0.22	1.597	-0.97
US-8575203	SureChEMBLccs	267	0.43	0.45	0.81	0.54	0.112	-0.348
US-8575364	SureChEMBL	269	0.06	0.23	0.79	0.22	1.613	-0.879
US-8575364	SureChEMBLccs	64	0.5	0.84	0.74	0.68	-0.725	1.017
US-8575364	ChEMBL	33	0.64	0.84	0.86	0.77	-1.537	0.26
US-8575391	SureChEMBL	340	0.04	0.64	0.62	0.25	1.34	1.175
US-8575391	SureChEMBLccs	171	0.71	0.82	0.84	0.79	-1.577	0.391
US-8575391	ChEMBL	28	0.71	0.83	0.87	0.8	-1.72	0.205
US-8580778	SureChEMBLccs	28	0.59	0.68	0.83	0.69	-0.91	0.095
US-8580778	ChEMBL	8	0.62	0.62	0.93	0.71	-1.233	-0.712
US-8580778	SureChEMBL	253	0.07	0.15	0.72	0.2	2.061	-0.563
US-8580782	ChEMBL	12	0.56	0.56	0.89	0.65	-0.784	-0.596
US-8580782	SureChEMBL	4171	0.07	0.28	0.74	0.24	1.666	-0.42
US-8580782	SureChEMBLccs	2790	0.36	0.55	0.7	0.52	0.473	0.593
US-8580788	ChEMBL	7	0.78	0.79	0.95	0.84	-2.108	-0.399
US-8580788	SureChEMBL	292	0.08	0.28	0.53	0.23	2.475	1.039
US-8580788	SureChEMBLccs	101	0.54	0.75	0.8	0.69	-0.84	0.428
US-8580800	SureChEMBL	345	0.07	0.28	0.66	0.23	1.983	0.134
US-8580800	SureChEMBLccs	76	0.59	0.69	0.76	0.68	-0.656	0.601
US-8580800	ChEMBL	9	0.76	0.77	0.95	0.82	-2.012	-0.452
US-8580815	SureChEMBLccs	108	0.46	0.56	0.7	0.56	0.209	0.667
US-8580815	ChEMBL	4	0.74	0.74	0.97	0.81	-1.97	-0.666
US-8580815	SureChEMBL	285	0.14	0.23	0.72	0.29	1.698	-0.353
US-8586571	SureChEMBLccs	359	0.41	0.56	0.74	0.55	0.171	0.364
US-8586571	ChEMBL	14	0.55	0.55	0.92	0.65	-0.855	-0.831
US-8586571	SureChEMBL	710	0.06	0.28	0.39	0.19	3.078	1.997
US-8586579	SureChEMBLccs	294	0.41	0.82	0.74	0.63	-0.46	0.927
US-8586579	SureChEMBL	396	0.08	0.73	0.53	0.31	1.383	2.014
US-8586579	ChEMBL	125	0.68	0.76	0.77	0.74	-1.082	0.73
US-8586580	SureChEMBL	138	0.15	0.5	0.63	0.36	1.376	0.86
US-8586580	ChEMBL	8	0.7	0.7	0.95	0.78	-1.698	-0.635
US-8586580	SureChEMBLccs	69	0.51	0.88	0.77	0.7	-0.965	0.902
US-8586597	SureChEMBL	326	0.05	0.25	0.72	0.21	1.866	-0.357
US-8586597	ChEMBL	5	0.79	0.79	0.97	0.85	-2.212	-0.532
US-8586597	SureChEMBLccs	128	0.32	0.77	0.72	0.56	-0.044	0.911
US-8586617	SureChEMBL	800	0.15	0.24	0.77	0.3	1.452	-0.672
US-8586617	ChEMBL	140	0.31	0.56	0.71	0.5	0.529	0.52
US-8586617	SureChEMBLccs	504	0.28	0.38	0.59	0.4	1.514	0.944
US-8586620	SureChEMBL	731	0.07	0.28	0.66	0.23	1.983	0.134
US-8586620	ChEMBL	76	0.36	0.45	0.71	0.49	0.676	0.307
US-8586620	SureChEMBLccs	116	0.36	0.56	0.68	0.52	0.529	0.753

US-8592410	ChEMBL	20	0.71	0.79	0.9	0.8	-1.742	-0.089
US-8592410	SureChEMBLccs	59	0.66	0.7	0.93	0.75	-1.523	-0.518
US-8592410	SureChEMBL	245	0.06	0.37	0.73	0.25	1.511	-0.161
US-8592415	SureChEMBLccs	31	0.72	0.72	0.87	0.77	-1.477	-0.028
US-8592415	ChEMBL	30	0.76	0.8	0.92	0.82	-1.966	-0.18
US-8592415	SureChEMBL	102	0.08	0.47	0.79	0.31	0.982	-0.349
US-8592426	SureChEMBLccs	39	0.67	0.74	0.87	0.76	-1.406	-0.011
US-8592426	ChEMBL	32	0.67	0.74	0.88	0.76	-1.446	-0.08
US-8592426	SureChEMBL	180	0.06	0.09	0.59	0.15	2.746	0.202
US-8592431	ChEMBL	82	0.69	0.86	0.84	0.79	-1.626	0.467
US-8592431	SureChEMBL	1996	0.05	0.21	0.84	0.21	1.487	-1.274
US-8592431	SureChEMBLccs	1004	0.51	0.71	0.69	0.63	-0.235	1.087
US-8592432	SureChEMBL	500	0.05	0.28	0.76	0.22	1.634	-0.568
US-8592432	SureChEMBLccs	35	0.45	0.69	0.81	0.63	-0.519	0.182
US-8592432	ChEMBL	10	0.74	0.78	0.94	0.82	-1.949	-0.372
US-8592454	SureChEMBL	1202	0.07	0.28	0.8	0.25	1.428	-0.835
US-8592454	SureChEMBLccs	196	0.42	0.64	0.71	0.58	0.071	0.75
US-8592454	ChEMBL	3	0.65	0.65	0.98	0.75	-1.576	-0.977
US-8592455	SureChEMBL	409	0.05	0.24	0.83	0.22	1.454	-1.139
US-8592455	ChEMBL	114	0.57	0.78	0.75	0.69	-0.787	0.855
US-8592455	SureChEMBLccs	175	0.53	0.67	0.7	0.63	-0.226	0.941
US-8592457	ChEMBL	3	0.84	0.84	0.98	0.88	-2.493	-0.467
US-8592457	SureChEMBL	466	0.05	0.21	0.74	0.2	1.884	-0.582
US-8592457	SureChEMBLccs	25	0.56	0.67	0.83	0.68	-0.813	0.057
US-8592581	ChEMBL	12	0.71	0.76	0.92	0.79	-1.749	-0.292
US-8592581	SureChEMBL	125	0.07	0.22	0.76	0.23	1.732	-0.688
US-8592581	SureChEMBLccs	38	0.53	0.56	0.82	0.62	-0.435	-0.127
US-8598155	SureChEMBL	283	0.04	0.18	0.75	0.18	1.941	-0.721
US-8598155	SureChEMBLccs	129	0.49	0.7	0.7	0.62	-0.202	0.986
US-8598155	ChEMBL	80	0.6	0.71	0.78	0.69	-0.808	0.511
US-8598163	SureChEMBLccs	14	0.82	0.82	0.93	0.86	-2.198	-0.174
US-8598163	ChEMBL	7	0.82	0.82	0.94	0.86	-2.238	-0.244
US-8598163	SureChEMBL	163	0.07	0.33	0.72	0.26	1.624	-0.173
US-8598184	SureChEMBLccs	8	0.68	0.9	0.94	0.83	-2.096	-0.143
US-8598184	ChEMBL	5	0.81	0.81	0.98	0.86	-2.348	-0.547
US-8598184	SureChEMBL	296	0.06	0.18	0.7	0.2	2.091	-0.365
US-8598210	ChEMBL	3	0.89	0.89	0.99	0.92	-2.774	-0.402
US-8598210	SureChEMBL	130	0.1	0.34	0.77	0.3	1.329	-0.482
US-8598210	SureChEMBLccs	18	0.62	0.67	0.89	0.72	-1.195	-0.327
US-8598217	SureChEMBL	477	0.06	0.18	0.71	0.2	2.051	-0.434
US-8598217	SureChEMBLccs	174	0.33	0.53	0.58	0.47	1.07	1.365
US-8598217	ChEMBL	16	0.57	0.78	0.86	0.73	-1.223	0.093
US-8598357	SureChEMBL	244	0.07	0.12	0.89	0.2	1.459	-1.804
US-8598357	SureChEMBLccs	128	0.8	0.84	0.88	0.84	-2.0	0.205
US-8598357	ChEMBL	68	0.8	0.84	0.89	0.84	-2.04	0.135
US-8604016	SureChEMBLccs	11	0.4	0.7	0.91	0.63	-0.82	-0.514
US-8604016	ChEMBL	5	0.7	0.7	0.97	0.78	-1.777	-0.774
US-8604016	SureChEMBL	292	0.05	0.14	0.82	0.18	1.736	-1.287
US-8604046	ChEMBL	49	0.67	0.7	0.86	0.74	-1.269	-0.028
US-8604046	SureChEMBL	188	0.04	0.22	0.82	0.19	1.566	-1.119
US-8604046	SureChEMBLccs	74	0.66	0.72	0.84	0.74	-1.214	0.149

US-8604061	ChEMBL	113	0.41	0.51	0.7	0.53	0.451	0.532
US-8604061	SureChEMBL	1067	0.07	0.21	0.68	0.22	2.074	-0.156
US-8604061	SureChEMBLccs	287	0.34	0.56	0.69	0.51	0.537	0.674
US-8604206	ChEMBL	2	0.83	0.83	0.99	0.88	-2.484	-0.563
US-8604206	SureChEMBLccs	27	0.37	0.4	0.89	0.51	0.06	-1.042
US-8604206	SureChEMBL	318	0.06	0.26	0.75	0.23	1.699	-0.537
US-8604217	SureChEMBLccs	117	0.5	0.71	0.79	0.65	-0.608	0.39
US-8604217	SureChEMBL	194	0.07	0.14	0.82	0.2	1.688	-1.277
US-8604217	ChEMBL	56	0.53	0.68	0.8	0.66	-0.647	0.271
US-8609647	SureChEMBLccs	17	0.53	0.62	0.89	0.66	-0.858	-0.482
US-8609647	SureChEMBL	189	0.06	0.12	0.55	0.16	2.832	0.543
US-8609647	ChEMBL	4	0.81	0.81	0.97	0.86	-2.308	-0.478
US-8609666	SureChEMBLccs	8	0.69	0.69	0.93	0.76	-1.571	-0.524
US-8609666	SureChEMBL	289	0.07	0.14	0.55	0.18	2.759	0.592
US-8609666	ChEMBL	8	0.69	0.69	0.93	0.76	-1.571	-0.524
US-8609678	SureChEMBLccs	24	0.62	0.66	0.88	0.71	-1.131	-0.279
US-8609678	SureChEMBL	188	0.06	0.18	0.72	0.2	2.012	-0.503
US-8609678	ChEMBL	21	0.62	0.66	0.9	0.72	-1.211	-0.417
US-8609681	SureChEMBLccs	162	0.41	0.64	0.69	0.57	0.175	0.883
US-8609681	ChEMBL	21	0.69	0.7	0.86	0.75	-1.317	-0.018
US-8609681	SureChEMBL	355	0.05	0.07	0.71	0.14	2.342	-0.677
US-8609688	ChEMBL	14	0.81	0.85	0.94	0.86	-2.286	-0.184
US-8609688	SureChEMBL	409	0.06	0.15	0.81	0.19	1.728	-1.191
US-8609688	SureChEMBLccs	72	0.46	0.68	0.81	0.63	-0.518	0.165
US-8609708	ChEMBL	4	0.55	0.55	0.97	0.66	-1.054	-1.177
US-8609708	SureChEMBL	207	0.11	0.24	0.79	0.28	1.469	-0.831
US-8609708	SureChEMBLccs	4	0.55	0.55	0.97	0.66	-1.054	-1.177
US-8609852	SureChEMBL	94	0.06	0.21	0.83	0.22	1.503	-1.199
US-8609852	SureChEMBLccs	15	0.47	0.53	0.92	0.61	-0.615	-0.916
US-8609852	ChEMBL	7	0.65	0.65	0.97	0.74	-1.536	-0.908
US-8614206	SureChEMBLccs	674	0.28	0.47	0.78	0.47	0.542	-0.176
US-8614206	ChEMBL	198	0.64	0.8	0.79	0.74	-1.162	0.658
US-8614206	SureChEMBL	1545	0.05	0.48	0.48	0.23	2.26	1.803
US-8614213	SureChEMBLccs	706	0.36	0.56	0.78	0.54	0.132	0.061
US-8614213	ChEMBL	72	0.59	0.77	0.82	0.72	-1.088	0.359
US-8614213	SureChEMBL	1168	0.06	0.23	0.85	0.23	1.375	-1.294
US-8614215	SureChEMBLccs	141	0.33	0.59	0.79	0.54	0.091	0.041
US-8614215	SureChEMBL	385	0.05	0.28	0.45	0.18	2.864	1.577
US-8614215	ChEMBL	83	0.6	0.72	0.77	0.69	-0.793	0.602
US-8614220	SureChEMBL	208	0.06	0.17	0.79	0.2	1.758	-1.009
US-8614220	SureChEMBLccs	60	0.62	0.75	0.83	0.73	-1.151	0.262
US-8614220	ChEMBL	10	0.7	0.91	0.94	0.84	-2.168	-0.111
US-8614234	SureChEMBL	225	0.07	0.18	0.83	0.22	1.551	-1.259
US-8614234	SureChEMBLccs	108	0.66	0.73	0.8	0.73	-1.08	0.447
US-8614234	ChEMBL	91	0.66	0.78	0.81	0.75	-1.241	0.486
US-8618098	SureChEMBLccs	6	0.62	0.62	0.95	0.71	-1.312	-0.85
US-8618098	ChEMBL	3	0.59	0.59	0.98	0.7	-1.286	-1.138
US-8618098	SureChEMBL	237	0.04	0.2	0.6	0.17	2.487	0.36
US-8618103	ChEMBL	119	0.6	0.77	0.75	0.7	-0.835	0.849
US-8618103	SureChEMBL	403	0.05	0.56	0.58	0.25	1.669	1.284
US-8618103	SureChEMBLccs	242	0.43	0.65	0.77	0.6	-0.215	0.362

US-8618107	ChEMBL	24	0.57	0.61	0.84	0.66	-0.731	-0.137
US-8618107	SureChEMBL	962	0.05	0.17	0.81	0.19	1.703	-1.153
US-8618107	SureChEMBLccs	182	0.17	0.28	0.9	0.35	0.791	-1.475
US-8618158	SureChEMBL	206	0.07	0.22	0.8	0.23	1.573	-0.965
US-8618158	SureChEMBLccs	101	0.6	0.73	0.76	0.69	-0.777	0.693
US-8618158	ChEMBL	59	0.64	0.78	0.87	0.76	-1.431	0.061
US-8618286	SureChEMBLccs	178	0.34	0.56	0.65	0.5	0.696	0.95
US-8618286	ChEMBL	32	0.62	0.71	0.82	0.71	-1.015	0.245
US-8618286	SureChEMBL	779	0.08	0.26	0.67	0.24	1.968	0.027
US-8618303	SureChEMBLccs	51	0.54	0.6	0.81	0.64	-0.516	0.034
US-8618303	ChEMBL	33	0.67	0.68	0.84	0.73	-1.141	0.067
US-8618303	SureChEMBL	92	0.09	0.2	0.88	0.25	1.257	-1.551
US-8623863	ChEMBL	12	0.71	0.71	0.94	0.78	-1.707	-0.539
US-8623863	SureChEMBL	484	0.05	0.21	0.74	0.2	1.884	-0.582
US-8623863	SureChEMBLccs	26	0.48	0.56	0.88	0.62	-0.553	-0.569
US-8623865	SureChEMBL	57	0.08	0.08	0.78	0.17	1.968	-1.125
US-8623865	SureChEMBLccs	5	0.87	0.87	0.96	0.9	-2.558	-0.248
US-8623865	ChEMBL	2	0.86	0.86	0.99	0.9	-2.629	-0.482
US-8623883	SureChEMBL	379	0.05	0.17	0.74	0.18	1.981	-0.668
US-8623883	SureChEMBLccs	28	0.49	0.64	0.86	0.65	-0.692	-0.252
US-8623883	ChEMBL	7	0.84	0.9	0.95	0.9	-2.519	-0.129
US-8623885	SureChEMBL	559	0.04	0.16	0.79	0.17	1.831	-1.041
US-8623885	ChEMBL	31	0.77	0.77	0.89	0.81	-1.798	-0.032
US-8623885	SureChEMBLccs	177	0.48	0.71	0.75	0.63	-0.401	0.656
US-8623889	ChEMBL	357	0.52	0.72	0.73	0.65	-0.442	0.837
US-8623889	SureChEMBL	1517	0.05	0.21	0.79	0.2	1.685	-0.928
US-8623889	SureChEMBLccs	519	0.5	0.75	0.69	0.64	-0.308	1.168
US-8623892	ChEMBL	4	0.71	0.71	0.97	0.79	-1.826	-0.747
US-8623892	SureChEMBL	237	0.05	0.28	0.7	0.21	1.872	-0.153
US-8623892	SureChEMBLccs	27	0.54	0.74	0.85	0.7	-1.015	0.06
US-8623901	SureChEMBL	604	0.06	0.2	0.64	0.2	2.281	0.094
US-8623901	SureChEMBLccs	171	0.64	0.79	0.8	0.74	-1.177	0.567
US-8623901	ChEMBL	43	0.74	0.83	0.88	0.81	-1.832	0.152
US-8623903	SureChEMBLccs	104	0.19	0.44	0.77	0.4	0.871	-0.218
US-8623903	ChEMBL	17	0.73	0.8	0.9	0.81	-1.814	-0.057
US-8623903	SureChEMBL	498	0.07	0.21	0.81	0.23	1.558	-1.056
US-8623906	SureChEMBLccs	68	0.74	0.78	0.84	0.79	-1.552	0.32
US-8623906	ChEMBL	66	0.77	0.8	0.88	0.82	-1.831	0.102
US-8623906	SureChEMBL	147	0.11	0.21	0.81	0.27	1.462	-1.035
US-8629135	SureChEMBLccs	10	0.75	0.76	0.94	0.81	-1.924	-0.41
US-8629135	SureChEMBL	95	0.06	0.28	0.82	0.24	1.372	-0.978
US-8629135	ChEMBL	4	0.76	0.76	0.97	0.82	-2.067	-0.612
US-8629141	SureChEMBL	675	0.06	0.16	0.77	0.19	1.862	-0.892
US-8629141	SureChEMBLccs	90	0.28	0.74	0.86	0.56	-0.43	-0.144
US-8629141	ChEMBL	26	0.7	0.84	0.89	0.81	-1.8	0.083
US-8629158	SureChEMBL	383	0.05	0.18	0.82	0.19	1.639	-1.2
US-8629158	SureChEMBLccs	167	0.43	0.59	0.64	0.55	0.446	1.131
US-8629158	ChEMBL	38	0.56	0.66	0.8	0.67	-0.67	0.243
US-8629167	SureChEMBLccs	277	0.38	0.56	0.77	0.55	0.124	0.141
US-8629167	ChEMBL	23	0.63	0.8	0.9	0.77	-1.574	-0.109
US-8629167	SureChEMBL	515	0.05	0.28	0.73	0.22	1.753	-0.361

US-8633183	ChEMBL	570	0.62	0.8	0.79	0.73	-1.114	0.647
US-8633183	SureChEMBL	844	0.06	0.69	0.55	0.28	1.449	1.778
US-8633183	SureChEMBLccs	615	0.61	0.72	0.78	0.7	-0.856	0.538
US-8633188	ChEMBL	11	0.65	0.72	0.92	0.76	-1.508	-0.41
US-8633188	SureChEMBL	596	0.06	0.24	0.76	0.22	1.708	-0.65
US-8633188	SureChEMBLccs	262	0.61	0.74	0.79	0.71	-0.945	0.512
US-8633196	ChEMBL	13	0.7	0.76	0.92	0.79	-1.725	-0.298
US-8633196	SureChEMBLccs	145	0.35	0.44	0.74	0.48	0.606	0.073
US-8633196	SureChEMBL	278	0.08	0.15	0.85	0.22	1.521	-1.457
US-8633206	SureChEMBLccs	334	0.5	0.74	0.82	0.67	-0.8	0.247
US-8633206	ChEMBL	136	0.73	0.8	0.85	0.79	-1.616	0.289
US-8633206	SureChEMBL	530	0.06	0.81	0.58	0.3	1.038	1.831
US-8633208	SureChEMBLccs	35	0.55	0.56	0.84	0.64	-0.562	-0.255
US-8633208	ChEMBL	5	0.56	0.56	0.96	0.67	-1.062	-1.081
US-8633208	SureChEMBL	218	0.1	0.24	0.75	0.26	1.651	-0.56
US-8633212	ChEMBL	88	0.5	0.66	0.74	0.63	-0.288	0.627
US-8633212	SureChEMBL	2043	0.07	0.56	0.44	0.26	2.176	2.263
US-8633212	SureChEMBLccs	1540	0.28	0.38	0.76	0.43	0.84	-0.232
US-8633226	SureChEMBL	246	0.07	0.23	0.76	0.23	1.708	-0.666
US-8633226	SureChEMBLccs	17	0.72	0.81	0.91	0.81	-1.854	-0.11
US-8633226	ChEMBL	5	0.81	0.88	0.97	0.88	-2.478	-0.326
US-8633231	SureChEMBL	284	0.07	0.33	0.68	0.25	1.782	0.104
US-8633231	SureChEMBLccs	31	0.54	0.76	0.8	0.69	-0.865	0.45
US-8633231	ChEMBL	18	0.56	0.62	0.87	0.67	-0.851	-0.328
US-8637500	SureChEMBLccs	557	0.51	0.67	0.72	0.63	-0.257	0.793
US-8637500	ChEMBL	339	0.56	0.65	0.77	0.65	-0.527	0.429
US-8637500	SureChEMBL	1236	0.04	0.16	0.78	0.17	1.87	-0.972
US-8637501	ChEMBL	110	0.54	0.75	0.72	0.66	-0.523	0.982
US-8637501	SureChEMBL	688	0.05	0.15	0.87	0.19	1.514	-1.611
US-8637501	SureChEMBLccs	383	0.37	0.44	0.86	0.52	0.082	-0.747
US-8637502	ChEMBL	31	0.5	0.5	0.83	0.59	-0.257	-0.342
US-8637502	SureChEMBL	731	0.06	0.28	0.75	0.23	1.65	-0.494
US-8637502	SureChEMBLccs	128	0.37	0.56	0.78	0.54	0.108	0.066
US-8637504	SureChEMBL	408	0.06	0.16	0.79	0.2	1.783	-1.031
US-8637504	SureChEMBLccs	171	0.28	0.56	0.67	0.47	0.76	0.781
US-8637504	ChEMBL	34	0.48	0.78	0.81	0.67	-0.809	0.393
US-8637507	SureChEMBLccs	155	0.56	0.71	0.72	0.66	-0.474	0.905
US-8637507	ChEMBL	12	0.65	0.73	0.93	0.76	-1.572	-0.458
US-8637507	SureChEMBL	372	0.04	0.56	0.44	0.21	2.248	2.248
US-8637526	SureChEMBLccs	618	0.46	0.56	0.64	0.55	0.447	1.082
US-8637526	ChEMBL	4	0.51	0.51	0.97	0.63	-0.86	-1.284
US-8637526	SureChEMBL	1494	0.05	0.15	0.81	0.18	1.752	-1.196
US-8637532	SureChEMBLccs	1041	0.4	0.56	0.72	0.54	0.274	0.497
US-8637532	SureChEMBL	1297	0.07	0.28	0.71	0.24	1.785	-0.212
US-8637532	ChEMBL	659	0.43	0.6	0.73	0.57	0.065	0.53
US-8637558	SureChEMBL	534	0.08	0.34	0.54	0.24	2.29	1.1
US-8637558	SureChEMBLccs	402	0.27	0.56	0.67	0.47	0.784	0.776
US-8637558	ChEMBL	121	0.5	0.71	0.76	0.65	-0.489	0.597
US-8648092	SureChEMBL	574	0.08	0.14	0.86	0.21	1.505	-1.548
US-8648092	SureChEMBLccs	259	0.51	0.56	0.69	0.58	0.129	0.762
US-8648092	ChEMBL	229	0.51	0.56	0.7	0.58	0.089	0.693

US-8653087	SureChEMBLccs	632	0.53	0.65	0.72	0.63	-0.256	0.76
US-8653087	ChEMBL	562	0.54	0.69	0.72	0.64	-0.378	0.852
US-8653087	SureChEMBL	1373	0.05	0.25	0.8	0.22	1.549	-0.91
US-8653091	ChEMBL	46	0.71	0.82	0.88	0.8	-1.736	0.114
US-8653091	SureChEMBL	301	0.06	0.28	0.82	0.24	1.372	-0.978
US-8653091	SureChEMBLccs	80	0.7	0.92	0.81	0.8	-1.677	0.81
US-8653092	ChEMBL	90	0.61	0.86	0.85	0.76	-1.474	0.357
US-8653092	SureChEMBL	578	0.06	0.71	0.52	0.28	1.519	2.029
US-8653092	SureChEMBLccs	234	0.62	0.75	0.79	0.72	-0.993	0.539
US-8653100	SureChEMBL	657	0.08	0.26	0.84	0.26	1.294	-1.15
US-8653100	SureChEMBLccs	396	0.45	0.75	0.68	0.61	-0.148	1.212
US-8653100	ChEMBL	173	0.54	0.73	0.79	0.68	-0.752	0.454
US-8653111	SureChEMBLccs	209	0.19	0.47	0.64	0.39	1.314	0.747
US-8653111	ChEMBL	18	0.51	0.56	0.91	0.64	-0.744	-0.761
US-8653111	SureChEMBL	531	0.07	0.18	0.82	0.22	1.591	-1.19
US-8653125	SureChEMBLccs	66	0.48	0.56	0.87	0.62	-0.513	-0.499
US-8653125	ChEMBL	19	0.48	0.62	0.86	0.63	-0.619	-0.3
US-8653125	SureChEMBL	250	0.03	0.28	0.53	0.16	2.595	1.013
US-8653257	ChEMBL	2	0.86	0.86	0.99	0.9	-2.629	-0.482
US-8653257	SureChEMBL	275	0.07	0.32	0.71	0.25	1.688	-0.125
US-8653257	SureChEMBLccs	9	0.68	0.81	0.96	0.81	-1.957	-0.476
US-8653263	SureChEMBL	1011	0.07	0.37	0.62	0.25	1.923	0.606
US-8653263	SureChEMBLccs	496	0.38	0.6	0.61	0.52	0.661	1.335
US-8653263	ChEMBL	369	0.56	0.8	0.75	0.7	-0.811	0.893
US-8653304	SureChEMBL	295	0.06	0.36	0.67	0.24	1.773	0.233
US-8653304	SureChEMBLccs	145	0.46	0.65	0.85	0.63	-0.604	-0.176
US-8653304	ChEMBL	9	0.7	0.7	0.92	0.77	-1.579	-0.428
US-8664219	SureChEMBL	124	0.06	0.1	0.88	0.17	1.571	-1.784
US-8664219	ChEMBL	12	0.57	0.89	0.9	0.77	-1.649	0.055
US-8664219	SureChEMBLccs	26	0.51	0.75	0.83	0.68	-0.887	0.205
US-8664230	ChEMBL	6	0.63	0.63	0.95	0.72	-1.36	-0.823
US-8664230	SureChEMBL	142	0.09	0.17	0.65	0.22	2.242	-0.025
US-8664230	SureChEMBLccs	33	0.59	0.67	0.85	0.7	-0.965	-0.065
US-8664236	ChEMBL	34	0.73	0.79	0.88	0.8	-1.711	0.06
US-8664236	SureChEMBL	357	0.06	0.17	0.77	0.2	1.838	-0.871
US-8664236	SureChEMBLccs	46	0.7	0.78	0.88	0.78	-1.615	0.023
US-8664258	SureChEMBL	763	0.07	0.2	0.81	0.22	1.582	-1.077
US-8664258	SureChEMBLccs	364	0.46	0.7	0.66	0.6	0.028	1.247
US-8664258	ChEMBL	3	0.64	0.64	0.98	0.74	-1.528	-1.004
US-8669252	SureChEMBLccs	74	0.46	0.83	0.74	0.66	-0.605	0.975
US-8669252	ChEMBL	11	0.78	0.78	0.9	0.82	-1.886	-0.074
US-8669252	SureChEMBL	389	0.05	0.19	0.8	0.2	1.694	-1.04
US-8669380	SureChEMBLccs	28	0.63	0.65	0.9	0.72	-1.21	-0.434
US-8669380	ChEMBL	2	0.9	0.9	0.99	0.93	-2.822	-0.375
US-8669380	SureChEMBL	223	0.07	0.19	0.66	0.21	2.202	-0.061
US-8673905	ChEMBL	41	0.56	0.57	0.88	0.65	-0.769	-0.505
US-8673905	SureChEMBL	241	0.09	0.12	0.66	0.19	2.323	-0.202
US-8673905	SureChEMBLccs	119	0.5	0.55	0.8	0.6	-0.259	-0.026
US-8673906	SureChEMBL	404	0.05	0.74	0.43	0.25	1.827	2.712
US-8673906	SureChEMBLccs	208	0.5	0.56	0.83	0.61	-0.402	-0.212
US-8673906	ChEMBL	90	0.68	0.83	0.8	0.77	-1.371	0.674

US-8673938	SureChEMBL	652	0.05	0.56	0.44	0.23	2.224	2.253
US-8673938	SureChEMBLccs	373	0.41	0.57	0.6	0.52	0.702	1.354
US-8673938	ChEMBL	19	0.54	0.57	0.9	0.65	-0.8	-0.654
US-8673950	SureChEMBLccs	151	0.48	0.78	0.71	0.64	-0.412	1.085
US-8673950	ChEMBL	61	0.67	0.85	0.85	0.79	-1.593	0.366
US-8673950	SureChEMBL	327	0.15	0.28	0.83	0.33	1.117	-1.001
US-8673966	ChEMBL	38	0.75	0.8	0.88	0.81	-1.783	0.092
US-8673966	SureChEMBL	165	0.06	0.16	0.75	0.19	1.941	-0.754
US-8673966	SureChEMBLccs	48	0.73	0.79	0.87	0.79	-1.671	0.129
US-8674100	ChEMBL	81	0.53	0.71	0.77	0.66	-0.6	0.544
US-8674100	SureChEMBL	608	0.09	0.28	0.48	0.23	2.649	1.39
US-8674100	SureChEMBLccs	339	0.35	0.54	0.63	0.49	0.799	1.051
US-8680116	ChEMBL	10	0.51	0.56	0.92	0.64	-0.783	-0.83
US-8680116	SureChEMBLccs	102	0.5	0.56	0.79	0.6	-0.244	0.065
US-8680116	SureChEMBL	419	0.05	0.19	0.77	0.19	1.813	-0.833
US-8680120	SureChEMBLccs	263	0.35	0.56	0.68	0.51	0.553	0.748
US-8680120	ChEMBL	31	0.56	0.61	0.83	0.66	-0.668	-0.073
US-8680120	SureChEMBL	658	0.07	0.28	0.79	0.25	1.467	-0.766
US-8680132	SureChEMBLccs	145	0.46	0.82	0.78	0.67	-0.739	0.676
US-8680132	ChEMBL	21	0.74	0.84	0.89	0.82	-1.896	0.104
US-8680132	SureChEMBL	680	0.06	0.14	0.59	0.17	2.625	0.31
US-8691827	ChEMBL	9	0.54	0.76	0.93	0.73	-1.38	-0.45
US-8691827	SureChEMBL	23	0.18	0.2	0.83	0.31	1.239	-1.159
US-8691827	SureChEMBLccs	13	0.61	0.61	0.92	0.7	-1.145	-0.669
US-8691852	SureChEMBL	241	0.07	0.14	0.83	0.2	1.649	-1.346
US-8691852	SureChEMBLccs	82	0.56	0.73	0.76	0.68	-0.681	0.672
US-8691852	ChEMBL	26	0.57	0.59	0.81	0.65	-0.564	0.028
US-8691856	SureChEMBL	26	0.14	0.7	0.83	0.43	0.122	-0.096
US-8691856	SureChEMBLccs	22	0.71	0.8	0.88	0.79	-1.687	0.071
US-8691856	ChEMBL	2	0.87	0.87	0.99	0.91	-2.677	-0.455
US-8691986	SureChEMBL	50	0.2	0.28	0.84	0.36	0.957	-1.044
US-8691986	ChEMBL	17	0.75	0.8	0.92	0.82	-1.942	-0.185
US-8691986	SureChEMBLccs	27	0.62	0.8	0.9	0.76	-1.55	-0.114
US-8691993	ChEMBL	13	0.8	0.83	0.95	0.86	-2.254	-0.302
US-8691993	SureChEMBL	44	0.18	0.6	0.8	0.44	0.387	-0.084
US-8691993	SureChEMBLccs	34	0.7	0.73	0.87	0.76	-1.454	-0.017
US-8697708	ChEMBL	11	0.59	0.59	0.94	0.69	-1.128	-0.862
US-8697708	SureChEMBL	1033	0.05	0.09	0.75	0.15	2.135	-0.911
US-8697708	SureChEMBLccs	370	0.42	0.71	0.7	0.59	-0.059	0.971
US-8697715	SureChEMBL	935	0.05	0.24	0.82	0.21	1.494	-1.07
US-8697715	SureChEMBLccs	514	0.52	0.64	0.78	0.64	-0.446	0.318
US-8697715	ChEMBL	41	0.59	0.88	0.85	0.76	-1.474	0.39
US-8697868	SureChEMBLccs	321	0.66	0.81	0.78	0.75	-1.195	0.759
US-8697868	ChEMBL	7	0.77	0.77	0.94	0.82	-1.996	-0.378
US-8697868	SureChEMBL	504	0.06	0.21	0.8	0.22	1.622	-0.992
US-8697869	SureChEMBLccs	57	0.73	0.79	0.86	0.79	-1.631	0.198
US-8697869	ChEMBL	36	0.73	0.79	0.86	0.79	-1.631	0.198
US-8697869	SureChEMBL	153	0.06	0.7	0.63	0.3	1.107	1.246
US-8697911	SureChEMBLccs	21	0.34	0.8	0.89	0.62	-0.839	-0.191
US-8697911	SureChEMBL	91	0.1	0.28	0.76	0.28	1.514	-0.542
US-8697911	ChEMBL	3	0.79	0.79	0.98	0.85	-2.251	-0.601

US-8703767	SureChEMBL	169	0.05	0.21	0.76	0.2	1.804	-0.72
US-8703767	SureChEMBLccs	12	0.64	0.7	0.92	0.74	-1.435	-0.459
US-8703767	ChEMBL	7	0.76	0.76	0.95	0.82	-1.988	-0.474
US-8703768	SureChEMBL	1205	0.07	0.23	0.45	0.19	2.938	1.479
US-8703768	SureChEMBLccs	697	0.23	0.45	0.69	0.41	1.068	0.378
US-8703768	ChEMBL	422	0.56	0.75	0.72	0.67	-0.571	0.992
US-8703770	SureChEMBLccs	28	0.46	0.7	0.83	0.64	-0.646	0.07
US-8703770	ChEMBL	2	0.73	0.73	0.99	0.81	-2.002	-0.831
US-8703770	SureChEMBL	459	0.06	0.12	0.81	0.18	1.8	-1.256
US-8703771	ChEMBL	11	0.88	0.94	0.95	0.92	-2.712	-0.022
US-8703771	SureChEMBL	119	0.07	0.15	0.68	0.19	2.219	-0.286
US-8703771	SureChEMBLccs	27	0.49	0.86	0.89	0.72	-1.344	0.017
US-8703807	ChEMBL	9	0.76	0.8	0.94	0.83	-2.045	-0.318
US-8703807	SureChEMBL	115	0.06	0.59	0.65	0.28	1.295	0.87
US-8703807	SureChEMBLccs	49	0.55	0.68	0.88	0.69	-1.012	-0.272
US-8703811	SureChEMBL	245	0.05	0.17	0.65	0.18	2.338	-0.045
US-8703811	SureChEMBLccs	84	0.54	0.64	0.78	0.65	-0.494	0.328
US-8703811	ChEMBL	70	0.54	0.7	0.78	0.67	-0.64	0.458
US-8710076	SureChEMBLccs	46	0.71	0.82	0.87	0.8	-1.696	0.184
US-8710076	ChEMBL	4	0.78	0.78	0.97	0.84	-2.164	-0.559
US-8710076	SureChEMBL	521	0.06	0.13	0.81	0.18	1.776	-1.234
US-8716285	SureChEMBLccs	98	0.43	0.47	0.8	0.54	0.103	-0.236
US-8716285	ChEMBL	42	0.68	0.85	0.86	0.79	-1.657	0.302
US-8716285	SureChEMBL	280	0.12	0.19	0.83	0.27	1.407	-1.211
US-8716287	ChEMBL	65	0.66	0.79	0.82	0.75	-1.305	0.439
US-8716287	SureChEMBL	418	0.07	0.2	0.79	0.22	1.662	-0.939
US-8716287	SureChEMBLccs	124	0.36	0.65	0.7	0.55	0.231	0.81
US-8722683	SureChEMBL	257	0.07	0.79	0.63	0.33	0.865	1.447
US-8722683	SureChEMBLccs	140	0.75	0.89	0.87	0.83	-1.962	0.356
US-8722683	ChEMBL	77	0.77	0.84	0.88	0.83	-1.928	0.189
US-8722692	SureChEMBL	1142	0.04	0.25	0.77	0.2	1.692	-0.708
US-8722692	SureChEMBLccs	774	0.5	0.56	0.73	0.59	-0.006	0.48
US-8722692	ChEMBL	715	0.51	0.56	0.73	0.59	-0.03	0.485
US-8722709	SureChEMBL	206	0.07	0.19	0.81	0.22	1.607	-1.099
US-8722709	ChEMBL	45	0.54	0.55	0.83	0.63	-0.474	-0.213
US-8722709	SureChEMBLccs	71	0.38	0.56	0.72	0.54	0.322	0.487
US-8722718	ChEMBL	2	0.84	0.84	0.99	0.89	-2.532	-0.536
US-8722718	SureChEMBLccs	12	0.35	0.36	0.9	0.48	0.165	-1.208
US-8722718	SureChEMBL	114	0.07	0.14	0.75	0.19	1.966	-0.792
US-8722721	ChEMBL	16	0.76	0.81	0.91	0.82	-1.95	-0.089
US-8722721	SureChEMBLccs	38	0.71	0.78	0.88	0.79	-1.639	0.028
US-8722721	SureChEMBL	103	0.04	0.18	0.65	0.17	2.337	-0.029
US-8722890	ChEMBL	5	0.81	0.86	0.97	0.88	-2.43	-0.37
US-8722890	SureChEMBLccs	10	0.74	0.87	0.92	0.84	-2.088	-0.038
US-8722890	SureChEMBL	374	0.06	0.15	0.79	0.19	1.807	-1.052
US-8722895	ChEMBL	7	0.76	0.76	0.96	0.82	-2.027	-0.543
US-8722895	SureChEMBLccs	24	0.44	0.71	0.86	0.65	-0.741	-0.126
US-8722895	SureChEMBL	149	0.1	0.51	0.64	0.32	1.432	0.786
US-8729061	SureChEMBLccs	52	0.21	0.48	0.8	0.43	0.607	-0.329
US-8729061	SureChEMBL	288	0.08	0.56	0.61	0.3	1.478	1.092
US-8729061	ChEMBL	3	0.63	0.63	0.99	0.73	-1.519	-1.1

US-8729074	SureChEMBL	310	0.05	0.15	0.79	0.18	1.831	-1.058
US-8729074	SureChEMBLccs	26	0.6	0.78	0.85	0.74	-1.256	0.178
US-8729074	ChEMBL	18	0.65	0.78	0.89	0.77	-1.534	-0.073
US-8729078	SureChEMBL	351	0.04	0.16	0.82	0.17	1.712	-1.249
US-8729078	SureChEMBLccs	102	0.51	0.72	0.75	0.65	-0.497	0.693
US-8729078	ChEMBL	44	0.62	0.66	0.84	0.7	-0.973	-0.002
US-8729113	SureChEMBLccs	193	0.33	0.56	0.81	0.53	0.085	-0.162
US-8729113	ChEMBL	60	0.51	0.56	0.86	0.63	-0.545	-0.415
US-8729113	SureChEMBL	699	0.09	0.28	0.7	0.26	1.776	-0.132
US-8729273	ChEMBL	26	0.54	0.82	0.85	0.72	-1.209	0.234
US-8729273	SureChEMBL	174	0.09	0.22	0.87	0.26	1.248	-1.439
US-8729273	SureChEMBLccs	82	0.46	0.51	0.73	0.56	0.212	0.351
US-8735395	ChEMBL	5	0.86	0.86	0.98	0.9	-2.589	-0.413
US-8735395	SureChEMBL	316	0.06	0.62	0.64	0.29	1.261	1.004
US-8735395	SureChEMBLccs	48	0.66	0.82	0.92	0.79	-1.774	-0.188
US-8735593	ChEMBL	5	0.78	0.85	0.97	0.86	-2.333	-0.407
US-8735593	SureChEMBLccs	7	0.64	0.85	0.95	0.8	-1.918	-0.341
US-8735593	SureChEMBL	104	0.04	0.1	0.86	0.15	1.699	-1.656
US-8741907	SureChEMBLccs	23	0.54	0.62	0.84	0.66	-0.684	-0.131
US-8741907	ChEMBL	7	0.62	0.62	0.94	0.71	-1.272	-0.781
US-8741907	SureChEMBL	87	0.07	0.12	0.7	0.18	2.213	-0.489
US-8741923	SureChEMBLccs	57	0.46	0.52	0.76	0.57	0.068	0.165
US-8741923	ChEMBL	16	0.53	0.65	0.88	0.67	-0.891	-0.348
US-8741923	SureChEMBL	490	0.05	0.11	0.63	0.15	2.563	-0.037
US-8741944	ChEMBL	10	0.78	0.86	0.96	0.86	-2.318	-0.316
US-8741944	SureChEMBL	129	0.07	0.28	0.8	0.25	1.428	-0.835
US-8741944	SureChEMBLccs	32	0.18	0.75	0.85	0.49	-0.175	-0.105
US-8742110	SureChEMBL	1176	0.05	0.17	0.84	0.19	1.584	-1.36
US-8742110	SureChEMBLccs	663	0.53	0.61	0.81	0.64	-0.516	0.05
US-8742110	ChEMBL	161	0.63	0.72	0.84	0.72	-1.142	0.133
US-8742113	SureChEMBL	1010	0.07	0.28	0.83	0.25	1.309	-1.042
US-8742113	SureChEMBLccs	878	0.31	0.56	0.76	0.51	0.331	0.174
US-8742113	ChEMBL	7	0.77	0.77	0.96	0.83	-2.076	-0.516
US-8742115	SureChEMBLccs	36	0.75	0.86	0.87	0.82	-1.889	0.291
US-8742115	ChEMBL	34	0.78	0.79	0.89	0.82	-1.87	0.017
US-8742115	SureChEMBL	134	0.06	0.4	0.67	0.25	1.676	0.32
US-8742134	ChEMBL	3	1.0	1.0	0.99	1.0	-3.305	-0.106
US-8742134	SureChEMBL	159	0.07	0.23	0.82	0.24	1.47	-1.082
US-8742134	SureChEMBLccs	38	0.83	0.83	0.99	0.88	-2.484	-0.563
US-8742138	ChEMBL	34	0.69	0.75	0.83	0.75	-1.319	0.298
US-8742138	SureChEMBL	178	0.19	0.26	0.82	0.34	1.109	-0.954
US-8742138	SureChEMBLccs	123	0.56	0.71	0.76	0.67	-0.633	0.629
US-8748418	SureChEMBL	342	0.07	0.25	0.66	0.23	2.056	0.069
US-8748418	SureChEMBLccs	156	0.26	0.28	0.62	0.36	1.686	0.51
US-8748418	ChEMBL	23	0.55	0.56	0.86	0.64	-0.641	-0.394
US-8748435	SureChEMBLccs	146	0.63	0.69	0.83	0.71	-1.03	0.137
US-8748435	ChEMBL	30	0.65	0.67	0.88	0.73	-1.228	-0.242
US-8748435	SureChEMBL	376	0.07	0.63	0.72	0.32	0.896	0.477
US-8748451	SureChEMBLccs	101	0.57	0.76	0.71	0.68	-0.58	1.088
US-8748451	ChEMBL	4	0.73	0.73	0.97	0.8	-1.922	-0.693
US-8748451	SureChEMBL	882	0.06	0.27	0.57	0.21	2.388	0.73

US-8748458	SureChEMBLccs	71	0.66	0.7	0.83	0.73	-1.126	0.174
US-8748458	SureChEMBL	181	0.07	0.26	0.51	0.21	2.627	1.129
US-8748458	ChEMBL	8	0.81	0.81	0.96	0.86	-2.269	-0.409
US-8748624	SureChEMBL	500	0.05	0.4	0.62	0.23	1.899	0.66
US-8748624	SureChEMBLccs	214	0.47	0.73	0.73	0.63	-0.346	0.833
US-8748624	ChEMBL	102	0.73	0.79	0.85	0.79	-1.592	0.267
US-8748626	SureChEMBL	238	0.08	0.28	0.72	0.25	1.721	-0.276
US-8748626	SureChEMBLccs	58	0.59	0.75	0.87	0.73	-1.238	-0.03
US-8748626	ChEMBL	8	0.73	0.73	0.95	0.8	-1.843	-0.555
US-8754075	SureChEMBLccs	102	0.28	0.28	0.68	0.38	1.4	0.105
US-8754075	ChEMBL	50	0.59	0.73	0.84	0.71	-1.071	0.134
US-8754075	SureChEMBL	219	0.1	0.23	0.71	0.25	1.834	-0.305
US-8754089	ChEMBL	28	0.61	0.82	0.88	0.76	-1.496	0.062
US-8754089	SureChEMBL	204	0.06	0.17	0.79	0.2	1.758	-1.009
US-8754089	SureChEMBLccs	59	0.47	0.56	0.81	0.6	-0.251	-0.089
US-8754099	ChEMBL	4	0.74	0.74	0.98	0.81	-2.01	-0.735
US-8754099	SureChEMBL	184	0.05	0.18	0.74	0.19	1.956	-0.647
US-8754099	SureChEMBLccs	26	0.74	0.93	0.88	0.85	-2.075	0.368
US-8754105	ChEMBL	3	0.81	0.81	0.98	0.86	-2.348	-0.547
US-8754105	SureChEMBLccs	22	0.28	0.39	0.91	0.46	0.221	-1.248
US-8754105	SureChEMBL	298	0.04	0.22	0.75	0.19	1.844	-0.634
US-8754113	SureChEMBLccs	606	0.53	0.72	0.74	0.66	-0.506	0.773
US-8754113	ChEMBL	39	0.62	0.71	0.86	0.72	-1.173	-0.032
US-8754113	SureChEMBL	911	0.07	0.28	0.75	0.24	1.626	-0.489
US-8754227	SureChEMBLccs	26	0.74	0.87	0.9	0.83	-2.008	0.1
US-8754227	ChEMBL	4	0.82	0.82	0.97	0.87	-2.357	-0.451
US-8754227	SureChEMBL	94	0.05	0.18	0.85	0.2	1.52	-1.408
US-8759338	ChEMBL	19	0.77	0.89	0.92	0.86	-2.208	0.02
US-8759338	SureChEMBL	217	0.06	0.22	0.82	0.22	1.518	-1.108
US-8759338	SureChEMBLccs	32	0.38	0.82	0.85	0.64	-0.825	0.15
US-8759365	SureChEMBL	532	0.06	0.23	0.8	0.22	1.573	-0.948
US-8759365	SureChEMBLccs	40	0.45	0.79	0.84	0.67	-0.88	0.191
US-8759365	ChEMBL	7	0.82	0.91	0.95	0.89	-2.496	-0.118
US-8759366	SureChEMBL	2974	0.05	0.28	0.8	0.22	1.476	-0.845
US-8759366	SureChEMBLccs	1841	0.46	0.69	0.74	0.62	-0.265	0.672
US-8759366	ChEMBL	39	0.59	0.69	0.87	0.71	-1.093	-0.16
US-8759386	SureChEMBL	198	0.05	0.14	0.59	0.16	2.649	0.305
US-8759386	ChEMBL	21	0.7	0.7	0.9	0.76	-1.5	-0.289
US-8759386	SureChEMBLccs	30	0.65	0.78	0.84	0.75	-1.336	0.273
US-8759532	ChEMBL	216	0.53	0.79	0.76	0.68	-0.755	0.786
US-8759532	SureChEMBL	875	0.06	0.18	0.8	0.21	1.694	-1.057
US-8759532	SureChEMBLccs	415	0.37	0.65	0.65	0.54	0.405	1.161
US-8759537	ChEMBL	17	0.67	0.68	0.9	0.74	-1.379	-0.348
US-8759537	SureChEMBL	611	0.06	0.15	0.82	0.19	1.688	-1.26
US-8759537	SureChEMBLccs	51	0.43	0.62	0.82	0.6	-0.34	-0.049
US-8765727	SureChEMBL	260	0.04	0.12	0.76	0.15	2.047	-0.92
US-8765727	SureChEMBLccs	62	0.25	0.28	0.88	0.39	0.679	-1.295
US-8765727	ChEMBL	14	0.77	0.78	0.91	0.82	-1.902	-0.149
US-8765733	SureChEMBLccs	125	0.62	0.77	0.76	0.71	-0.922	0.79
US-8765733	ChEMBL	20	0.71	0.75	0.89	0.78	-1.605	-0.106
US-8765733	SureChEMBL	300	0.11	0.3	0.71	0.29	1.64	-0.148

US-8765744	SureChEMBLccs	200	0.45	0.56	0.82	0.59	-0.243	-0.169
US-8765744	ChEMBL	106	0.55	0.65	0.78	0.65	-0.542	0.355
US-8765744	SureChEMBL	507	0.04	0.25	0.56	0.18	2.525	0.746
US-8765750	ChEMBL	11	0.84	0.9	0.94	0.89	-2.48	-0.06
US-8765750	SureChEMBL	194	0.07	0.44	0.6	0.26	1.833	0.896
US-8765750	SureChEMBLccs	44	0.47	0.56	0.81	0.6	-0.251	-0.089
US-8765820	SureChEMBL	147	0.05	0.42	0.58	0.23	2.009	0.981
US-8765820	SureChEMBLccs	32	0.68	0.88	0.87	0.8	-1.77	0.298
US-8765820	ChEMBL	5	0.76	0.76	0.96	0.82	-2.027	-0.543
US-8765972	SureChEMBL	269	0.06	0.2	0.38	0.17	3.312	1.893
US-8765972	SureChEMBLccs	145	0.44	0.56	0.69	0.55	0.297	0.726
US-8765972	ChEMBL	19	0.57	0.74	0.9	0.72	-1.285	-0.27
US-8772282	SureChEMBLccs	241	0.42	0.56	0.69	0.55	0.345	0.715
US-8772282	ChEMBL	3	0.8	0.8	0.98	0.86	-2.3	-0.574
US-8772282	SureChEMBL	490	0.06	0.16	0.87	0.2	1.465	-1.584
US-8772283	ChEMBL	54	0.72	0.86	0.85	0.81	-1.738	0.414
US-8772283	SureChEMBL	295	0.1	0.3	0.69	0.27	1.744	-0.015
US-8772283	SureChEMBLccs	155	0.49	0.75	0.8	0.66	-0.72	0.402
US-8772285	ChEMBL	2	0.8	0.8	0.99	0.86	-2.339	-0.643
US-8772285	SureChEMBL	270	0.1	0.24	0.72	0.26	1.77	-0.352
US-8772285	SureChEMBLccs	61	0.53	0.6	0.8	0.63	-0.452	0.098
US-8772288	ChEMBL	35	0.64	0.71	0.8	0.71	-0.983	0.393
US-8772288	SureChEMBLccs	83	0.55	0.7	0.73	0.66	-0.465	0.809
US-8772288	SureChEMBL	571	0.08	0.21	0.74	0.23	1.812	-0.566
US-8772297	SureChEMBLccs	38	0.6	0.67	0.84	0.7	-0.949	0.009
US-8772297	SureChEMBL	165	0.03	0.21	0.62	0.16	2.408	0.238
US-8772297	ChEMBL	15	0.83	0.87	0.95	0.88	-2.423	-0.199
US-8772304	SureChEMBLccs	34	0.66	0.71	0.87	0.74	-1.309	-0.081
US-8772304	SureChEMBL	115	0.1	0.18	0.81	0.24	1.559	-1.105
US-8772304	ChEMBL	31	0.64	0.72	0.88	0.74	-1.325	-0.139
US-8772305	SureChEMBLccs	413	0.3	0.61	0.78	0.52	0.154	0.138
US-8772305	SureChEMBL	1316	0.06	0.17	0.8	0.2	1.719	-1.078
US-8772305	ChEMBL	275	0.56	0.71	0.76	0.67	-0.633	0.629
US-8772316	SureChEMBLccs	267	0.3	0.46	0.66	0.45	0.994	0.644
US-8772316	SureChEMBL	461	0.08	0.19	0.78	0.23	1.702	-0.886
US-8772316	ChEMBL	84	0.61	0.78	0.76	0.71	-0.923	0.806
US-8772480	SureChEMBLccs	530	0.37	0.64	0.61	0.52	0.588	1.416
US-8772480	ChEMBL	321	0.44	0.56	0.71	0.56	0.218	0.587
US-8772480	SureChEMBL	1324	0.04	0.22	0.79	0.19	1.685	-0.911
US-8772481	ChEMBL	21	0.63	0.63	0.86	0.7	-1.003	-0.2
US-8772481	SureChEMBL	119	0.05	0.14	0.89	0.18	1.458	-1.771
US-8772481	SureChEMBLccs	28	0.56	0.79	0.82	0.71	-1.065	0.387
US-8772508	ChEMBL	20	0.65	0.86	0.94	0.81	-1.927	-0.245
US-8772508	SureChEMBL	394	0.08	0.24	0.7	0.24	1.898	-0.224
US-8772508	SureChEMBLccs	48	0.56	0.86	0.89	0.75	-1.512	0.054
US-8772511	SureChEMBL	165	0.09	0.2	0.72	0.23	1.891	-0.444
US-8772511	SureChEMBLccs	42	0.77	0.88	0.88	0.84	-2.025	0.276
US-8772511	ChEMBL	7	0.77	0.8	0.95	0.84	-2.109	-0.382
US-8778412	SureChEMBLccs	61	0.27	0.56	0.82	0.5	0.189	-0.263
US-8778412	ChEMBL	3	0.61	0.61	0.98	0.71	-1.383	-1.085
US-8778412	SureChEMBL	681	0.07	0.16	0.78	0.21	1.798	-0.956

US-8778932	SureChEMBLccs	76	0.31	0.71	0.76	0.55	-0.033	0.498
US-8778932	ChEMBL	5	0.82	0.82	0.96	0.86	-2.317	-0.382
US-8778932	SureChEMBL	149	0.08	0.17	0.85	0.23	1.472	-1.414
US-8778951	SureChEMBLccs	518	0.42	0.56	0.78	0.57	-0.012	0.092
US-8778951	SureChEMBL	813	0.07	0.42	0.61	0.26	1.842	0.783
US-8778951	ChEMBL	346	0.47	0.69	0.72	0.62	-0.21	0.815
US-8778970	SureChEMBL	255	0.05	0.09	0.46	0.13	3.286	1.096
US-8778970	SureChEMBLccs	52	0.52	0.72	0.79	0.67	-0.68	0.422
US-8778970	ChEMBL	12	0.69	0.75	0.9	0.78	-1.597	-0.186
US-8779142	SureChEMBL	687	0.06	0.21	0.81	0.22	1.582	-1.061
US-8779142	SureChEMBLccs	414	0.4	0.56	0.71	0.54	0.314	0.566
US-8779142	ChEMBL	148	0.51	0.64	0.77	0.63	-0.383	0.382
US-8785459	SureChEMBLccs	199	0.56	0.68	0.73	0.65	-0.441	0.771
US-8785459	ChEMBL	123	0.59	0.81	0.79	0.72	-1.066	0.653
US-8785459	SureChEMBL	291	0.08	0.26	0.88	0.26	1.135	-1.427
US-8785464	ChEMBL	67	0.52	0.68	0.86	0.67	-0.861	-0.149
US-8785464	SureChEMBL	248	0.05	0.24	0.77	0.21	1.692	-0.724
US-8785464	SureChEMBLccs	95	0.52	0.68	0.84	0.67	-0.781	-0.011
US-8785467	ChEMBL	20	0.56	0.72	0.92	0.72	-1.292	-0.457
US-8785467	SureChEMBL	342	0.05	0.28	0.77	0.22	1.595	-0.638
US-8785467	SureChEMBLccs	71	0.5	0.71	0.8	0.66	-0.647	0.321
US-8785489	SureChEMBL	307	0.08	0.17	0.85	0.23	1.472	-1.414
US-8785489	SureChEMBLccs	179	0.46	0.67	0.68	0.59	0.022	1.043
US-8785489	ChEMBL	102	0.54	0.55	0.71	0.6	0.002	0.617
US-8785638	SureChEMBLccs	396	0.5	0.69	0.67	0.61	-0.083	1.177
US-8785638	ChEMBL	70	0.61	0.85	0.77	0.74	-1.132	0.889
US-8785638	SureChEMBL	680	0.06	0.63	0.43	0.25	2.07	2.479
US-8791100	SureChEMBLccs	134	0.51	0.7	0.7	0.63	-0.25	0.996
US-8791100	ChEMBL	76	0.51	0.73	0.75	0.65	-0.522	0.715
US-8791100	SureChEMBL	334	0.08	0.28	0.43	0.21	2.872	1.731
US-8791118	ChEMBL	38	0.67	0.89	0.82	0.79	-1.571	0.661
US-8791118	SureChEMBL	235	0.05	0.18	0.58	0.17	2.591	0.461
US-8791118	SureChEMBLccs	118	0.65	0.82	0.8	0.75	-1.274	0.637
US-8791123	SureChEMBL	978	0.07	0.37	0.62	0.25	1.923	0.606
US-8791123	SureChEMBLccs	575	0.46	0.68	0.72	0.61	-0.161	0.788
US-8791123	ChEMBL	208	0.62	0.7	0.85	0.72	-1.109	0.015
US-8791130	SureChEMBL	207	0.05	0.12	0.9	0.18	1.467	-1.884
US-8791130	SureChEMBLccs	61	0.52	0.56	0.83	0.62	-0.45	-0.202
US-8791130	ChEMBL	42	0.62	0.7	0.84	0.71	-1.07	0.084
US-8791131	SureChEMBL	642	0.06	0.18	0.82	0.21	1.615	-1.195
US-8791131	ChEMBL	152	0.52	0.66	0.71	0.62	-0.217	0.845
US-8791131	SureChEMBLccs	247	0.52	0.66	0.71	0.62	-0.217	0.845
US-8791257	ChEMBL	22	0.63	0.69	0.87	0.72	-1.189	-0.14
US-8791257	SureChEMBL	895	0.05	0.28	0.68	0.21	1.952	-0.015
US-8791257	SureChEMBLccs	305	0.44	0.71	0.75	0.62	-0.305	0.635
US-8791268	SureChEMBLccs	35	0.72	0.88	0.87	0.82	-1.866	0.319
US-8791268	SureChEMBL	123	0.08	0.21	0.55	0.21	2.565	0.749
US-8791268	ChEMBL	14	0.73	0.73	0.92	0.79	-1.724	-0.347
US-8791272	SureChEMBL	485	0.05	0.47	0.44	0.22	2.443	2.058
US-8791272	ChEMBL	208	0.56	0.67	0.74	0.65	-0.456	0.68
US-8791272	SureChEMBLccs	279	0.33	0.56	0.75	0.52	0.323	0.253

US-8796244	SureChEMBLccs	477	0.56	0.79	0.72	0.68	-0.668	1.079
US-8796244	ChEMBL	298	0.63	0.8	0.8	0.74	-1.178	0.583
US-8796244	SureChEMBL	960	0.06	0.15	0.8	0.19	1.767	-1.122
US-8796280	SureChEMBLccs	39	0.7	0.8	0.87	0.79	-1.623	0.135
US-8796280	ChEMBL	34	0.7	0.8	0.86	0.78	-1.584	0.204
US-8796280	SureChEMBL	166	0.07	0.22	0.76	0.23	1.732	-0.688
US-8796293	SureChEMBLccs	64	0.56	0.74	0.79	0.69	-0.825	0.486
US-8796293	SureChEMBL	431	0.05	0.24	0.66	0.2	2.128	0.037
US-8796293	ChEMBL	6	0.84	0.85	0.96	0.88	-2.438	-0.307
US-8796295	SureChEMBL	277	0.09	0.22	0.81	0.25	1.486	-1.024
US-8796295	SureChEMBLccs	132	0.49	0.56	0.84	0.61	-0.418	-0.287
US-8796295	ChEMBL	15	0.6	0.6	0.9	0.69	-1.017	-0.558
US-8796296	SureChEMBL	181	0.06	0.28	0.61	0.22	2.205	0.475
US-8796296	SureChEMBLccs	31	0.62	0.7	0.89	0.73	-1.268	-0.262
US-8796296	ChEMBL	26	0.62	0.7	0.88	0.73	-1.228	-0.192
US-8796304	SureChEMBLccs	53	0.28	0.81	0.78	0.56	-0.283	0.561
US-8796304	ChEMBL	16	0.8	0.86	0.94	0.86	-2.287	-0.167
US-8796304	SureChEMBL	200	0.04	0.3	0.63	0.2	2.126	0.369
US-8796310	ChEMBL	68	0.79	0.87	0.87	0.84	-2.009	0.334
US-8796310	SureChEMBL	555	0.07	0.14	0.83	0.2	1.649	-1.346
US-8796310	SureChEMBLccs	193	0.72	0.87	0.81	0.8	-1.603	0.712
US-8796330	ChEMBL	34	0.53	0.84	0.81	0.71	-1.075	0.549
US-8796330	SureChEMBL	551	0.05	0.31	0.65	0.22	1.998	0.258
US-8796330	SureChEMBLccs	55	0.4	0.54	0.73	0.54	0.283	0.385
US-8802663	ChEMBL	42	0.45	0.62	0.8	0.61	-0.309	0.099
US-8802663	SureChEMBLccs	187	0.41	0.61	0.63	0.54	0.486	1.234
US-8802663	SureChEMBL	731	0.05	0.28	0.63	0.21	2.15	0.331
US-8802665	SureChEMBLccs	104	0.46	0.65	0.73	0.6	-0.128	0.654
US-8802665	ChEMBL	40	0.64	0.76	0.81	0.73	-1.144	0.432
US-8802665	SureChEMBL	237	0.07	0.28	0.78	0.25	1.507	-0.696
US-8802672	SureChEMBL	104	0.09	0.19	0.86	0.24	1.36	-1.435
US-8802672	ChEMBL	5	0.87	0.87	0.97	0.9	-2.598	-0.317
US-8802672	SureChEMBLccs	45	0.76	0.79	0.89	0.81	-1.822	0.006
US-8802674	ChEMBL	207	0.5	0.6	0.69	0.59	0.056	0.843
US-8802674	SureChEMBLccs	353	0.49	0.71	0.7	0.62	-0.227	1.007
US-8802674	SureChEMBL	463	0.07	0.65	0.51	0.29	1.68	1.974
US-8802679	SureChEMBL	952	0.08	0.56	0.57	0.29	1.637	1.369
US-8802679	SureChEMBLccs	707	0.47	0.56	0.73	0.58	0.066	0.464
US-8802679	ChEMBL	22	0.68	0.72	0.9	0.76	-1.5	-0.256
US-8802686	SureChEMBL	100	0.06	0.28	0.55	0.21	2.444	0.89
US-8802686	SureChEMBLccs	44	0.39	0.43	0.86	0.52	0.058	-0.759
US-8802686	ChEMBL	14	0.79	0.79	0.92	0.83	-2.013	-0.186
US-8802695	SureChEMBLccs	57	0.56	0.63	0.85	0.67	-0.796	-0.168
US-8802695	ChEMBL	22	0.68	0.84	0.87	0.79	-1.672	0.211
US-8802695	SureChEMBL	462	0.06	0.26	0.7	0.22	1.897	-0.191
US-8802711	ChEMBL	150	0.55	0.75	0.75	0.68	-0.666	0.779
US-8802711	SureChEMBLccs	176	0.55	0.75	0.76	0.68	-0.706	0.71
US-8802711	SureChEMBL	484	0.04	0.28	0.82	0.21	1.42	-0.989
US-8802712	ChEMBL	78	0.66	0.8	0.79	0.75	-1.21	0.668
US-8802712	SureChEMBLccs	107	0.6	0.8	0.77	0.72	-0.987	0.775
US-8802712	SureChEMBL	171	0.1	0.16	0.91	0.24	1.211	-1.84

US-8802721	ChEMBL	6	0.59	0.59	0.95	0.69	-1.167	-0.931
US-8802721	SureChEMBLccs	126	0.5	0.6	0.78	0.62	-0.301	0.221
US-8802721	SureChEMBL	338	0.07	0.2	0.81	0.22	1.582	-1.077
US-8802861	ChEMBL	17	0.64	0.67	0.91	0.73	-1.323	-0.455
US-8802861	SureChEMBLccs	229	0.28	0.38	0.71	0.42	1.038	0.114
US-8802861	SureChEMBL	487	0.06	0.16	0.85	0.2	1.545	-1.446
US-8802864	ChEMBL	11	0.77	0.77	0.94	0.82	-1.996	-0.378
US-8802864	SureChEMBLccs	272	0.59	0.81	0.78	0.72	-1.027	0.722
US-8802864	SureChEMBL	577	0.06	0.22	0.83	0.22	1.478	-1.178
US-8809402	SureChEMBL	221	0.06	0.22	0.53	0.19	2.668	0.899
US-8809402	ChEMBL	32	0.65	0.7	0.84	0.73	-1.142	0.1
US-8809402	SureChEMBLccs	47	0.65	0.68	0.84	0.72	-1.093	0.057
US-8815840	SureChEMBL	1225	0.04	0.28	0.83	0.21	1.381	-1.058
US-8815840	ChEMBL	92	0.5	0.57	0.74	0.6	-0.07	0.432
US-8815840	SureChEMBLccs	770	0.42	0.56	0.68	0.54	0.385	0.784
US-8815845	SureChEMBL	795	0.07	0.17	0.78	0.21	1.774	-0.935
US-8815845	SureChEMBLccs	103	0.59	0.74	0.79	0.7	-0.897	0.502
US-8815845	ChEMBL	5	0.75	0.75	0.97	0.82	-2.019	-0.639
US-8815854	ChEMBL	20	0.69	0.89	0.9	0.82	-1.937	0.117
US-8815854	SureChEMBLccs	86	0.68	0.83	0.85	0.78	-1.569	0.328
US-8815854	SureChEMBL	373	0.06	0.09	0.81	0.16	1.873	-1.321
US-8815881	SureChEMBL	670	0.08	0.65	0.55	0.31	1.498	1.702
US-8815881	ChEMBL	20	0.71	0.78	0.9	0.79	-1.718	-0.111
US-8815881	SureChEMBLccs	440	0.4	0.72	0.76	0.6	-0.273	0.567
US-8815891	SureChEMBL	459	0.07	0.56	0.38	0.25	2.414	2.678
US-8815891	SureChEMBLccs	310	0.34	0.5	0.73	0.5	0.524	0.267
US-8815891	ChEMBL	105	0.52	0.67	0.74	0.64	-0.36	0.659
US-8815901	SureChEMBLccs	234	0.5	0.56	0.67	0.57	0.232	0.895
US-8815901	SureChEMBL	835	0.05	0.14	0.75	0.17	2.014	-0.803
US-8815901	ChEMBL	47	0.6	0.79	0.8	0.72	-1.082	0.546
US-8815918	SureChEMBLccs	159	0.45	0.56	0.74	0.57	0.075	0.385
US-8815918	SureChEMBL	237	0.11	0.17	0.86	0.25	1.361	-1.468
US-8815918	ChEMBL	14	0.62	0.66	0.93	0.72	-1.33	-0.625
US-8815926	SureChEMBLccs	398	0.51	0.64	0.74	0.62	-0.264	0.589
US-8815926	SureChEMBL	1042	0.07	0.14	0.84	0.2	1.609	-1.415
US-8815926	ChEMBL	277	0.63	0.82	0.76	0.73	-1.068	0.903
US-8815934	ChEMBL	38	0.84	0.9	0.9	0.88	-2.321	0.217
US-8815934	SureChEMBLccs	110	0.61	0.75	0.77	0.71	-0.889	0.672
US-8815934	SureChEMBL	348	0.05	0.23	0.76	0.21	1.756	-0.677
US-8822447	ChEMBL	69	0.51	0.56	0.84	0.62	-0.466	-0.276
US-8822447	SureChEMBL	452	0.03	0.23	0.66	0.17	2.2	0.005
US-8822447	SureChEMBLccs	131	0.48	0.56	0.86	0.61	-0.473	-0.43
US-8822458	ChEMBL	11	0.71	0.82	0.92	0.81	-1.894	-0.162
US-8822458	SureChEMBLccs	102	0.67	0.79	0.77	0.74	-1.13	0.79
US-8822458	SureChEMBL	434	0.04	0.23	0.84	0.2	1.462	-1.236
US-8822479	ChEMBL	5	0.81	0.81	0.96	0.86	-2.269	-0.409
US-8822479	SureChEMBLccs	33	0.72	0.79	0.91	0.8	-1.806	-0.153
US-8822479	SureChEMBL	202	0.06	0.15	0.61	0.18	2.521	0.193
US-8822494	SureChEMBL	511	0.05	0.17	0.69	0.18	2.179	-0.322
US-8822494	SureChEMBLccs	73	0.35	0.45	0.66	0.47	0.899	0.648
US-8822494	ChEMBL	26	0.62	0.64	0.88	0.7	-1.083	-0.322

US-8822500	SureChEMBL	510	0.06	0.15	0.72	0.19	2.085	-0.568
US-8822500	SureChEMBLccs	102	0.54	0.83	0.75	0.7	-0.836	0.947
US-8822500	ChEMBL	17	0.69	0.76	0.91	0.78	-1.661	-0.234
US-8822505	SureChEMBL	127	0.06	0.21	0.81	0.22	1.582	-1.061
US-8822505	ChEMBL	4	0.75	0.82	0.98	0.84	-2.228	-0.557
US-8822505	SureChEMBLccs	23	0.5	0.54	0.87	0.62	-0.513	-0.532
US-8822510	ChEMBL	17	0.55	0.55	0.9	0.65	-0.776	-0.692
US-8822510	SureChEMBL	587	0.04	0.23	0.48	0.16	2.891	1.256
US-8822510	SureChEMBLccs	179	0.41	0.59	0.71	0.56	0.217	0.637
US-8822534	ChEMBL	18	0.65	0.7	0.89	0.74	-1.34	-0.246
US-8822534	SureChEMBL	1035	0.05	0.18	0.84	0.2	1.56	-1.339
US-8822534	SureChEMBLccs	508	0.5	0.64	0.75	0.62	-0.279	0.515
US-8829000	SureChEMBL	652	0.1	0.24	0.85	0.27	1.255	-1.252
US-8829000	ChEMBL	9	0.46	0.48	0.93	0.59	-0.509	-1.098
US-8829000	SureChEMBLccs	488	0.32	0.56	0.58	0.47	1.021	1.424
US-8829010	SureChEMBL	261	0.05	0.13	0.85	0.18	1.641	-1.516
US-8829010	ChEMBL	39	0.69	0.69	0.84	0.74	-1.213	0.099
US-8829010	SureChEMBLccs	55	0.62	0.78	0.81	0.73	-1.145	0.465
US-8829190	SureChEMBLccs	32	0.42	0.55	0.85	0.58	-0.266	-0.414
US-8829190	SureChEMBL	109	0.06	0.14	0.87	0.19	1.514	-1.628
US-8829190	ChEMBL	26	0.72	0.77	0.88	0.79	-1.638	0.011
US-8829199	SureChEMBLccs	780	0.27	0.37	0.62	0.4	1.444	0.71
US-8829199	SureChEMBL	1249	0.07	0.16	0.81	0.21	1.679	-1.164
US-8829199	ChEMBL	109	0.66	0.79	0.83	0.76	-1.344	0.369
US-8829200	SureChEMBLccs	44	0.71	0.8	0.87	0.79	-1.647	0.14
US-8829200	ChEMBL	29	0.79	0.86	0.9	0.85	-2.104	0.104
US-8829200	SureChEMBL	112	0.13	0.21	0.77	0.28	1.573	-0.748
US-8835436	SureChEMBLccs	439	0.59	0.83	0.73	0.71	-0.877	1.112
US-8835436	ChEMBL	54	0.59	0.69	0.8	0.69	-0.815	0.324
US-8835436	SureChEMBL	682	0.11	0.71	0.56	0.35	1.24	1.779
US-8835445	ChEMBL	39	0.51	0.56	0.85	0.62	-0.506	-0.345
US-8835445	SureChEMBL	116	0.06	0.22	0.8	0.22	1.597	-0.97
US-8835445	SureChEMBLccs	54	0.51	0.56	0.83	0.62	-0.426	-0.207
US-8835464	SureChEMBL	1149	0.06	0.18	0.79	0.2	1.734	-0.987
US-8835464	ChEMBL	60	0.41	0.55	0.74	0.55	0.195	0.342
US-8835464	SureChEMBLccs	121	0.4	0.55	0.67	0.53	0.496	0.821
US-8835465	ChEMBL	3	0.73	0.73	0.98	0.81	-1.962	-0.762
US-8835465	SureChEMBLccs	125	0.54	0.8	0.74	0.68	-0.724	0.952
US-8835465	SureChEMBL	417	0.05	0.24	0.5	0.18	2.763	1.144
US-8835470	SureChEMBLccs	213	0.64	0.88	0.78	0.76	-1.317	0.9
US-8835470	SureChEMBL	501	0.03	0.47	0.66	0.21	1.618	0.525
US-8835470	ChEMBL	22	0.73	0.82	0.92	0.82	-1.942	-0.152
US-8835472	SureChEMBL	1155	0.06	0.64	0.45	0.26	1.967	2.362
US-8835472	ChEMBL	655	0.52	0.64	0.76	0.63	-0.367	0.456
US-8835472	SureChEMBLccs	701	0.49	0.65	0.78	0.63	-0.399	0.324
US-8835659	ChEMBL	6	0.56	0.56	0.95	0.67	-1.022	-1.011
US-8835659	SureChEMBLccs	31	0.41	0.49	0.81	0.55	0.063	-0.272
US-8835659	SureChEMBL	86	0.08	0.28	0.81	0.26	1.364	-0.899
US-8841288	SureChEMBLccs	119	0.67	0.81	0.79	0.75	-1.258	0.695
US-8841288	ChEMBL	10	0.77	0.8	0.92	0.83	-1.99	-0.175
US-8841288	SureChEMBL	164	0.09	0.22	0.84	0.26	1.367	-1.231

US-8841312	SureChEMBLccs	529	0.56	0.7	0.73	0.66	-0.489	0.814
US-8841312	SureChEMBL	1260	0.05	0.14	0.83	0.18	1.696	-1.356
US-8841312	ChEMBL	476	0.57	0.74	0.73	0.68	-0.611	0.906
US-8841450	ChEMBL	2	0.85	0.85	0.99	0.89	-2.581	-0.509
US-8841450	SureChEMBLccs	6	0.75	0.78	0.96	0.83	-2.052	-0.505
US-8841450	SureChEMBL	267	0.05	0.28	0.74	0.22	1.714	-0.43
US-8846000	SureChEMBLccs	34	0.76	0.88	0.85	0.83	-1.882	0.478
US-8846000	SureChEMBL	335	0.07	0.38	0.67	0.26	1.701	0.282
US-8846000	ChEMBL	29	0.76	0.88	0.85	0.83	-1.882	0.478
US-8846654	ChEMBL	7	0.77	0.77	0.94	0.82	-1.996	-0.378
US-8846654	SureChEMBL	527	0.06	0.73	0.58	0.29	1.233	1.657
US-8846654	SureChEMBLccs	306	0.57	0.7	0.77	0.67	-0.672	0.543
US-8846657	SureChEMBL	1688	0.04	0.19	0.81	0.18	1.678	-1.115
US-8846657	ChEMBL	232	0.45	0.66	0.83	0.63	-0.525	-0.021
US-8846657	SureChEMBLccs	759	0.38	0.63	0.76	0.57	-0.007	0.362
US-8846658	SureChEMBLccs	372	0.28	0.43	0.7	0.44	0.957	0.291
US-8846658	SureChEMBL	752	0.07	0.18	0.8	0.22	1.67	-1.051
US-8846658	ChEMBL	174	0.56	0.68	0.75	0.66	-0.52	0.633
US-8846673	SureChEMBL	646	0.05	0.2	0.82	0.2	1.591	-1.157
US-8846673	ChEMBL	37	0.48	0.53	0.84	0.6	-0.321	-0.357
US-8846673	SureChEMBLccs	434	0.37	0.56	0.71	0.53	0.386	0.551
US-8846696	ChEMBL	10	0.88	0.89	0.96	0.91	-2.631	-0.199
US-8846696	SureChEMBL	106	0.08	0.19	0.69	0.22	2.059	-0.263
US-8846696	SureChEMBLccs	20	0.57	0.87	0.9	0.76	-1.6	0.012
US-8846698	ChEMBL	57	0.57	0.68	0.85	0.69	-0.941	-0.054
US-8846698	SureChEMBL	1090	0.05	0.17	0.84	0.19	1.584	-1.36
US-8846698	SureChEMBLccs	185	0.47	0.66	0.8	0.63	-0.454	0.197
US-8846699	SureChEMBLccs	480	0.54	0.79	0.71	0.67	-0.581	1.137
US-8846699	SureChEMBL	676	0.07	0.22	0.89	0.24	1.216	-1.588
US-8846699	ChEMBL	146	0.7	0.89	0.81	0.8	-1.604	0.745
US-8846711	SureChEMBLccs	150	0.53	0.8	0.74	0.68	-0.7	0.946
US-8846711	ChEMBL	19	0.59	0.67	0.86	0.7	-1.004	-0.135
US-8846711	SureChEMBL	558	0.07	0.1	0.7	0.17	2.261	-0.533
US-8846719	SureChEMBL	741	0.05	0.62	0.4	0.23	2.238	2.66
US-8846719	ChEMBL	319	0.55	0.81	0.69	0.67	-0.574	1.324
US-8846719	SureChEMBLccs	388	0.55	0.81	0.68	0.67	-0.534	1.394
US-8846730	ChEMBL	63	0.71	0.82	0.88	0.8	-1.736	0.114
US-8846730	SureChEMBLccs	227	0.64	0.87	0.8	0.76	-1.372	0.74
US-8846730	SureChEMBL	410	0.06	0.81	0.65	0.32	0.761	1.346
US-8846741	SureChEMBLccs	51	0.53	0.56	0.89	0.64	-0.712	-0.612
US-8846741	ChEMBL	7	0.63	0.63	0.97	0.73	-1.44	-0.962
US-8846741	SureChEMBL	408	0.07	0.12	0.68	0.18	2.292	-0.351
US-8846746	SureChEMBL	1118	0.05	0.28	0.76	0.22	1.634	-0.568
US-8846746	ChEMBL	506	0.53	0.72	0.73	0.65	-0.466	0.842
US-8846746	SureChEMBLccs	629	0.48	0.76	0.71	0.64	-0.364	1.041
US-8853193	SureChEMBL	673	0.06	0.2	0.82	0.21	1.567	-1.152
US-8853193	SureChEMBLccs	204	0.56	0.63	0.72	0.63	-0.28	0.732
US-8853193	ChEMBL	125	0.56	0.56	0.74	0.61	-0.189	0.442
US-8853203	SureChEMBL	659	0.06	0.28	0.74	0.23	1.69	-0.425
US-8853203	SureChEMBLccs	253	0.52	0.71	0.77	0.66	-0.576	0.539
US-8853203	ChEMBL	180	0.56	0.65	0.76	0.65	-0.487	0.499

US-8853207	SureChEMBLccs	103	0.55	0.73	0.81	0.69	-0.856	0.321
US-8853207	SureChEMBL	255	0.08	0.28	0.73	0.25	1.681	-0.345
US-8853207	ChEMBL	5	0.65	0.65	0.96	0.74	-1.496	-0.839
US-8853212	SureChEMBL	387	0.07	0.2	0.75	0.22	1.82	-0.662
US-8853212	ChEMBL	14	0.69	0.71	0.92	0.77	-1.579	-0.411
US-8853212	SureChEMBLccs	32	0.57	0.64	0.85	0.68	-0.844	-0.141
US-8853215	ChEMBL	17	0.64	0.74	0.88	0.75	-1.374	-0.095
US-8853215	SureChEMBLccs	237	0.6	0.68	0.77	0.68	-0.696	0.515
US-8853215	SureChEMBL	768	0.06	0.28	0.73	0.23	1.729	-0.356
US-8853224	SureChEMBLccs	285	0.25	0.38	0.68	0.4	1.229	0.306
US-8853224	SureChEMBL	536	0.07	0.19	0.64	0.2	2.281	0.077
US-8853224	ChEMBL	35	0.48	0.56	0.89	0.62	-0.592	-0.638
US-8853226	SureChEMBL	436	0.07	0.16	0.8	0.21	1.719	-1.095
US-8853226	ChEMBL	10	0.77	0.8	0.95	0.84	-2.109	-0.382
US-8853226	SureChEMBLccs	151	0.42	0.46	0.78	0.53	0.231	-0.124
US-8853227	SureChEMBL	167	0.06	0.09	0.76	0.16	2.072	-0.975
US-8853227	SureChEMBLccs	15	0.11	0.12	0.87	0.23	1.442	-1.645
US-8853227	ChEMBL	2	0.67	0.67	0.99	0.76	-1.712	-0.993
US-8853228	SureChEMBL	222	0.07	0.14	0.73	0.19	2.045	-0.654
US-8853228	ChEMBL	10	0.68	0.69	0.92	0.76	-1.507	-0.46
US-8853228	SureChEMBLccs	53	0.17	0.24	0.78	0.32	1.364	-0.731
US-8853242	SureChEMBLccs	899	0.41	0.56	0.69	0.54	0.369	0.71
US-8853242	ChEMBL	18	0.68	0.76	0.92	0.78	-1.677	-0.308
US-8853242	SureChEMBL	1024	0.07	0.28	0.78	0.25	1.507	-0.696
US-8853258	ChEMBL	22	0.66	0.79	0.89	0.77	-1.583	-0.046
US-8853258	SureChEMBL	328	0.05	0.56	0.44	0.23	2.224	2.253
US-8853258	SureChEMBLccs	152	0.34	0.56	0.69	0.51	0.537	0.674
US-8859534	SureChEMBLccs	72	0.64	0.77	0.81	0.74	-1.169	0.454
US-8859534	ChEMBL	53	0.65	0.69	0.82	0.72	-1.038	0.217
US-8859534	SureChEMBL	401	0.05	0.28	0.52	0.19	2.587	1.093
US-8859545	SureChEMBL	537	0.06	0.34	0.7	0.24	1.703	-0.018
US-8859545	SureChEMBLccs	146	0.49	0.65	0.78	0.63	-0.399	0.324
US-8859545	ChEMBL	68	0.62	0.71	0.81	0.71	-0.975	0.314
US-8859586	SureChEMBLccs	104	0.49	0.56	0.88	0.62	-0.577	-0.563
US-8859586	ChEMBL	32	0.79	0.87	0.89	0.85	-2.089	0.195
US-8859586	SureChEMBL	373	0.07	0.28	0.62	0.23	2.142	0.411
US-8859596	SureChEMBLccs	530	0.44	0.62	0.71	0.58	0.072	0.717
US-8859596	ChEMBL	104	0.57	0.6	0.82	0.65	-0.628	-0.02
US-8859596	SureChEMBL	897	0.07	0.83	0.32	0.26	1.997	3.679
US-8865686	SureChEMBLccs	34	0.68	0.92	0.9	0.83	-1.986	0.177
US-8865686	ChEMBL	2	0.84	0.84	0.99	0.89	-2.532	-0.536
US-8865686	SureChEMBL	77	0.09	0.84	0.71	0.38	0.378	1.012
US-8865706	ChEMBL	26	0.67	0.79	0.88	0.78	-1.567	0.029
US-8865706	SureChEMBL	499	0.04	0.21	0.78	0.19	1.749	-0.864
US-8865706	SureChEMBLccs	123	0.53	0.8	0.72	0.67	-0.62	1.085
US-8865714	SureChEMBL	179	0.04	0.08	0.78	0.14	2.064	-1.145
US-8865714	ChEMBL	9	0.79	0.84	0.96	0.86	-2.293	-0.354
US-8865714	SureChEMBLccs	47	0.39	0.47	0.77	0.52	0.318	-0.049
US-8865911	ChEMBL	59	0.6	0.8	0.78	0.72	-1.026	0.706
US-8865911	SureChEMBLccs	262	0.47	0.77	0.77	0.65	-0.602	0.643
US-8865911	SureChEMBL	856	0.07	0.17	0.85	0.22	1.496	-1.419

US-8871728	ChEMBL	23	0.56	0.64	0.86	0.68	-0.86	-0.215
US-8871728	SureChEMBLccs	200	0.28	0.56	0.6	0.45	1.038	1.265
US-8871728	SureChEMBL	785	0.07	0.28	0.8	0.25	1.428	-0.835
US-8871738	SureChEMBLccs	52	0.57	0.73	0.8	0.69	-0.864	0.4
US-8871738	SureChEMBL	586	0.07	0.22	0.7	0.22	1.97	-0.273
US-8871738	ChEMBL	15	0.71	0.76	0.93	0.79	-1.788	-0.362
US-8871778	SureChEMBLccs	239	0.63	0.77	0.77	0.72	-0.986	0.726
US-8871778	SureChEMBL	420	0.06	0.17	0.89	0.21	1.362	-1.701
US-8871778	ChEMBL	20	0.76	0.86	0.87	0.83	-1.913	0.296
US-8871783	SureChEMBLccs	58	0.47	0.79	0.7	0.64	-0.373	1.17
US-8871783	SureChEMBL	255	0.06	0.12	0.82	0.18	1.761	-1.325
US-8871783	ChEMBL	26	0.65	0.73	0.85	0.74	-1.254	0.096
US-8871790	SureChEMBLccs	761	0.32	0.64	0.62	0.5	0.668	1.321
US-8871790	SureChEMBL	1347	0.05	0.31	0.75	0.23	1.601	-0.434
US-8871790	ChEMBL	472	0.43	0.56	0.72	0.56	0.202	0.513
US-8871934	SureChEMBLccs	738	0.44	0.63	0.81	0.61	-0.349	0.047
US-8871934	SureChEMBL	1076	0.04	0.16	0.72	0.17	2.108	-0.557
US-8871934	ChEMBL	587	0.54	0.62	0.75	0.63	-0.327	0.492
US-8877741	ChEMBL	454	0.6	0.73	0.76	0.69	-0.777	0.693
US-8877741	SureChEMBLccs	514	0.59	0.75	0.77	0.7	-0.841	0.662
US-8877741	SureChEMBL	898	0.07	0.56	0.58	0.28	1.621	1.294
US-8877757	SureChEMBL	439	0.06	0.23	0.76	0.22	1.732	-0.672
US-8877757	ChEMBL	7	0.71	0.71	0.94	0.78	-1.707	-0.539
US-8877757	SureChEMBLccs	209	0.56	0.74	0.7	0.66	-0.468	1.109
US-8877775	ChEMBL	25	0.74	0.76	0.94	0.81	-1.9	-0.415
US-8877775	SureChEMBLccs	57	0.54	0.65	0.84	0.67	-0.756	-0.066
US-8877775	SureChEMBL	249	0.06	0.12	0.82	0.18	1.761	-1.325
US-8877778	SureChEMBL	319	0.07	0.85	0.53	0.32	1.116	2.269
US-8877778	SureChEMBLccs	201	0.62	0.86	0.8	0.75	-1.299	0.708
US-8877778	ChEMBL	95	0.67	0.89	0.79	0.78	-1.452	0.868
US-8877786	ChEMBL	3	0.64	0.64	0.98	0.74	-1.528	-1.004
US-8877786	SureChEMBL	457	0.08	0.33	0.59	0.25	2.116	0.732
US-8877786	SureChEMBLccs	111	0.57	0.73	0.8	0.69	-0.864	0.4
US-8877798	ChEMBL	25	0.67	0.67	0.92	0.74	-1.434	-0.508
US-8877798	SureChEMBLccs	280	0.22	0.69	0.6	0.45	0.866	1.516
US-8877798	SureChEMBL	507	0.06	0.16	0.77	0.19	1.862	-0.892
US-8877944	SureChEMBLccs	150	0.44	0.56	0.81	0.58	-0.179	-0.105
US-8877944	ChEMBL	51	0.56	0.76	0.9	0.73	-1.309	-0.232
US-8877944	SureChEMBL	272	0.05	0.18	0.76	0.19	1.877	-0.785
US-8883759	SureChEMBLccs	54	0.52	0.86	0.84	0.72	-1.218	0.379
US-8883759	ChEMBL	20	0.75	0.86	0.91	0.84	-2.048	0.014
US-8883759	SureChEMBL	169	0.06	0.14	0.57	0.17	2.704	0.448
US-8889668	SureChEMBLccs	88	0.48	0.56	0.72	0.58	0.082	0.539
US-8889668	SureChEMBL	352	0.04	0.2	0.63	0.17	2.368	0.153
US-8889668	ChEMBL	26	0.61	0.63	0.87	0.69	-0.995	-0.28
US-8889671	SureChEMBLccs	306	0.45	0.55	0.73	0.57	0.138	0.432
US-8889671	SureChEMBL	596	0.05	0.18	0.62	0.18	2.432	0.184
US-8889671	ChEMBL	74	0.8	0.85	0.87	0.84	-1.985	0.295
US-8889672	SureChEMBLccs	34	0.52	0.57	0.82	0.62	-0.435	-0.111
US-8889672	ChEMBL	7	0.53	0.53	0.94	0.64	-0.838	-1.023
US-8889672	SureChEMBL	345	0.05	0.23	0.76	0.21	1.756	-0.677

US-8889684	ChEMBL	8	0.82	0.82	0.95	0.86	-2.277	-0.313
US-8889684	SureChEMBL	504	0.06	0.16	0.76	0.19	1.902	-0.823
US-8889684	SureChEMBLccs	36	0.6	0.72	0.83	0.71	-1.031	0.187
US-8889708	ChEMBL	25	0.63	0.71	0.87	0.73	-1.237	-0.096
US-8889708	SureChEMBL	283	0.06	0.15	0.8	0.19	1.767	-1.122
US-8889708	SureChEMBLccs	46	0.56	0.69	0.79	0.67	-0.703	0.378
US-8889724	ChEMBL	3	0.85	0.85	0.98	0.89	-2.541	-0.44
US-8889724	SureChEMBL	89	0.07	0.28	0.6	0.23	2.221	0.549
US-8889724	SureChEMBLccs	13	0.63	0.8	0.89	0.77	-1.535	-0.04
US-8889870	SureChEMBL	407	0.07	0.39	0.65	0.26	1.756	0.442
US-8889870	ChEMBL	66	0.59	0.68	0.76	0.67	-0.632	0.579
US-8889870	SureChEMBLccs	245	0.46	0.67	0.66	0.59	0.101	1.182
US-8894989	SureChEMBLccs	90	0.33	0.36	0.64	0.42	1.244	0.581
US-8894989	ChEMBL	47	0.56	0.75	0.77	0.69	-0.769	0.646
US-8894989	SureChEMBL	268	0.04	0.13	0.86	0.16	1.626	-1.591
US-8895549	SureChEMBLccs	47	0.66	0.87	0.81	0.77	-1.459	0.681
US-8895549	SureChEMBL	533	0.06	0.21	0.78	0.21	1.701	-0.853
US-8895549	ChEMBL	35	0.7	0.88	0.82	0.8	-1.619	0.654
US-8895581	ChEMBL	75	0.64	0.68	0.8	0.7	-0.911	0.328
US-8895581	SureChEMBLccs	201	0.28	0.35	0.6	0.39	1.547	0.81
US-8895581	SureChEMBL	732	0.06	0.18	0.79	0.2	1.734	-0.987
US-8895589	ChEMBL	48	0.57	0.65	0.82	0.67	-0.749	0.089
US-8895589	SureChEMBLccs	93	0.55	0.65	0.76	0.65	-0.463	0.493
US-8895589	SureChEMBL	272	0.06	0.21	0.52	0.19	2.732	0.946
US-8895592	SureChEMBLccs	89	0.53	0.7	0.78	0.66	-0.616	0.453
US-8895592	SureChEMBL	267	0.05	0.15	0.84	0.18	1.633	-1.404
US-8895592	ChEMBL	56	0.59	0.63	0.84	0.68	-0.828	-0.083
US-8895745	SureChEMBLccs	641	0.5	0.77	0.7	0.65	-0.396	1.143
US-8895745	SureChEMBL	1096	0.04	0.16	0.82	0.17	1.712	-1.249
US-8895745	ChEMBL	17	0.76	0.78	0.89	0.81	-1.798	-0.015
US-8901310	ChEMBL	11	0.8	0.86	0.94	0.86	-2.287	-0.167
US-8901310	SureChEMBL	312	0.07	0.28	0.8	0.25	1.428	-0.835
US-8901310	SureChEMBLccs	20	0.72	0.79	0.88	0.79	-1.687	0.055
US-8906911	SureChEMBLccs	82	0.49	0.65	0.82	0.64	-0.557	0.047
US-8906911	SureChEMBL	724	0.06	0.22	0.72	0.21	1.915	-0.416
US-8906911	ChEMBL	66	0.64	0.9	0.86	0.79	-1.682	0.39
US-8906933	ChEMBL	10	0.83	0.83	0.95	0.87	-2.326	-0.286
US-8906933	SureChEMBLccs	178	0.38	0.66	0.76	0.58	-0.079	0.427
US-8906933	SureChEMBL	234	0.08	0.26	0.86	0.26	1.214	-1.288
US-8906943	ChEMBL	3	0.64	0.64	0.98	0.74	-1.528	-1.004
US-8906943	SureChEMBL	84	0.11	0.36	0.75	0.31	1.336	-0.295
US-8906943	SureChEMBLccs	5	0.54	0.54	0.96	0.65	-0.966	-1.134
US-8907086	SureChEMBL	366	0.06	0.28	0.74	0.23	1.69	-0.425
US-8907086	SureChEMBLccs	14	0.48	0.77	0.9	0.69	-1.142	-0.252
US-8907086	ChEMBL	8	0.77	0.77	0.95	0.83	-2.036	-0.447
US-8912173	ChEMBL	119	0.53	0.56	0.83	0.63	-0.474	-0.197
US-8912173	SureChEMBLccs	167	0.5	0.56	0.82	0.61	-0.363	-0.143
US-8912173	SureChEMBL	944	0.07	0.19	0.8	0.22	1.646	-1.03
US-8912181	SureChEMBL	310	0.07	0.18	0.81	0.22	1.631	-1.121
US-8912181	SureChEMBLccs	149	0.51	0.56	0.76	0.6	-0.149	0.278
US-8912181	ChEMBL	115	0.52	0.62	0.76	0.63	-0.318	0.413

US-8912188	SureChEMBL	944	0.05	0.2	0.86	0.2	1.432	-1.434
US-8912188	SureChEMBLccs	565	0.42	0.56	0.69	0.55	0.345	0.715
US-8912188	ChEMBL	145	0.55	0.63	0.78	0.65	-0.494	0.312
US-8912213	SureChEMBL	175	0.05	0.07	0.72	0.14	2.303	-0.747
US-8912213	ChEMBL	5	0.73	0.74	0.97	0.81	-1.946	-0.671
US-8912213	SureChEMBLccs	59	0.45	0.56	0.75	0.57	0.035	0.316
US-8912221	ChEMBL	42	0.59	0.61	0.85	0.67	-0.819	-0.195
US-8912221	SureChEMBL	169	0.04	0.13	0.78	0.16	1.943	-1.037
US-8912221	SureChEMBLccs	51	0.52	0.64	0.87	0.66	-0.803	-0.305
US-8916561	SureChEMBLccs	82	0.45	0.67	0.73	0.6	-0.153	0.692
US-8916561	ChEMBL	8	0.66	0.72	0.94	0.76	-1.611	-0.543
US-8916561	SureChEMBL	331	0.05	0.23	0.59	0.19	2.43	0.5
US-8916574	SureChEMBLccs	10	0.84	0.84	0.95	0.88	-2.374	-0.259
US-8916574	ChEMBL	4	0.91	0.91	0.98	0.93	-2.831	-0.279
US-8916574	SureChEMBL	97	0.06	0.19	0.72	0.2	1.988	-0.481
US-8916576	SureChEMBLccs	249	0.42	0.62	0.68	0.56	0.239	0.914
US-8916576	SureChEMBL	560	0.04	0.56	0.36	0.2	2.566	2.801
US-8916576	ChEMBL	42	0.7	0.77	0.88	0.78	-1.59	0.001
US-8916577	ChEMBL	11	0.73	0.83	0.92	0.82	-1.967	-0.13
US-8916577	SureChEMBL	324	0.05	0.28	0.76	0.22	1.634	-0.568
US-8916577	SureChEMBLccs	183	0.35	0.56	0.67	0.51	0.592	0.817
US-8916594	SureChEMBL	343	0.06	0.16	0.87	0.2	1.465	-1.584
US-8916594	SureChEMBLccs	167	0.55	0.64	0.82	0.66	-0.677	0.056
US-8916594	ChEMBL	25	0.63	0.63	0.9	0.71	-1.162	-0.477
US-8921368	SureChEMBLccs	553	0.27	0.56	0.67	0.47	0.784	0.776
US-8921368	SureChEMBL	914	0.06	0.56	0.43	0.24	2.24	2.327
US-8921368	ChEMBL	52	0.56	0.73	0.8	0.69	-0.84	0.395
US-8921370	SureChEMBLccs	292	0.55	0.6	0.83	0.65	-0.619	-0.099
US-8921370	SureChEMBL	387	0.05	0.67	0.61	0.27	1.283	1.315
US-8921370	ChEMBL	9	0.67	0.68	0.95	0.76	-1.578	-0.694
US-8921373	SureChEMBL	1807	0.06	0.18	0.81	0.21	1.655	-1.126
US-8921373	ChEMBL	19	0.47	0.53	0.88	0.6	-0.456	-0.639
US-8921373	SureChEMBLccs	434	0.3	0.56	0.65	0.48	0.791	0.93
US-8921389	SureChEMBL	700	0.08	0.31	0.77	0.27	1.45	-0.557
US-8921389	SureChEMBLccs	401	0.36	0.56	0.71	0.52	0.41	0.546
US-8921389	ChEMBL	161	0.56	0.72	0.79	0.68	-0.776	0.443
US-8921397	ChEMBL	88	0.76	0.89	0.85	0.83	-1.906	0.5
US-8921397	SureChEMBLccs	267	0.53	0.62	0.81	0.64	-0.541	0.072
US-8921397	SureChEMBL	379	0.07	0.73	0.46	0.29	1.685	2.493
US-8921404	ChEMBL	7	0.68	0.68	0.94	0.76	-1.562	-0.62
US-8921404	SureChEMBLccs	8	0.62	0.82	0.96	0.79	-1.837	-0.486
US-8921404	SureChEMBL	111	0.07	0.24	0.72	0.23	1.842	-0.368
US-8921410	ChEMBL	67	0.5	0.74	0.8	0.67	-0.72	0.386
US-8921410	SureChEMBL	187	0.07	0.65	0.62	0.3	1.244	1.213
US-8921410	SureChEMBLccs	83	0.5	0.74	0.78	0.66	-0.641	0.524
US-8921412	SureChEMBL	204	0.06	0.18	0.59	0.19	2.527	0.397
US-8921412	ChEMBL	9	0.88	0.88	0.94	0.9	-2.527	-0.082
US-8921412	SureChEMBLccs	18	0.56	0.69	0.92	0.71	-1.219	-0.522
US-8921424	SureChEMBL	111	0.08	0.16	0.88	0.22	1.378	-1.643
US-8921424	ChEMBL	26	0.76	0.84	0.9	0.83	-1.983	0.045
US-8921424	SureChEMBLccs	57	0.64	0.84	0.85	0.77	-1.497	0.329

US-8921559	SureChEMBL	288	0.07	0.36	0.78	0.27	1.313	-0.523
US-8921559	ChEMBL	53	0.72	0.89	0.84	0.81	-1.771	0.548
US-8921559	SureChEMBLccs	133	0.7	0.82	0.81	0.77	-1.434	0.594
US-8927507	SureChEMBL	143	0.04	0.07	0.64	0.12	2.644	-0.198
US-8927507	SureChEMBLccs	44	0.36	0.44	0.83	0.51	0.225	-0.545
US-8927507	ChEMBL	8	0.69	0.69	0.95	0.77	-1.65	-0.662
US-8927520	SureChEMBL	100	0.06	0.56	0.68	0.28	1.248	0.597
US-8927520	ChEMBL	2	0.92	0.92	0.99	0.94	-2.919	-0.321
US-8927520	SureChEMBLccs	50	0.48	0.56	0.84	0.61	-0.394	-0.292
US-8927539	SureChEMBLccs	27	0.39	0.64	0.86	0.6	-0.452	-0.304
US-8927539	ChEMBL	10	0.6	0.6	0.91	0.69	-1.057	-0.627
US-8927539	SureChEMBL	143	0.07	0.33	0.71	0.25	1.663	-0.104
US-8927543	ChEMBL	138	0.72	0.77	0.89	0.79	-1.678	-0.058
US-8927543	SureChEMBLccs	268	0.65	0.83	0.85	0.77	-1.497	0.312
US-8927543	SureChEMBL	568	0.06	0.21	0.78	0.21	1.701	-0.853
US-8927577	ChEMBL	12	0.69	0.84	0.93	0.81	-1.934	-0.199
US-8927577	SureChEMBL	255	0.09	0.2	0.65	0.23	2.169	0.04
US-8927577	SureChEMBLccs	57	0.45	0.6	0.86	0.61	-0.499	-0.359
US-8927730	ChEMBL	4	0.83	0.83	0.97	0.87	-2.405	-0.424
US-8927730	SureChEMBL	35	0.15	0.33	0.84	0.35	0.956	-0.962
US-8927730	SureChEMBLccs	4	0.83	0.83	0.97	0.87	-2.405	-0.424
US-8933040	SureChEMBL	169	0.04	0.2	0.64	0.17	2.329	0.084
US-8933040	SureChEMBLccs	57	0.49	0.52	0.8	0.59	-0.162	-0.096
US-8933040	ChEMBL	6	0.78	0.8	0.96	0.84	-2.172	-0.446
US-8933079	SureChEMBL	194	0.08	0.22	0.83	0.24	1.43	-1.167
US-8933079	ChEMBL	72	0.6	0.76	0.75	0.7	-0.81	0.827
US-8933079	SureChEMBLccs	126	0.56	0.65	0.78	0.66	-0.566	0.36
US-8933099	SureChEMBLccs	212	0.47	0.62	0.82	0.62	-0.436	-0.029
US-8933099	ChEMBL	112	0.71	0.78	0.83	0.77	-1.44	0.374
US-8933099	SureChEMBL	424	0.05	0.8	0.4	0.25	1.801	3.05
US-8933221	SureChEMBLccs	70	0.43	0.51	0.84	0.57	-0.153	-0.426
US-8933221	SureChEMBL	782	0.07	0.2	0.8	0.22	1.622	-1.008
US-8933221	ChEMBL	28	0.53	0.55	0.85	0.63	-0.53	-0.357
US-8933224	SureChEMBLccs	189	0.33	0.51	0.69	0.49	0.682	0.56
US-8933224	ChEMBL	162	0.52	0.59	0.75	0.61	-0.206	0.417
US-8933224	SureChEMBL	559	0.05	0.23	0.8	0.21	1.597	-0.954
US-8937058	SureChEMBL	180	0.06	0.39	0.84	0.27	1.026	-0.878
US-8937058	SureChEMBLccs	79	0.64	0.67	0.85	0.71	-1.085	-0.039
US-8937058	ChEMBL	17	0.74	0.74	0.93	0.8	-1.812	-0.389
US-8937070	SureChEMBL	929	0.04	0.22	0.46	0.16	2.994	1.373
US-8937070	ChEMBL	4	0.79	0.79	0.98	0.85	-2.251	-0.601
US-8937070	SureChEMBLccs	346	0.53	0.62	0.76	0.63	-0.342	0.418
US-8937084	SureChEMBL	132	0.09	0.13	0.81	0.21	1.704	-1.219
US-8937084	SureChEMBLccs	61	0.42	0.51	0.82	0.56	-0.049	-0.293
US-8937084	ChEMBL	15	0.59	0.68	0.89	0.71	-1.148	-0.321
US-8937092	SureChEMBLccs	180	0.56	0.73	0.71	0.66	-0.483	1.018
US-8937092	SureChEMBL	343	0.13	0.34	0.74	0.32	1.376	-0.258
US-8937092	ChEMBL	22	0.75	0.89	0.9	0.84	-2.081	0.148
US-8940719	SureChEMBL	154	0.04	0.09	0.8	0.14	1.961	-1.262
US-8940719	SureChEMBLccs	39	0.73	0.82	0.89	0.81	-1.823	0.056
US-8940719	ChEMBL	13	0.74	0.79	0.92	0.81	-1.893	-0.212

US-8940720	SureChEMBLccs	377	0.2	0.36	0.79	0.38	0.961	-0.525
US-8940720	ChEMBL	23	0.52	0.52	0.84	0.61	-0.393	-0.358
US-8940720	SureChEMBL	1420	0.04	0.17	0.76	0.17	1.925	-0.812
US-8940736	SureChEMBLccs	613	0.34	0.78	0.67	0.56	0.082	1.289
US-8940736	SureChEMBL	1152	0.05	0.42	0.57	0.23	2.048	1.05
US-8940736	ChEMBL	49	0.66	0.72	0.86	0.74	-1.294	0.01
US-8940744	SureChEMBLccs	100	0.67	0.82	0.87	0.78	-1.6	0.163
US-8940744	SureChEMBL	951	0.06	0.23	0.8	0.22	1.573	-0.948
US-8940744	ChEMBL	16	0.78	0.91	0.94	0.87	-2.36	-0.069
US-8940748	ChEMBL	10	0.56	0.92	0.92	0.78	-1.777	-0.024
US-8940748	SureChEMBLccs	500	0.15	0.56	0.71	0.39	0.913	0.436
US-8940748	SureChEMBL	1250	0.06	0.21	0.8	0.22	1.622	-0.992
US-8940771	SureChEMBL	648	0.05	0.28	0.73	0.22	1.753	-0.361
US-8940771	SureChEMBLccs	101	0.5	0.7	0.75	0.64	-0.425	0.645
US-8940771	ChEMBL	22	0.6	0.65	0.88	0.7	-1.059	-0.311
US-8940893	SureChEMBLccs	26	0.75	0.76	0.92	0.81	-1.845	-0.272
US-8940893	SureChEMBL	112	0.06	0.36	0.66	0.24	1.813	0.302
US-8940893	ChEMBL	17	0.76	0.8	0.94	0.83	-2.045	-0.318
US-8952008	SureChEMBLccs	56	0.66	0.71	0.83	0.73	-1.15	0.196
US-8952008	SureChEMBL	458	0.06	0.15	0.82	0.19	1.688	-1.26
US-8952008	ChEMBL	47	0.67	0.67	0.84	0.72	-1.117	0.045
US-8952025	SureChEMBL	274	0.05	0.28	0.44	0.18	2.904	1.646
US-8952025	SureChEMBLccs	103	0.3	0.59	0.74	0.51	0.362	0.372
US-8952025	ChEMBL	3	0.77	0.77	0.98	0.83	-2.155	-0.655
US-8952027	SureChEMBLccs	959	0.46	0.56	0.78	0.59	-0.108	0.113
US-8952027	ChEMBL	3	0.71	0.71	0.98	0.79	-1.865	-0.816
US-8952027	SureChEMBL	1858	0.05	0.42	0.59	0.23	1.969	0.911
US-8952036	ChEMBL	3	0.9	0.9	0.98	0.93	-2.782	-0.305
US-8952036	SureChEMBL	322	0.06	0.19	0.74	0.2	1.908	-0.62
US-8952036	SureChEMBLccs	15	0.5	0.5	0.94	0.62	-0.693	-1.103
US-8952037	SureChEMBLccs	486	0.37	0.56	0.71	0.53	0.386	0.551
US-8952037	ChEMBL	85	0.55	0.61	0.8	0.65	-0.525	0.13
US-8952037	SureChEMBL	976	0.05	0.62	0.43	0.24	2.119	2.452
US-8952169	ChEMBL	394	0.43	0.56	0.71	0.56	0.242	0.582
US-8952169	SureChEMBLccs	785	0.25	0.56	0.6	0.44	1.11	1.25
US-8952169	SureChEMBL	1397	0.05	0.41	0.48	0.21	2.43	1.651
US-8952177	ChEMBL	159	0.54	0.81	0.71	0.68	-0.629	1.181
US-8952177	SureChEMBLccs	373	0.5	0.73	0.66	0.62	-0.141	1.333
US-8952177	SureChEMBL	674	0.04	0.71	0.45	0.23	1.845	2.503
US-8956589	ChEMBL	17	0.69	0.87	0.9	0.81	-1.888	0.074
US-8956589	SureChEMBL	88	0.08	0.12	0.91	0.21	1.356	-1.938
US-8956589	SureChEMBLccs	50	0.67	0.84	0.8	0.77	-1.371	0.691
US-8957059	SureChEMBLccs	75	0.51	0.74	0.72	0.65	-0.427	0.944
US-8957059	ChEMBL	41	0.62	0.84	0.83	0.76	-1.37	0.457
US-8957059	SureChEMBL	272	0.04	0.28	0.71	0.2	1.857	-0.228
US-8957064	SureChEMBLccs	108	0.54	0.61	0.71	0.62	-0.144	0.747
US-8957064	SureChEMBL	549	0.04	0.23	0.77	0.19	1.74	-0.751
US-8957064	ChEMBL	83	0.65	0.72	0.79	0.72	-0.992	0.489
US-8957068	ChEMBL	491	0.39	0.56	0.76	0.55	0.139	0.215
US-8957068	SureChEMBLccs	849	0.35	0.56	0.71	0.52	0.434	0.54
US-8957068	SureChEMBL	1479	0.05	0.28	0.6	0.2	2.269	0.539

US-8957073	SureChEMBLccs	863	0.38	0.56	0.66	0.52	0.56	0.902
US-8957073	ChEMBL	372	0.49	0.74	0.79	0.66	-0.657	0.45
US-8957073	SureChEMBL	2174	0.06	0.22	0.8	0.22	1.597	-0.97
US-8957074	ChEMBL	137	0.67	0.77	0.81	0.75	-1.241	0.47
US-8957074	SureChEMBL	777	0.05	0.31	0.59	0.21	2.236	0.673
US-8957074	SureChEMBLccs	268	0.51	0.67	0.7	0.62	-0.178	0.931
US-8957075	ChEMBL	20	0.62	0.69	0.88	0.72	-1.204	-0.214
US-8957075	SureChEMBL	562	0.04	0.14	0.85	0.17	1.641	-1.5
US-8957075	SureChEMBLccs	223	0.56	0.72	0.71	0.66	-0.459	0.996
US-8957077	ChEMBL	14	0.55	0.64	0.92	0.69	-1.074	-0.636
US-8957077	SureChEMBLccs	75	0.49	0.68	0.78	0.64	-0.471	0.389
US-8957077	SureChEMBL	394	0.05	0.21	0.78	0.2	1.725	-0.858
US-8957093	SureChEMBL	1252	0.07	0.19	0.86	0.23	1.408	-1.445
US-8957093	ChEMBL	232	0.62	0.82	0.78	0.73	-1.123	0.76
US-8957093	SureChEMBLccs	731	0.57	0.77	0.76	0.69	-0.802	0.764
US-8957103	SureChEMBLccs	6	0.83	0.83	0.95	0.87	-2.326	-0.286
US-8957103	SureChEMBL	136	0.08	0.23	0.75	0.24	1.723	-0.592
US-8957103	ChEMBL	4	0.85	0.85	0.97	0.89	-2.501	-0.371
US-8957219	SureChEMBLccs	671	0.35	0.47	0.71	0.49	0.652	0.345
US-8957219	SureChEMBL	1032	0.06	0.28	0.8	0.24	1.452	-0.84
US-8957219	ChEMBL	12	0.59	0.88	0.9	0.78	-1.673	0.044
US-8962608	SureChEMBLccs	1034	0.37	0.65	0.79	0.57	-0.15	0.192
US-8962608	SureChEMBL	1472	0.06	0.21	0.85	0.22	1.423	-1.338
US-8962608	ChEMBL	520	0.64	0.7	0.8	0.71	-0.959	0.372
US-8962609	SureChEMBLccs	95	0.67	0.8	0.82	0.76	-1.353	0.466
US-8962609	SureChEMBL	153	0.06	0.24	0.73	0.22	1.827	-0.442
US-8962609	ChEMBL	72	0.67	0.8	0.83	0.76	-1.393	0.396
US-8962611	SureChEMBLccs	770	0.38	0.63	0.76	0.57	-0.007	0.362
US-8962611	SureChEMBL	1692	0.04	0.19	0.81	0.18	1.678	-1.115
US-8962611	ChEMBL	231	0.4	0.71	0.78	0.61	-0.328	0.407
US-8962612	SureChEMBL	428	0.12	0.44	0.64	0.32	1.554	0.645
US-8962612	ChEMBL	19	0.66	0.7	0.9	0.75	-1.404	-0.31
US-8962612	SureChEMBLccs	301	0.34	0.45	0.62	0.46	1.081	0.92
US-8962616	ChEMBL	10	0.7	0.81	0.96	0.82	-2.005	-0.466
US-8962616	SureChEMBL	416	0.06	0.21	0.76	0.21	1.78	-0.715
US-8962616	SureChEMBLccs	75	0.52	0.82	0.77	0.69	-0.843	0.777
US-8962619	SureChEMBL	488	0.06	0.15	0.82	0.19	1.688	-1.26
US-8962619	SureChEMBLccs	91	0.67	0.68	0.86	0.73	-1.221	-0.071
US-8962619	ChEMBL	17	0.76	0.76	0.92	0.81	-1.869	-0.266
US-8962630	ChEMBL	83	0.77	0.84	0.85	0.82	-1.809	0.397
US-8962630	SureChEMBL	324	0.06	0.77	0.56	0.3	1.215	1.883
US-8962630	SureChEMBLccs	112	0.74	0.82	0.84	0.8	-1.649	0.407
US-8962637	SureChEMBLccs	115	0.54	0.66	0.75	0.64	-0.424	0.579
US-8962637	ChEMBL	16	0.67	0.68	0.92	0.75	-1.459	-0.487
US-8962637	SureChEMBL	413	0.06	0.22	0.81	0.22	1.558	-1.039
US-8962641	SureChEMBLccs	512	0.57	0.75	0.76	0.69	-0.754	0.72
US-8962641	SureChEMBL	833	0.07	0.64	0.56	0.29	1.506	1.606
US-8962641	ChEMBL	450	0.57	0.72	0.84	0.7	-0.998	0.102
US-8962646	ChEMBL	11	0.79	0.86	0.94	0.86	-2.263	-0.173
US-8962646	SureChEMBLccs	36	0.56	0.69	0.88	0.7	-1.06	-0.245
US-8962646	SureChEMBL	202	0.07	0.22	0.82	0.23	1.494	-1.103

US-8962648	ChEMBL	223	0.52	0.74	0.69	0.64	-0.332	1.157
US-8962648	SureChEMBL	1993	0.09	0.18	0.86	0.24	1.384	-1.456
US-8962648	SureChEMBLccs	1711	0.38	0.56	0.73	0.54	0.282	0.418
US-8962651	SureChEMBL	199	0.06	0.19	0.82	0.21	1.591	-1.173
US-8962651	SureChEMBLccs	48	0.66	0.67	0.88	0.73	-1.252	-0.237
US-8962651	ChEMBL	2	0.92	0.92	0.99	0.94	-2.919	-0.321
US-8962674	SureChEMBL	1110	0.08	0.28	0.83	0.26	1.285	-1.037
US-8962674	SureChEMBLccs	802	0.5	0.72	0.75	0.65	-0.473	0.688
US-8962674	ChEMBL	88	0.55	0.73	0.76	0.67	-0.657	0.667
US-8962837	SureChEMBL	180	0.08	0.18	0.77	0.22	1.765	-0.839
US-8962837	ChEMBL	15	0.68	0.72	0.9	0.76	-1.5	-0.256
US-8962837	SureChEMBLccs	26	0.56	0.62	0.86	0.67	-0.811	-0.259
US-8962859	ChEMBL	11	0.83	0.84	0.98	0.88	-2.469	-0.472
US-8962859	SureChEMBLccs	26	0.64	0.83	0.95	0.8	-1.87	-0.385
US-8962859	SureChEMBL	117	0.08	0.19	0.82	0.23	1.543	-1.163
US-8969325	SureChEMBLccs	327	0.5	0.56	0.71	0.58	0.074	0.618
US-8969325	SureChEMBL	598	0.06	0.21	0.86	0.22	1.384	-1.407
US-8969325	ChEMBL	325	0.5	0.56	0.74	0.59	-0.045	0.411
US-8969333	ChEMBL	4	0.79	0.79	0.97	0.85	-2.212	-0.532
US-8969333	SureChEMBL	288	0.05	0.18	0.75	0.19	1.917	-0.716
US-8969333	SureChEMBLccs	21	0.41	0.56	0.87	0.58	-0.345	-0.536
US-8969335	SureChEMBLccs	125	0.53	0.78	0.76	0.68	-0.731	0.765
US-8969335	ChEMBL	77	0.64	0.72	0.81	0.72	-1.047	0.346
US-8969335	SureChEMBL	599	0.05	0.14	0.75	0.17	2.014	-0.803
US-8969336	SureChEMBL	1388	0.05	0.28	0.39	0.18	3.102	1.992
US-8969336	SureChEMBLccs	905	0.35	0.56	0.63	0.5	0.751	1.094
US-8969336	ChEMBL	34	0.64	0.82	0.85	0.76	-1.449	0.286
US-8969341	ChEMBL	185	0.56	0.59	0.76	0.63	-0.342	0.369
US-8969341	SureChEMBL	901	0.04	0.16	0.38	0.13	3.457	1.796
US-8969341	SureChEMBLccs	307	0.43	0.55	0.67	0.54	0.424	0.837
US-8969348	SureChEMBL	123	0.14	0.31	0.54	0.29	2.218	1.066
US-8969348	ChEMBL	10	0.67	0.67	0.91	0.74	-1.395	-0.439
US-8969348	SureChEMBLccs	48	0.57	0.62	0.78	0.65	-0.518	0.3
US-8969352	SureChEMBLccs	185	0.55	0.78	0.76	0.69	-0.779	0.775
US-8969352	SureChEMBL	396	0.05	0.28	0.76	0.22	1.634	-0.568
US-8969352	ChEMBL	46	0.65	0.75	0.82	0.74	-1.184	0.347
US-8969358	SureChEMBLccs	93	0.63	0.73	0.88	0.74	-1.325	-0.122
US-8969358	SureChEMBL	545	0.06	0.28	0.81	0.24	1.412	-0.909
US-8969358	ChEMBL	35	0.8	0.84	0.9	0.85	-2.079	0.066
US-8969376	SureChEMBLccs	288	0.5	0.72	0.7	0.63	-0.275	1.034
US-8969376	ChEMBL	43	0.61	0.72	0.84	0.72	-1.094	0.123
US-8969376	SureChEMBL	549	0.06	0.28	0.75	0.23	1.65	-0.494
US-8969394	ChEMBL	22	0.6	0.69	0.86	0.71	-1.077	-0.086
US-8969394	SureChEMBL	170	0.06	0.28	0.76	0.23	1.61	-0.563
US-8969394	SureChEMBLccs	46	0.41	0.49	0.73	0.53	0.38	0.281
US-8969578	SureChEMBL	37	0.07	0.32	0.84	0.27	1.172	-1.025
US-8969578	ChEMBL	4	0.82	0.82	0.97	0.87	-2.357	-0.451
US-8969578	SureChEMBLccs	12	0.68	0.75	0.92	0.78	-1.652	-0.33
US-8969583	ChEMBL	24	0.52	0.56	0.85	0.63	-0.53	-0.34
US-8969583	SureChEMBL	180	0.05	0.2	0.39	0.16	3.296	1.819
US-8969583	SureChEMBLccs	97	0.4	0.55	0.72	0.54	0.298	0.475

US-8975247	ChEMBL	27	0.45	0.65	0.87	0.63	-0.66	-0.32
US-8975247	SureChEMBLccs	304	0.41	0.53	0.63	0.52	0.68	1.06
US-8975247	SureChEMBL	983	0.05	0.15	0.79	0.18	1.831	-1.058
US-8975249	ChEMBL	36	0.72	0.85	0.85	0.8	-1.713	0.392
US-8975249	SureChEMBLccs	75	0.69	0.78	0.82	0.76	-1.353	0.433
US-8975249	SureChEMBL	343	0.06	0.17	0.79	0.2	1.758	-1.009
US-8975250	SureChEMBLccs	179	0.57	0.71	0.77	0.68	-0.696	0.565
US-8975250	SureChEMBL	371	0.05	0.17	0.51	0.16	2.893	0.923
US-8975250	ChEMBL	13	0.64	0.64	0.94	0.73	-1.369	-0.727
US-8975252	SureChEMBLccs	883	0.34	0.56	0.66	0.5	0.656	0.881
US-8975252	ChEMBL	2	0.78	0.78	0.99	0.84	-2.243	-0.697
US-8975252	SureChEMBL	1365	0.09	0.56	0.37	0.27	2.406	2.758
US-8975260	ChEMBL	12	0.7	0.85	0.92	0.82	-1.943	-0.103
US-8975260	SureChEMBLccs	43	0.63	0.76	0.8	0.73	-1.081	0.496
US-8975260	SureChEMBL	492	0.05	0.16	0.76	0.18	1.926	-0.828
US-8975261	SureChEMBL	503	0.07	0.28	0.67	0.24	1.943	0.065
US-8975261	SureChEMBLccs	166	0.33	0.57	0.8	0.53	0.1	-0.071
US-8975261	ChEMBL	15	0.62	0.71	0.91	0.74	-1.372	-0.378
US-8975265	SureChEMBL	691	0.05	0.13	0.87	0.18	1.562	-1.655
US-8975265	ChEMBL	179	0.75	0.8	0.87	0.81	-1.743	0.161
US-8975265	SureChEMBLccs	396	0.63	0.68	0.74	0.68	-0.649	0.738
US-8975267	SureChEMBLccs	106	0.31	0.34	0.72	0.42	1.024	-0.026
US-8975267	ChEMBL	15	0.39	0.39	0.92	0.52	-0.083	-1.26
US-8975267	SureChEMBL	262	0.07	0.28	0.57	0.22	2.34	0.757
US-8975282	SureChEMBLccs	59	0.15	0.75	0.76	0.44	0.254	0.502
US-8975282	SureChEMBL	371	0.04	0.28	0.75	0.2	1.698	-0.504
US-8975282	ChEMBL	4	0.86	0.86	0.97	0.9	-2.55	-0.344
US-8975417	SureChEMBL	603	0.07	0.38	0.56	0.25	2.137	1.043
US-8975417	SureChEMBLccs	260	0.47	0.65	0.67	0.59	0.086	1.075
US-8975417	ChEMBL	191	0.52	0.64	0.74	0.63	-0.288	0.594
US-8980887	ChEMBL	7	0.77	0.89	0.95	0.87	-2.327	-0.187
US-8980887	SureChEMBL	138	0.05	0.2	0.64	0.19	2.305	0.089
US-8980887	SureChEMBLccs	62	0.57	0.64	0.86	0.68	-0.884	-0.21
US-8980901	ChEMBL	5	0.72	0.8	0.97	0.82	-2.068	-0.547
US-8980901	SureChEMBLccs	122	0.63	0.81	0.76	0.73	-1.043	0.882
US-8980901	SureChEMBL	337	0.05	0.25	0.72	0.21	1.866	-0.357
US-8980904	SureChEMBLccs	100	0.53	0.55	0.77	0.61	-0.212	0.197
US-8980904	ChEMBL	18	0.64	0.67	0.93	0.74	-1.402	-0.593
US-8980904	SureChEMBL	382	0.06	0.17	0.53	0.18	2.79	0.79
US-8980905	SureChEMBL	107	0.07	0.17	0.85	0.22	1.496	-1.419
US-8980905	SureChEMBLccs	26	0.63	0.76	0.85	0.74	-1.279	0.15
US-8980905	ChEMBL	7	0.64	0.64	0.95	0.73	-1.409	-0.796
US-8980916	SureChEMBL	277	0.08	0.26	0.63	0.24	2.127	0.304
US-8980916	ChEMBL	3	0.85	0.85	0.98	0.89	-2.541	-0.44
US-8980916	SureChEMBLccs	16	0.36	0.42	0.9	0.51	-0.004	-1.073
US-8980929	SureChEMBL	397	0.07	0.2	0.76	0.22	1.781	-0.731
US-8980929	ChEMBL	7	0.78	0.78	0.96	0.84	-2.124	-0.489
US-8980929	SureChEMBLccs	26	0.37	0.38	0.82	0.49	0.386	-0.601
US-8981106	SureChEMBLccs	26	0.42	0.45	0.88	0.55	-0.142	-0.838
US-8981106	ChEMBL	9	0.72	0.72	0.92	0.78	-1.676	-0.374
US-8981106	SureChEMBL	513	0.05	0.17	0.75	0.19	1.941	-0.738

US-8987239	SureChEMBLccs	92	0.51	0.75	0.75	0.66	-0.57	0.758
US-8987239	SureChEMBL	147	0.06	0.34	0.76	0.25	1.465	-0.433
US-8987239	ChEMBL	8	0.82	0.83	0.96	0.87	-2.341	-0.36
US-8987249	SureChEMBL	1240	0.06	0.71	0.36	0.25	2.154	3.137
US-8987249	ChEMBL	356	0.61	0.83	0.77	0.73	-1.084	0.845
US-8987249	SureChEMBLccs	757	0.31	0.56	0.7	0.5	0.569	0.589
US-8987254	SureChEMBL	159	0.07	0.15	0.85	0.21	1.545	-1.462
US-8987254	SureChEMBLccs	61	0.56	0.71	0.87	0.7	-1.069	-0.133
US-8987254	ChEMBL	6	0.78	0.78	0.95	0.83	-2.084	-0.42
US-8987257	ChEMBL	34	0.67	0.72	0.86	0.75	-1.318	0.015
US-8987257	SureChEMBL	366	0.07	0.23	0.73	0.23	1.827	-0.459
US-8987257	SureChEMBLccs	87	0.65	0.72	0.82	0.73	-1.111	0.282
US-8987268	ChEMBL	52	0.56	0.56	0.84	0.64	-0.586	-0.25
US-8987268	SureChEMBL	1864	0.06	0.22	0.46	0.18	2.946	1.383
US-8987268	SureChEMBLccs	850	0.18	0.56	0.64	0.4	1.119	0.936
US-8987273	ChEMBL	38	0.77	0.8	0.92	0.83	-1.99	-0.175
US-8987273	SureChEMBLccs	88	0.65	0.76	0.81	0.74	-1.168	0.438
US-8987273	SureChEMBL	338	0.04	0.19	0.73	0.18	1.996	-0.561
US-8987286	ChEMBL	45	0.77	0.82	0.88	0.82	-1.88	0.146
US-8987286	SureChEMBLccs	83	0.64	0.81	0.82	0.75	-1.305	0.472
US-8987286	SureChEMBL	378	0.06	0.2	0.82	0.21	1.567	-1.152
US-8987314	SureChEMBL	938	0.05	0.28	0.78	0.22	1.555	-0.707
US-8987314	ChEMBL	255	0.49	0.78	0.76	0.66	-0.635	0.744
US-8987314	SureChEMBLccs	376	0.48	0.79	0.65	0.63	-0.199	1.521
US-8987319	ChEMBL	37	0.31	0.69	0.78	0.55	-0.064	0.317
US-8987319	SureChEMBLccs	108	0.28	0.42	0.73	0.44	0.862	0.062
US-8987319	SureChEMBL	245	0.04	0.1	0.74	0.14	2.175	-0.825
US-8987335	SureChEMBLccs	83	0.63	0.81	0.83	0.75	-1.321	0.397
US-8987335	ChEMBL	16	0.62	0.8	0.86	0.75	-1.392	0.163
US-8987335	SureChEMBL	233	0.05	0.28	0.77	0.22	1.595	-0.638
US-8987457	ChEMBL	169	0.56	0.71	0.74	0.67	-0.553	0.767
US-8987457	SureChEMBLccs	273	0.4	0.68	0.66	0.56	0.221	1.172
US-8987457	SureChEMBL	574	0.05	0.4	0.49	0.21	2.414	1.56
US-8987474	SureChEMBLccs	167	0.47	0.68	0.78	0.63	-0.423	0.378
US-8987474	SureChEMBL	322	0.09	0.66	0.41	0.29	2.005	2.698
US-8987474	ChEMBL	140	0.56	0.68	0.72	0.65	-0.401	0.84
US-8993552	SureChEMBLccs	46	0.37	0.37	0.79	0.48	0.529	-0.415
US-8993552	ChEMBL	34	0.39	0.62	0.79	0.58	-0.125	0.137
US-8993552	SureChEMBL	115	0.08	0.21	0.82	0.24	1.494	-1.12
US-8993556	SureChEMBLccs	273	0.28	0.69	0.62	0.49	0.643	1.408
US-8993556	SureChEMBL	430	0.05	0.73	0.28	0.22	2.447	3.728
US-8993556	ChEMBL	16	0.7	0.7	0.85	0.75	-1.301	0.057
US-8993557	SureChEMBL	321	0.07	0.28	0.7	0.24	1.824	-0.143
US-8993557	SureChEMBLccs	97	0.41	0.56	0.81	0.57	-0.107	-0.121
US-8993557	ChEMBL	10	0.48	0.54	0.92	0.62	-0.663	-0.889
US-8993565	ChEMBL	151	0.56	0.83	0.74	0.7	-0.845	1.027
US-8993565	SureChEMBL	355	0.08	0.26	0.75	0.25	1.651	-0.527
US-8993565	SureChEMBLccs	158	0.56	0.83	0.74	0.7	-0.845	1.027
US-8993568	SureChEMBLccs	33	0.61	0.84	0.87	0.76	-1.505	0.175
US-8993568	ChEMBL	5	0.78	0.78	0.96	0.84	-2.124	-0.489
US-8993568	SureChEMBL	101	0.07	0.17	0.73	0.21	1.972	-0.589

US-8993575	ChEMBL	5	0.63	0.63	0.96	0.72	-1.4	-0.892
US-8993575	SureChEMBL	66	0.12	0.22	0.82	0.28	1.374	-1.077
US-8993575	SureChEMBLccs	22	0.65	0.66	0.87	0.72	-1.164	-0.194
US-8993586	SureChEMBLccs	101	0.36	0.56	0.68	0.52	0.529	0.753
US-8993586	SureChEMBL	518	0.05	0.23	0.77	0.21	1.716	-0.746
US-8993586	ChEMBL	24	0.67	0.75	0.9	0.77	-1.549	-0.196
US-8993612	SureChEMBL	260	0.05	0.16	0.86	0.19	1.529	-1.52
US-8993612	ChEMBL	4	0.66	0.66	0.97	0.75	-1.584	-0.881
US-8993612	SureChEMBLccs	22	0.67	0.67	0.93	0.75	-1.474	-0.577
US-8993616	ChEMBL	92	0.6	0.67	0.82	0.69	-0.87	0.147
US-8993616	SureChEMBLccs	291	0.4	0.68	0.68	0.57	0.141	1.034
US-8993616	SureChEMBL	554	0.07	0.19	0.79	0.22	1.686	-0.961
US-8993631	SureChEMBL	858	0.05	0.28	0.71	0.22	1.833	-0.222
US-8993631	ChEMBL	42	0.55	0.72	0.81	0.68	-0.831	0.299
US-8993631	SureChEMBLccs	475	0.36	0.56	0.72	0.53	0.37	0.476
US-8993756	SureChEMBL	540	0.07	0.25	0.81	0.24	1.461	-0.969
US-8993756	SureChEMBLccs	336	0.49	0.78	0.73	0.65	-0.516	0.951
US-8993756	ChEMBL	112	0.55	0.81	0.78	0.7	-0.931	0.702
US-8993765	SureChEMBLccs	72	0.44	0.62	0.72	0.58	0.032	0.648
US-8993765	ChEMBL	17	0.56	0.72	0.88	0.71	-1.133	-0.18
US-8993765	SureChEMBL	318	0.05	0.14	0.82	0.18	1.736	-1.287
US-8999957	SureChEMBLccs	332	0.6	0.71	0.76	0.69	-0.729	0.649
US-8999957	ChEMBL	265	0.6	0.69	0.76	0.68	-0.68	0.606
US-8999957	SureChEMBL	1054	0.05	0.18	0.81	0.19	1.679	-1.131
US-8999975	SureChEMBLccs	304	0.49	0.61	0.63	0.57	0.294	1.275
US-8999975	ChEMBL	136	0.65	0.78	0.84	0.75	-1.336	0.273
US-8999975	SureChEMBL	507	0.05	0.12	0.86	0.17	1.626	-1.607
US-8999980	ChEMBL	16	0.64	0.67	0.88	0.72	-1.204	-0.247
US-8999980	SureChEMBLccs	60	0.5	0.56	0.83	0.61	-0.402	-0.212
US-8999980	SureChEMBL	301	0.04	0.11	0.63	0.14	2.587	-0.042
US-8999981	ChEMBL	189	0.56	0.69	0.74	0.66	-0.505	0.724
US-8999981	SureChEMBLccs	285	0.47	0.69	0.67	0.6	-0.011	1.161
US-8999981	SureChEMBL	411	0.06	0.56	0.39	0.24	2.399	2.604
US-8999994	ChEMBL	7	0.88	0.88	0.96	0.91	-2.606	-0.221
US-8999994	SureChEMBLccs	9	0.68	0.68	0.94	0.76	-1.562	-0.62
US-8999994	SureChEMBL	52	0.12	0.28	0.88	0.31	0.99	-1.362
US-8999998	SureChEMBL	1524	0.04	0.19	0.8	0.18	1.718	-1.045
US-8999998	ChEMBL	15	0.64	0.67	0.92	0.73	-1.362	-0.524
US-8999998	SureChEMBLccs	550	0.5	0.8	0.73	0.66	-0.588	1.0
US-9000008	ChEMBL	5	0.72	0.73	0.97	0.8	-1.898	-0.698
US-9000008	SureChEMBLccs	13	0.54	0.59	0.95	0.67	-1.047	-0.957
US-9000008	SureChEMBL	71	0.04	0.1	0.73	0.14	2.214	-0.756
US-9000015	SureChEMBLccs	22	0.6	0.71	0.87	0.72	-1.165	-0.112
US-9000015	SureChEMBL	224	0.04	0.24	0.7	0.19	1.994	-0.245
US-9000015	ChEMBL	14	0.79	0.85	0.96	0.86	-2.318	-0.333
US-9000153	SureChEMBLccs	45	0.7	0.77	0.86	0.77	-1.511	0.139
US-9000153	SureChEMBL	111	0.08	0.28	0.78	0.26	1.483	-0.691
US-9000153	ChEMBL	41	0.7	0.77	0.87	0.78	-1.551	0.07
US-9000182	ChEMBL	6	0.75	0.75	0.97	0.82	-2.019	-0.639
US-9000182	SureChEMBL	155	0.07	0.41	0.7	0.27	1.509	0.139
US-9000182	SureChEMBLccs	68	0.41	0.83	0.85	0.66	-0.921	0.188

US-9000184	SureChEMBLccs	28	0.52	0.78	0.92	0.72	-1.341	-0.348
US-9000184	ChEMBL	17	0.66	0.81	0.93	0.79	-1.79	-0.279
US-9000184	SureChEMBL	77	0.08	0.34	0.78	0.28	1.338	-0.561
US-9000185	SureChEMBLccs	49	0.5	0.5	0.9	0.61	-0.535	-0.827
US-9000185	ChEMBL	2	0.97	0.97	0.99	0.98	-3.16	-0.187
US-9000185	SureChEMBL	110	0.07	0.2	0.86	0.23	1.384	-1.423
US-9006232	SureChEMBL	241	0.08	0.65	0.54	0.3	1.537	1.771
US-9006232	ChEMBL	70	0.59	0.73	0.75	0.69	-0.714	0.757
US-9006232	SureChEMBLccs	98	0.56	0.68	0.77	0.66	-0.6	0.494
US-9006242	SureChEMBL	150	0.07	0.12	0.67	0.18	2.332	-0.282
US-9006242	ChEMBL	2	0.63	0.63	0.99	0.73	-1.519	-1.1
US-9006242	SureChEMBLccs	22	0.53	0.67	0.92	0.69	-1.098	-0.581
US-9006244	ChEMBL	4	0.85	0.85	0.98	0.89	-2.541	-0.44
US-9006244	SureChEMBLccs	33	0.47	0.57	0.85	0.61	-0.434	-0.344
US-9006244	SureChEMBL	197	0.05	0.28	0.74	0.22	1.714	-0.43
US-9006246	ChEMBL	13	0.6	0.6	0.92	0.69	-1.096	-0.696
US-9006246	SureChEMBLccs	659	0.52	0.67	0.74	0.64	-0.36	0.659
US-9006246	SureChEMBL	994	0.06	0.2	0.8	0.21	1.646	-1.013
US-9006249	ChEMBL	10	0.64	0.82	0.92	0.78	-1.726	-0.199
US-9006249	SureChEMBL	872	0.06	0.28	0.78	0.24	1.531	-0.702
US-9006249	SureChEMBLccs	275	0.56	0.64	0.76	0.65	-0.463	0.477
US-9006268	ChEMBL	6	0.73	0.8	0.96	0.82	-2.052	-0.472
US-9006268	SureChEMBL	286	0.06	0.07	0.8	0.15	1.961	-1.295
US-9006268	SureChEMBLccs	57	0.35	0.48	0.76	0.5	0.429	0.021
US-9006269	SureChEMBLccs	28	0.56	0.64	0.85	0.67	-0.82	-0.146
US-9006269	ChEMBL	14	0.7	0.71	0.9	0.76	-1.524	-0.268
US-9006269	SureChEMBL	136	0.07	0.25	0.8	0.24	1.501	-0.9
US-9006282	SureChEMBL	63	0.12	0.21	0.76	0.27	1.636	-0.684
US-9006282	SureChEMBLccs	12	0.46	0.46	0.94	0.58	-0.5	-1.211
US-9006282	ChEMBL	8	0.46	0.78	0.94	0.7	-1.277	-0.518
US-9006454	SureChEMBLccs	172	0.46	0.56	0.73	0.57	0.09	0.459
US-9006454	ChEMBL	99	0.52	0.71	0.76	0.65	-0.537	0.608
US-9006454	SureChEMBL	842	0.04	0.12	0.78	0.16	1.967	-1.059
US-9012443	ChEMBL	482	0.5	0.7	0.69	0.62	-0.187	1.06
US-9012443	SureChEMBL	1361	0.05	0.15	0.88	0.19	1.474	-1.68
US-9012443	SureChEMBLccs	911	0.34	0.56	0.7	0.51	0.497	0.604
US-9012461	ChEMBL	37	0.69	0.84	0.84	0.79	-1.577	0.424
US-9012461	SureChEMBL	570	0.04	0.17	0.31	0.13	3.71	2.302
US-9012461	SureChEMBLccs	192	0.56	0.71	0.68	0.65	-0.315	1.182
US-9012475	ChEMBL	12	0.83	0.83	0.95	0.87	-2.326	-0.286
US-9012475	SureChEMBL	124	0.05	0.21	0.84	0.21	1.487	-1.274
US-9012475	SureChEMBLccs	37	0.61	0.67	0.92	0.72	-1.29	-0.539
US-9012651	ChEMBL	209	0.46	0.71	0.69	0.61	-0.115	1.061
US-9012651	SureChEMBL	810	0.06	0.56	0.57	0.27	1.685	1.358
US-9012651	SureChEMBLccs	476	0.41	0.69	0.66	0.57	0.172	1.199
US-9018211	SureChEMBLccs	214	0.34	0.56	0.74	0.52	0.338	0.328
US-9018211	SureChEMBL	611	0.07	0.2	0.81	0.22	1.582	-1.077
US-9018211	ChEMBL	9	0.66	0.74	0.96	0.78	-1.739	-0.639
US-9018213	SureChEMBLccs	20	0.77	0.78	0.87	0.81	-1.743	0.128
US-9018213	ChEMBL	3	0.74	0.74	0.98	0.81	-2.01	-0.735
US-9018213	SureChEMBL	84	0.12	0.22	0.84	0.28	1.295	-1.216

US-9018214	SureChEMBLccs	806	0.23	0.56	0.62	0.43	1.078	1.101
US-9018214	ChEMBL	217	0.47	0.56	0.8	0.59	-0.211	-0.02
US-9018214	SureChEMBL	1522	0.06	0.56	0.47	0.25	2.081	2.05
US-9018217	ChEMBL	90	0.53	0.65	0.75	0.64	-0.375	0.552
US-9018217	SureChEMBLccs	193	0.28	0.56	0.7	0.48	0.641	0.573
US-9018217	SureChEMBL	454	0.05	0.56	0.46	0.23	2.145	2.114
US-9018255	SureChEMBLccs	8	0.41	0.66	0.96	0.64	-0.945	-0.942
US-9018255	SureChEMBL	43	0.12	0.21	0.8	0.27	1.478	-0.96
US-9018255	ChEMBL	3	0.66	0.66	0.98	0.75	-1.624	-0.95
US-9023849	SureChEMBL	332	0.07	0.15	0.72	0.2	2.061	-0.563
US-9023849	SureChEMBLccs	29	0.36	0.56	0.87	0.56	-0.225	-0.562
US-9023849	ChEMBL	6	0.81	0.81	0.96	0.86	-2.269	-0.409
US-9023865	SureChEMBLccs	711	0.42	0.56	0.71	0.55	0.266	0.577
US-9023865	ChEMBL	645	0.51	0.63	0.78	0.63	-0.398	0.291
US-9023865	SureChEMBL	1420	0.06	0.28	0.78	0.24	1.531	-0.702
US-9023882	ChEMBL	204	0.52	0.66	0.72	0.63	-0.257	0.776
US-9023882	SureChEMBL	800	0.07	0.22	0.77	0.23	1.692	-0.757
US-9023882	SureChEMBLccs	540	0.44	0.56	0.72	0.56	0.178	0.518
US-9029356	SureChEMBLccs	197	0.38	0.56	0.67	0.52	0.52	0.833
US-9029356	SureChEMBL	494	0.06	0.23	0.58	0.2	2.446	0.574
US-9029356	ChEMBL	4	0.84	0.85	0.98	0.89	-2.517	-0.445
US-9029360	SureChEMBLccs	24	0.63	0.8	0.88	0.76	-1.495	0.029
US-9029360	SureChEMBL	169	0.06	0.19	0.7	0.2	2.067	-0.343
US-9029360	ChEMBL	18	0.7	0.73	0.93	0.78	-1.692	-0.432
US-9029362	SureChEMBLccs	419	0.43	0.56	0.76	0.57	0.043	0.236
US-9029362	ChEMBL	7	0.8	0.86	0.95	0.87	-2.326	-0.237
US-9029362	SureChEMBL	1223	0.06	0.21	0.8	0.22	1.622	-0.992
US-9029367	ChEMBL	3	0.78	0.78	0.98	0.84	-2.203	-0.628
US-9029367	SureChEMBL	323	0.06	0.28	0.74	0.23	1.69	-0.425
US-9029367	SureChEMBLccs	198	0.63	0.69	0.78	0.7	-0.832	0.483
US-9029381	ChEMBL	25	0.74	0.76	0.85	0.78	-1.543	0.208
US-9029381	SureChEMBLccs	84	0.74	0.84	0.85	0.81	-1.737	0.381
US-9029381	SureChEMBL	497	0.05	0.12	0.84	0.17	1.705	-1.469
US-9029393	ChEMBL	18	0.55	0.65	0.87	0.68	-0.899	-0.268
US-9029393	SureChEMBLccs	179	0.38	0.56	0.67	0.52	0.52	0.833
US-9029393	SureChEMBL	385	0.11	0.18	0.46	0.21	2.923	1.322
US-9029399	ChEMBL	50	0.65	0.74	0.87	0.75	-1.358	-0.021
US-9029399	SureChEMBLccs	67	0.6	0.76	0.83	0.72	-1.128	0.273
US-9029399	SureChEMBL	395	0.07	0.18	0.76	0.21	1.829	-0.775
US-9029559	SureChEMBL	433	0.07	0.35	0.78	0.27	1.337	-0.545
US-9029559	SureChEMBLccs	348	0.57	0.73	0.72	0.67	-0.547	0.954
US-9029559	ChEMBL	162	0.63	0.85	0.78	0.75	-1.22	0.83
US-9034574	SureChEMBLccs	5	0.34	0.34	0.97	0.48	-0.04	-1.741
US-9034574	SureChEMBL	61	0.12	0.15	0.88	0.25	1.306	-1.644
US-9034574	ChEMBL	2	0.76	0.76	0.99	0.83	-2.146	-0.751
US-9034856	SureChEMBLccs	62	0.63	0.74	0.87	0.74	-1.31	-0.031
US-9034856	SureChEMBL	164	0.05	0.15	0.88	0.19	1.474	-1.68
US-9034856	ChEMBL	7	0.79	0.79	0.95	0.84	-2.132	-0.393
US-9034874	ChEMBL	10	0.66	0.84	0.94	0.8	-1.902	-0.283
US-9034874	SureChEMBL	163	0.05	0.16	0.78	0.18	1.846	-0.967
US-9034874	SureChEMBLccs	13	0.64	0.83	0.91	0.78	-1.711	-0.108

US-9034886	ChEMBL	5	0.61	0.61	0.96	0.71	-1.303	-0.946
US-9034886	SureChEMBL	105	0.11	0.2	0.87	0.27	1.248	-1.472
US-9034886	SureChEMBLccs	60	0.59	0.61	0.82	0.67	-0.7	0.012
US-9034897	SureChEMBLccs	116	0.68	0.92	0.81	0.8	-1.629	0.8
US-9034897	SureChEMBL	200	0.06	0.13	0.92	0.19	1.34	-1.995
US-9034897	ChEMBL	41	0.72	0.85	0.85	0.8	-1.713	0.392
US-9034899	SureChEMBL	248	0.07	0.24	0.78	0.24	1.604	-0.783
US-9034899	ChEMBL	24	0.59	0.72	0.85	0.71	-1.086	0.043
US-9034899	SureChEMBLccs	71	0.49	0.63	0.7	0.6	-0.033	0.834
US-9034921	SureChEMBLccs	381	0.27	0.49	0.69	0.45	0.875	0.485
US-9034921	SureChEMBL	893	0.06	0.39	0.6	0.24	1.978	0.782
US-9034921	ChEMBL	199	0.57	0.76	0.72	0.68	-0.619	1.019
US-9035066	ChEMBL	6	0.55	0.55	0.95	0.66	-0.974	-1.038
US-9035066	SureChEMBLccs	66	0.4	0.54	0.8	0.56	0.005	-0.1
US-9035066	SureChEMBL	134	0.08	0.25	0.47	0.21	2.786	1.389
US-9035074	ChEMBL	27	0.68	0.76	0.86	0.76	-1.439	0.107
US-9035074	SureChEMBL	321	0.06	0.23	0.81	0.22	1.533	-1.018
US-9035074	SureChEMBLccs	120	0.49	0.54	0.81	0.6	-0.251	-0.122
US-9040501	ChEMBL	4	0.75	0.75	0.98	0.82	-2.058	-0.708
US-9040501	SureChEMBLccs	17	0.43	0.56	0.95	0.61	-0.711	-1.079
US-9040501	SureChEMBL	153	0.07	0.19	0.84	0.22	1.488	-1.307
US-9040518	SureChEMBLccs	287	0.62	0.76	0.78	0.72	-0.977	0.63
US-9040518	SureChEMBL	430	0.1	0.83	0.62	0.37	0.735	1.618
US-9040518	ChEMBL	69	0.72	0.84	0.87	0.81	-1.768	0.232
US-9040528	SureChEMBL	956	0.06	0.44	0.42	0.22	2.571	2.136
US-9040528	SureChEMBLccs	704	0.33	0.82	0.62	0.55	0.208	1.716
US-9040528	ChEMBL	186	0.69	0.75	0.85	0.76	-1.399	0.16
US-9040534	SureChEMBLccs	122	0.56	0.74	0.79	0.69	-0.825	0.486
US-9040534	ChEMBL	90	0.61	0.84	0.8	0.74	-1.227	0.659
US-9040534	SureChEMBL	324	0.06	0.16	0.84	0.2	1.584	-1.377
US-9040691	ChEMBL	27	0.54	0.75	0.88	0.71	-1.158	-0.126
US-9040691	SureChEMBL	208	0.08	0.42	0.6	0.27	1.857	0.858
US-9040691	SureChEMBLccs	56	0.5	0.7	0.79	0.65	-0.583	0.368
US-9040694	ChEMBL	5	0.74	0.74	0.96	0.81	-1.931	-0.597
US-9040694	SureChEMBL	46	0.09	0.25	0.82	0.26	1.373	-1.028
US-9040694	SureChEMBLccs	9	0.64	0.78	0.94	0.78	-1.709	-0.424
US-9040714	SureChEMBL	946	0.04	0.18	0.8	0.18	1.742	-1.067
US-9040714	SureChEMBLccs	289	0.37	0.56	0.72	0.53	0.346	0.482
US-9040714	ChEMBL	94	0.45	0.56	0.82	0.59	-0.243	-0.169
US-9040727	SureChEMBL	98	0.13	0.31	0.84	0.32	1.052	-1.015
US-9040727	ChEMBL	18	0.73	0.76	0.91	0.8	-1.757	-0.213
US-9040727	SureChEMBLccs	59	0.64	0.83	0.8	0.75	-1.275	0.653
US-9045389	SureChEMBLccs	19	0.69	0.73	0.89	0.77	-1.509	-0.16
US-9045389	ChEMBL	9	0.75	0.82	0.93	0.83	-2.03	-0.211
US-9045389	SureChEMBL	187	0.05	0.13	0.63	0.16	2.514	0.006
US-9045459	ChEMBL	3	0.79	0.79	0.99	0.85	-2.291	-0.67
US-9045459	SureChEMBLccs	12	0.65	0.74	0.9	0.76	-1.477	-0.229
US-9045459	SureChEMBL	287	0.1	0.28	0.79	0.28	1.395	-0.75
US-9045468	SureChEMBLccs	136	0.47	0.59	0.76	0.6	-0.126	0.322
US-9045468	SureChEMBL	300	0.07	0.2	0.76	0.22	1.781	-0.731
US-9045468	ChEMBL	48	0.6	0.7	0.82	0.7	-0.942	0.212

US-9045483	SureChEMBLccs	24	0.56	0.67	0.87	0.69	-0.972	-0.219
US-9045483	SureChEMBL	118	0.05	0.26	0.78	0.22	1.604	-0.75
US-9045483	ChEMBL	13	0.57	0.57	0.91	0.67	-0.912	-0.708
US-9045498	SureChEMBLccs	70	0.49	0.69	0.74	0.63	-0.337	0.687
US-9045498	SureChEMBL	468	0.04	0.2	0.75	0.18	1.892	-0.678
US-9045498	ChEMBL	10	0.59	0.59	0.92	0.68	-1.048	-0.723
US-9045500	SureChEMBL	311	0.07	0.15	0.81	0.2	1.704	-1.186
US-9045500	ChEMBL	16	0.71	0.73	0.93	0.78	-1.716	-0.427
US-9045500	SureChEMBLccs	36	0.65	0.86	0.83	0.77	-1.49	0.516
US-9045501	ChEMBL	21	0.46	0.55	0.92	0.62	-0.639	-0.877
US-9045501	SureChEMBLccs	149	0.32	0.49	0.7	0.48	0.715	0.442
US-9045501	SureChEMBL	545	0.06	0.21	0.81	0.22	1.582	-1.061
US-9051240	SureChEMBL	266	0.06	0.23	0.43	0.18	3.041	1.612
US-9051240	ChEMBL	2	0.85	0.85	0.99	0.89	-2.581	-0.509
US-9051240	SureChEMBLccs	51	0.51	0.79	0.75	0.67	-0.667	0.845
US-9051265	SureChEMBLccs	442	0.53	0.85	0.68	0.67	-0.583	1.47
US-9051265	SureChEMBL	807	0.05	0.28	0.45	0.18	2.864	1.577
US-9051265	ChEMBL	61	0.56	0.65	0.89	0.69	-1.003	-0.401
US-9051270	SureChEMBL	802	0.06	0.16	0.85	0.2	1.545	-1.446
US-9051270	SureChEMBLccs	238	0.49	0.69	0.69	0.62	-0.139	1.033
US-9051270	ChEMBL	156	0.63	0.81	0.77	0.73	-1.083	0.812
US-9051279	ChEMBL	58	0.61	0.72	0.84	0.72	-1.094	0.123
US-9051279	SureChEMBLccs	723	0.33	0.7	0.72	0.55	0.102	0.764
US-9051279	SureChEMBL	1872	0.06	0.28	0.4	0.19	3.039	1.928
US-9051280	SureChEMBLccs	315	0.45	0.56	0.63	0.54	0.511	1.146
US-9051280	SureChEMBL	631	0.07	0.28	0.72	0.24	1.745	-0.281
US-9051280	ChEMBL	10	0.75	0.76	0.94	0.81	-1.924	-0.41
US-9051311	SureChEMBLccs	134	0.44	0.68	0.77	0.61	-0.312	0.432
US-9051311	ChEMBL	59	0.62	0.85	0.79	0.75	-1.235	0.755
US-9051311	SureChEMBL	262	0.05	0.16	0.83	0.19	1.648	-1.313
US-9051313	ChEMBL	17	0.78	0.79	0.89	0.82	-1.87	0.017
US-9051313	SureChEMBL	315	0.07	0.2	0.75	0.22	1.82	-0.662
US-9051313	SureChEMBLccs	41	0.59	0.84	0.85	0.75	-1.377	0.303
US-9051321	ChEMBL	27	0.56	0.56	0.82	0.64	-0.507	-0.112
US-9051321	SureChEMBLccs	195	0.43	0.77	0.73	0.62	-0.347	0.899
US-9051321	SureChEMBL	551	0.07	0.1	0.69	0.17	2.301	-0.464
US-9051329	ChEMBL	8	0.77	0.77	0.93	0.82	-1.957	-0.309
US-9051329	SureChEMBLccs	43	0.46	0.56	0.75	0.58	0.011	0.321
US-9051329	SureChEMBL	306	0.05	0.28	0.76	0.22	1.634	-0.568
US-9056832	SureChEMBLccs	305	0.48	0.68	0.72	0.62	-0.209	0.799
US-9056832	ChEMBL	150	0.51	0.56	0.73	0.59	-0.03	0.485
US-9056832	SureChEMBL	1043	0.07	0.28	0.79	0.25	1.467	-0.766
US-9056836	ChEMBL	3	0.7	0.7	0.98	0.78	-1.817	-0.843
US-9056836	SureChEMBLccs	71	0.33	0.45	0.83	0.5	0.272	-0.539
US-9056836	SureChEMBL	449	0.08	0.26	0.68	0.24	1.928	-0.042
US-9056843	SureChEMBLccs	208	0.46	0.63	0.72	0.59	-0.04	0.68
US-9056843	ChEMBL	139	0.61	0.74	0.76	0.7	-0.826	0.72
US-9056843	SureChEMBL	466	0.05	0.22	0.76	0.2	1.78	-0.698
US-9056857	SureChEMBLccs	36	0.31	0.5	0.86	0.51	0.08	-0.649
US-9056857	ChEMBL	5	0.8	0.8	0.96	0.85	-2.22	-0.436
US-9056857	SureChEMBL	595	0.06	0.18	0.77	0.2	1.813	-0.849

US-9056859	SureChEMBL	1255	0.07	0.36	0.7	0.26	1.63	0.031
US-9056859	SureChEMBLccs	526	0.33	0.56	0.65	0.49	0.72	0.945
US-9056859	ChEMBL	9	0.69	0.74	0.93	0.78	-1.692	-0.415
US-9056863	SureChEMBL	125	0.06	0.19	0.84	0.21	1.512	-1.312
US-9056863	SureChEMBLccs	44	0.35	0.44	0.79	0.5	0.407	-0.273
US-9056863	ChEMBL	3	0.77	0.77	0.98	0.83	-2.155	-0.655
US-9056865	SureChEMBL	452	0.04	0.18	0.49	0.15	2.972	1.078
US-9056865	SureChEMBLccs	181	0.45	0.56	0.75	0.57	0.035	0.316
US-9056865	ChEMBL	148	0.47	0.63	0.71	0.59	-0.024	0.754
US-9061041	ChEMBL	6	0.32	0.32	0.95	0.46	0.136	-1.656
US-9061041	SureChEMBLccs	107	0.28	0.34	0.72	0.41	1.096	-0.042
US-9061041	SureChEMBL	341	0.05	0.28	0.43	0.18	2.944	1.715
US-9062004	ChEMBL	18	0.64	0.68	0.89	0.73	-1.268	-0.295
US-9062004	SureChEMBLccs	26	0.57	0.64	0.86	0.68	-0.884	-0.21
US-9062004	SureChEMBL	109	0.06	0.24	0.82	0.23	1.47	-1.065
US-9062045	ChEMBL	89	0.6	0.8	0.79	0.72	-1.066	0.637
US-9062045	SureChEMBL	654	0.07	0.16	0.78	0.21	1.798	-0.956
US-9062045	SureChEMBLccs	229	0.36	0.56	0.63	0.5	0.727	1.099
US-9062048	SureChEMBL	991	0.06	0.79	0.38	0.26	1.88	3.172
US-9062048	ChEMBL	127	0.63	0.79	0.85	0.75	-1.352	0.215
US-9062048	SureChEMBLccs	553	0.43	0.78	0.67	0.61	-0.134	1.335
US-9062059	SureChEMBL	652	0.05	0.28	0.68	0.21	1.952	-0.015
US-9062059	ChEMBL	20	0.62	0.65	0.86	0.7	-1.028	-0.162
US-9062059	SureChEMBLccs	230	0.52	0.82	0.68	0.66	-0.486	1.4
US-9062060	ChEMBL	11	0.73	0.81	0.95	0.83	-2.037	-0.381
US-9062060	SureChEMBL	194	0.04	0.19	0.76	0.18	1.877	-0.769
US-9062060	SureChEMBLccs	72	0.3	0.42	0.78	0.46	0.616	-0.273
US-9062070	SureChEMBL	792	0.06	0.28	0.56	0.21	2.404	0.821
US-9062070	SureChEMBLccs	312	0.33	0.51	0.7	0.49	0.642	0.491
US-9062070	ChEMBL	34	0.74	0.83	0.88	0.81	-1.832	0.152
US-9062078	SureChEMBLccs	531	0.36	0.56	0.7	0.52	0.449	0.615
US-9062078	SureChEMBL	882	0.04	0.37	0.54	0.2	2.313	1.144
US-9062078	ChEMBL	264	0.49	0.56	0.74	0.59	-0.021	0.406
US-9066954	SureChEMBLccs	109	0.5	0.72	0.76	0.65	-0.513	0.619
US-9066954	SureChEMBL	935	0.06	0.16	0.78	0.2	1.822	-0.962
US-9066954	ChEMBL	19	0.61	0.7	0.91	0.73	-1.323	-0.405
US-9067871	ChEMBL	158	0.5	0.56	0.79	0.6	-0.244	0.065
US-9067871	SureChEMBLccs	507	0.47	0.56	0.69	0.57	0.225	0.741
US-9067871	SureChEMBL	941	0.08	0.28	0.76	0.26	1.562	-0.553
US-9067917	SureChEMBLccs	278	0.16	0.72	0.77	0.45	0.263	0.373
US-9067917	SureChEMBL	584	0.04	0.68	0.5	0.24	1.719	2.092
US-9067917	ChEMBL	136	0.64	0.73	0.78	0.71	-0.953	0.575
US-9067922	SureChEMBL	340	0.04	0.21	0.79	0.19	1.709	-0.933
US-9067922	ChEMBL	66	0.75	0.81	0.86	0.81	-1.728	0.252
US-9067922	SureChEMBLccs	98	0.75	0.81	0.84	0.8	-1.649	0.39
US-9067931	SureChEMBL	346	0.11	0.28	0.82	0.29	1.252	-0.952
US-9067931	ChEMBL	2	0.91	0.91	0.99	0.94	-2.87	-0.348
US-9067931	SureChEMBLccs	82	0.74	0.8	0.85	0.8	-1.64	0.294
US-9067935	SureChEMBL	216	0.07	0.21	0.79	0.23	1.637	-0.917
US-9067935	ChEMBL	8	0.6	0.6	0.96	0.7	-1.255	-0.973
US-9067935	SureChEMBLccs	52	0.33	0.39	0.72	0.45	0.854	0.092

US-9067937	ChEMBL	94	0.56	0.62	0.77	0.64	-0.454	0.364
US-9067937	SureChEMBLccs	391	0.46	0.77	0.61	0.6	0.057	1.745
US-9067937	SureChEMBL	725	0.07	0.28	0.71	0.24	1.785	-0.212
US-9073833	SureChEMBL	58	0.14	0.26	0.79	0.31	1.348	-0.773
US-9073833	ChEMBL	8	0.83	0.83	0.95	0.87	-2.326	-0.286
US-9073833	SureChEMBLccs	24	0.77	0.83	0.92	0.84	-2.063	-0.11
US-9073853	ChEMBL	151	0.59	0.72	0.76	0.69	-0.729	0.666
US-9073853	SureChEMBL	450	0.1	0.68	0.63	0.35	1.06	1.224
US-9073853	SureChEMBLccs	357	0.49	0.65	0.71	0.61	-0.121	0.808
US-9073869	ChEMBL	25	0.74	0.76	0.94	0.81	-1.9	-0.415
US-9073869	SureChEMBL	250	0.06	0.12	0.81	0.18	1.8	-1.256
US-9073869	SureChEMBLccs	56	0.56	0.61	0.89	0.67	-0.906	-0.488
US-9073870	ChEMBL	95	0.45	0.65	0.81	0.62	-0.422	0.095
US-9073870	SureChEMBLccs	464	0.28	0.41	0.73	0.44	0.886	0.041
US-9073870	SureChEMBL	728	0.05	0.28	0.75	0.22	1.674	-0.499
US-9073876	SureChEMBL	748	0.07	0.19	0.82	0.22	1.567	-1.168
US-9073876	SureChEMBLccs	421	0.47	0.71	0.73	0.62	-0.298	0.789
US-9073876	ChEMBL	306	0.49	0.7	0.79	0.65	-0.56	0.363
US-9073881	SureChEMBL	556	0.05	0.07	0.92	0.15	1.509	-2.131
US-9073881	ChEMBL	140	0.69	0.83	0.81	0.77	-1.434	0.61
US-9073881	SureChEMBLccs	239	0.65	0.82	0.77	0.74	-1.155	0.844
US-9073893	ChEMBL	24	0.52	0.63	0.87	0.66	-0.779	-0.327
US-9073893	SureChEMBL	139	0.04	0.19	0.78	0.18	1.798	-0.907
US-9073893	SureChEMBLccs	76	0.43	0.77	0.74	0.63	-0.387	0.829
US-9073906	SureChEMBL	586	0.07	0.12	0.73	0.18	2.094	-0.697
US-9073906	SureChEMBLccs	447	0.28	0.77	0.52	0.48	0.846	2.274
US-9073906	ChEMBL	22	0.6	0.67	0.91	0.72	-1.227	-0.475
US-9073917	SureChEMBLccs	18	0.69	0.72	0.87	0.76	-1.405	-0.043
US-9073917	ChEMBL	7	0.72	0.72	0.94	0.79	-1.755	-0.512
US-9073917	SureChEMBL	56	0.12	0.28	0.76	0.29	1.466	-0.532
US-9073922	SureChEMBLccs	595	0.39	0.56	0.67	0.53	0.496	0.838
US-9073922	SureChEMBL	1257	0.05	0.18	0.81	0.19	1.679	-1.131
US-9073922	ChEMBL	352	0.42	0.56	0.63	0.53	0.583	1.13
US-9073925	SureChEMBLccs	33	0.56	0.68	0.82	0.68	-0.798	0.148
US-9073925	ChEMBL	10	0.72	0.75	0.96	0.8	-1.907	-0.586
US-9073925	SureChEMBL	210	0.05	0.16	0.86	0.19	1.529	-1.52
US-9073926	ChEMBL	104	0.51	0.59	0.76	0.61	-0.222	0.343
US-9073926	SureChEMBLccs	137	0.34	0.57	0.68	0.51	0.552	0.764
US-9073926	SureChEMBL	280	0.06	0.21	0.52	0.19	2.732	0.946
US-9073931	SureChEMBL	543	0.05	0.23	0.7	0.2	1.994	-0.261
US-9073931	ChEMBL	48	0.76	0.78	0.91	0.81	-1.878	-0.154
US-9073931	SureChEMBLccs	208	0.56	0.69	0.77	0.67	-0.624	0.516
US-9073939	SureChEMBLccs	13	0.62	0.83	0.89	0.77	-1.584	0.02
US-9073939	SureChEMBL	267	0.04	0.19	0.76	0.18	1.877	-0.769
US-9073939	ChEMBL	4	0.72	0.83	0.98	0.84	-2.181	-0.551
US-9079852	SureChEMBLccs	758	0.32	0.39	0.79	0.46	0.601	-0.397
US-9079852	SureChEMBL	1018	0.06	0.67	0.42	0.26	2.013	2.635
US-9079852	ChEMBL	10	0.78	0.86	0.95	0.86	-2.278	-0.247
US-9079866	SureChEMBLccs	1187	0.46	0.72	0.67	0.61	-0.06	1.221
US-9079866	ChEMBL	787	0.49	0.68	0.7	0.62	-0.154	0.942
US-9079866	SureChEMBL	2152	0.05	0.19	0.82	0.2	1.615	-1.179

US-9079880	SureChEMBLccs	215	0.37	0.56	0.68	0.52	0.505	0.758
US-9079880	ChEMBL	100	0.53	0.65	0.74	0.63	-0.336	0.621
US-9079880	SureChEMBL	459	0.08	0.62	0.45	0.28	1.967	2.329
US-9079906	ChEMBL	27	0.55	0.63	0.81	0.65	-0.613	0.104
US-9079906	SureChEMBL	1011	0.07	0.56	0.36	0.24	2.494	2.817
US-9079906	SureChEMBLccs	416	0.49	0.56	0.73	0.59	0.018	0.475
US-9079913	SureChEMBLccs	254	0.55	0.74	0.7	0.66	-0.444	1.104
US-9079913	SureChEMBL	863	0.07	0.15	0.48	0.17	3.013	1.098
US-9079913	ChEMBL	95	0.62	0.68	0.76	0.68	-0.704	0.595
US-9085531	ChEMBL	16	0.6	0.6	0.91	0.69	-1.057	-0.627
US-9085531	SureChEMBL	1927	0.06	0.17	0.9	0.21	1.322	-1.77
US-9085531	SureChEMBLccs	1372	0.42	0.56	0.72	0.55	0.226	0.508
US-9085540	SureChEMBL	895	0.08	0.13	0.84	0.21	1.609	-1.431
US-9085540	SureChEMBLccs	634	0.4	0.56	0.75	0.55	0.155	0.29
US-9085540	ChEMBL	17	0.57	0.81	0.89	0.74	-1.415	-0.049
US-9085549	SureChEMBLccs	47	0.27	0.66	0.7	0.5	0.422	0.785
US-9085549	ChEMBL	10	0.6	0.6	0.94	0.7	-1.176	-0.835
US-9085549	SureChEMBL	271	0.07	0.2	0.83	0.23	1.503	-1.216
US-9085555	ChEMBL	678	0.44	0.54	0.77	0.57	0.028	0.129
US-9085555	SureChEMBL	2108	0.06	0.28	0.62	0.22	2.166	0.406
US-9085555	SureChEMBLccs	1367	0.25	0.43	0.56	0.39	1.584	1.245
US-9085572	ChEMBL	10	0.65	0.65	0.91	0.73	-1.298	-0.493
US-9085572	SureChEMBL	214	0.06	0.28	0.65	0.22	2.047	0.198
US-9085572	SureChEMBLccs	84	0.35	0.65	0.77	0.56	-0.023	0.32
US-9085576	SureChEMBLccs	715	0.3	0.56	0.67	0.48	0.712	0.791
US-9085576	ChEMBL	344	0.54	0.73	0.72	0.66	-0.475	0.938
US-9085576	SureChEMBL	1078	0.07	0.18	0.81	0.22	1.631	-1.121
US-9085584	SureChEMBLccs	307	0.38	0.56	0.64	0.51	0.639	1.04
US-9085584	SureChEMBL	641	0.06	0.14	0.82	0.19	1.712	-1.282
US-9085584	ChEMBL	121	0.59	0.67	0.76	0.67	-0.608	0.557
US-9089569	SureChEMBLccs	371	0.5	0.73	0.66	0.62	-0.141	1.333
US-9089569	ChEMBL	159	0.53	0.81	0.7	0.67	-0.565	1.245
US-9089569	SureChEMBL	659	0.04	0.66	0.39	0.22	2.204	2.81
US-9090562	SureChEMBL	792	0.06	0.65	0.44	0.26	1.982	2.453
US-9090562	ChEMBL	144	0.68	0.74	0.8	0.74	-1.152	0.479
US-9090562	SureChEMBLccs	279	0.55	0.7	0.67	0.64	-0.227	1.225
US-9090596	SureChEMBLccs	89	0.69	0.81	0.81	0.77	-1.386	0.567
US-9090596	ChEMBL	36	0.72	0.79	0.89	0.8	-1.726	-0.015
US-9090596	SureChEMBL	323	0.09	0.25	0.69	0.25	1.889	-0.128
US-9090618	SureChEMBLccs	294	0.67	0.81	0.86	0.78	-1.536	0.21
US-9090618	ChEMBL	116	0.73	0.78	0.81	0.77	-1.409	0.523
US-9090618	SureChEMBL	954	0.06	0.23	0.77	0.22	1.692	-0.741
US-9090628	ChEMBL	51	0.61	0.67	0.85	0.7	-1.013	-0.055
US-9090628	SureChEMBL	1125	0.05	0.23	0.78	0.21	1.676	-0.815
US-9090628	SureChEMBLccs	302	0.49	0.56	0.73	0.59	0.018	0.475
US-9096579	SureChEMBLccs	217	0.37	0.74	0.71	0.58	-0.051	0.941
US-9096579	ChEMBL	174	0.67	0.78	0.84	0.76	-1.384	0.284
US-9096579	SureChEMBL	852	0.07	0.18	0.81	0.22	1.631	-1.121
US-9096580	ChEMBL	5	0.84	0.84	0.96	0.88	-2.413	-0.328
US-9096580	SureChEMBL	73	0.07	0.17	0.78	0.21	1.774	-0.935
US-9096580	SureChEMBLccs	16	0.72	0.84	0.92	0.82	-1.967	-0.114

US-9096589	SureChEMBLccs	102	0.5	0.56	0.77	0.6	-0.164	0.203
US-9096589	ChEMBL	80	0.5	0.71	0.75	0.64	-0.449	0.667
US-9096589	SureChEMBL	323	0.05	0.23	0.71	0.2	1.954	-0.331
US-9096593	ChEMBL	54	0.61	0.8	0.78	0.72	-1.05	0.711
US-9096593	SureChEMBL	1337	0.06	0.16	0.8	0.2	1.743	-1.1
US-9096593	SureChEMBLccs	654	0.32	0.44	0.74	0.47	0.678	0.057
US-9096594	ChEMBL	4	0.84	0.84	0.97	0.88	-2.453	-0.397
US-9096594	SureChEMBLccs	180	0.53	0.86	0.74	0.7	-0.845	1.076
US-9096594	SureChEMBL	623	0.06	0.15	0.8	0.19	1.767	-1.122
US-9096595	ChEMBL	49	0.68	0.74	0.84	0.75	-1.311	0.202
US-9096595	SureChEMBL	193	0.08	0.18	0.85	0.23	1.448	-1.392
US-9096595	SureChEMBLccs	83	0.52	0.56	0.77	0.61	-0.212	0.214
US-9096606	SureChEMBL	543	0.08	0.27	0.81	0.26	1.388	-0.92
US-9096606	ChEMBL	14	0.85	0.88	0.94	0.89	-2.455	-0.098
US-9096606	SureChEMBLccs	101	0.55	0.76	0.82	0.7	-0.968	0.316
US-9102591	SureChEMBLccs	340	0.28	0.56	0.61	0.46	0.998	1.196
US-9102591	SureChEMBL	666	0.08	0.18	0.63	0.21	2.321	0.13
US-9102591	ChEMBL	168	0.43	0.56	0.77	0.57	0.004	0.167
US-9102656	SureChEMBLccs	10	0.78	0.84	0.95	0.85	-2.23	-0.29
US-9102656	ChEMBL	6	0.77	0.77	0.95	0.83	-2.036	-0.447
US-9102656	SureChEMBL	50	0.12	0.21	0.81	0.27	1.438	-1.03
US-9102670	SureChEMBLccs	516	0.62	0.84	0.75	0.73	-1.052	1.011
US-9102670	ChEMBL	423	0.62	0.84	0.75	0.73	-1.052	1.011
US-9102670	SureChEMBL	907	0.05	0.65	0.5	0.25	1.768	2.033
US-9102686	ChEMBL	45	0.7	0.8	0.88	0.79	-1.663	0.066
US-9102686	SureChEMBLccs	264	0.56	0.64	0.69	0.63	-0.185	0.961
US-9102686	SureChEMBL	388	0.05	0.35	0.78	0.24	1.385	-0.555
US-9107923	SureChEMBLccs	97	0.39	0.52	0.83	0.55	-0.041	-0.356
US-9107923	SureChEMBL	537	0.04	0.18	0.8	0.18	1.742	-1.067
US-9107923	ChEMBL	72	0.61	0.64	0.81	0.68	-0.781	0.157
US-9108903	SureChEMBL	614	0.06	0.15	0.84	0.2	1.609	-1.398
US-9108903	ChEMBL	60	0.63	0.7	0.78	0.7	-0.856	0.505
US-9108903	SureChEMBLccs	103	0.56	0.74	0.72	0.67	-0.547	0.97
US-9108905	SureChEMBL	202	0.06	0.16	0.84	0.2	1.584	-1.377
US-9108905	ChEMBL	34	0.65	0.76	0.89	0.76	-1.486	-0.116
US-9108905	SureChEMBLccs	74	0.47	0.76	0.85	0.67	-0.895	0.067
US-9108951	SureChEMBL	689	0.04	0.28	0.74	0.2	1.738	-0.435
US-9108951	ChEMBL	10	0.77	0.78	0.94	0.83	-2.021	-0.356
US-9108951	SureChEMBLccs	154	0.33	0.55	0.81	0.53	0.109	-0.184
US-9108973	SureChEMBLccs	125	0.73	0.76	0.83	0.77	-1.44	0.341
US-9108973	SureChEMBL	349	0.06	0.4	0.64	0.25	1.795	0.527
US-9108973	ChEMBL	57	0.71	0.81	0.85	0.79	-1.592	0.3
US-9114126	SureChEMBLccs	4	0.71	0.71	0.97	0.79	-1.826	-0.747
US-9114126	SureChEMBL	38	0.09	0.13	0.89	0.22	1.387	-1.772
US-9114126	ChEMBL	4	0.71	0.71	0.97	0.79	-1.826	-0.747
US-9115102	SureChEMBLccs	135	0.39	0.51	0.73	0.53	0.38	0.314
US-9115102	SureChEMBL	240	0.08	0.13	0.84	0.21	1.609	-1.431
US-9115102	ChEMBL	20	0.71	0.82	0.88	0.8	-1.736	0.114
US-9115117	SureChEMBLccs	318	0.35	0.55	0.7	0.51	0.497	0.588
US-9115117	SureChEMBL	499	0.06	0.21	0.76	0.21	1.78	-0.715
US-9115117	ChEMBL	97	0.56	0.69	0.75	0.66	-0.545	0.654

US-9115121	ChEMBL	36	0.67	0.75	0.87	0.76	-1.43	0.011
US-9115121	SureChEMBLccs	51	0.65	0.75	0.84	0.74	-1.263	0.208
US-9115121	SureChEMBL	181	0.08	0.28	0.68	0.25	1.88	0.001
US-9115126	SureChEMBL	410	0.08	0.28	0.62	0.24	2.118	0.416
US-9115126	ChEMBL	10	0.64	0.8	0.94	0.78	-1.757	-0.381
US-9115126	SureChEMBLccs	293	0.48	0.74	0.68	0.62	-0.196	1.206
US-9115140	ChEMBL	19	0.66	0.79	0.9	0.78	-1.622	-0.115
US-9115140	SureChEMBL	670	0.07	0.28	0.59	0.23	2.261	0.619
US-9115140	SureChEMBLccs	277	0.34	0.72	0.56	0.52	0.664	1.92
US-9120745	SureChEMBL	943	0.06	0.18	0.78	0.2	1.774	-0.918
US-9120745	SureChEMBLccs	49	0.38	0.39	0.78	0.49	0.496	-0.297
US-9120745	ChEMBL	6	0.66	0.66	0.95	0.75	-1.505	-0.743
US-9120749	SureChEMBLccs	1948	0.39	0.66	0.64	0.55	0.373	1.262
US-9120749	ChEMBL	598	0.51	0.65	0.7	0.61	-0.129	0.888
US-9120749	SureChEMBL	2545	0.09	0.38	0.51	0.26	2.288	1.399
US-9120756	SureChEMBLccs	131	0.49	0.73	0.7	0.63	-0.275	1.051
US-9120756	ChEMBL	39	0.63	0.82	0.82	0.75	-1.306	0.488
US-9120756	SureChEMBL	442	0.1	0.28	0.77	0.28	1.475	-0.612
US-9120761	ChEMBL	13	0.78	0.83	0.93	0.84	-2.126	-0.174
US-9120761	SureChEMBL	332	0.05	0.28	0.43	0.18	2.944	1.715
US-9120761	SureChEMBLccs	44	0.61	0.68	0.84	0.7	-0.997	0.036
US-9238653	SureChEMBLccs	80	0.57	0.65	0.76	0.66	-0.511	0.504
US-9238653	SureChEMBL	211	0.06	0.23	0.74	0.22	1.811	-0.533
US-9238653	ChEMBL	29	0.63	0.76	0.85	0.74	-1.279	0.15
